# Supplementary material for: Key factors for connecting silver-based icosahedral superatoms by vertex sharing
Source: Commun Chem. 2023 Mar 28;6:57. doi: 10.1038/s42004-023-00854-0 (PMC10050180; doi:10.1038/s42004-023-00854-0)
Supplement: Supplementary file 2 — Supplemental Information [file 42004_2023_854_MOESM2_ESM.pdf]

Supporting Information

# ***Key factors for connecting silver-based icosahedral superatoms by vertex sharing***

Sayuri Miyajima,[1] Sakiat Hossain,[2,*] Ayaka Ikeda,[1] Taiga Kosaka,[1] Tokuhisa Kawawaki,[1,2] Yoshiki Niihori,[2] Takeshi Iwasa,[*,3,4], Tetsuya Taketsugu,[3,4] and Yuichi Negishi[*,1,2]

[1]Department of Applied Chemistry, Faculty of Science, Tokyo University of Science, Kagurazaka, Shinjuku−ku, Tokyo 162−8601, Japan

[2]Research Institute for Science & Technology, Tokyo University of Science, Kagurazaka, Shinjuku−ku, Tokyo 162−8601, Japan

[3]Department of Chemistry, Faculty of Science, Hokkaido University, Sapporo, Hokkaido, 060−0810, Japan

[4]WPI-ICReDD, Hokkaido University, Sapporo, Hokkaido, 060−0810, Japan

Corresponding Author E-mail: sakiathossain@rs.tus.ac.jp (S. Hossain); tiwasa@sci.hokudai.ac.jp (T. Iwasa); negishi@rs.tus.ac.jp (Y. Negishi)

## S1. Supplementary Methods

### S1.1 Chemicals:

All chemicals were obtained commercially and used without further purification. Silver nitrate ($AgNO_3$), methanol, ethanol, dichloromethane, and hexane were purchased from Kanto Chemical Co., Inc. Triphenylphosphine ($PPh_3$), tetraoctylammonium bromide (TOABr), sodium tetrahydroborate ($NaBH_4$), Ag standard solution (1.00 mg/mL), Pd standard solution (1.00 mg/mL), and yttrium (Y) standard solution (1.00 mg/mL) were purchased from FUJIFILM Wako Pure Chemical Co. Tetrakis(triphenylphosphine)palladium ($Pd(PPh_3)_4$), platinum(II) bromide ($PtBr_2$) were purchased from Sigma Aldrich. Palladium(II) bromide ($PdBr_2$) and cesium carbonate ($Cs_2CO_3$) were purchased from Tokyo Chemical Industry. Chloroplatinic acid hexahydrate ($H_2PtCl_6 \cdot 6H_2O$) was purchased from Tanaka Kikinzoku. Indium (In) plate was purchased from the Nilaco Corporation. Pure Milli-Q water (18.2 MΩ cm) was generated with a Merck Millipore Direct 3 UV system.

### S1.2 Experiments and Software Used for Obtaining Geometric Structures:

The single crystal was immersed in cryoprotectant Parabar 10312 (Hampton Research, 34 Journey, Aliso Viejo, CA 92656-3317 USA) and mounted on a MicroLoops E Inclined Assortment ™ (MiTeGen, LLC, Ithaca, NY, USA). The X-ray diffraction data sets were collected using a Bruker D8 QUEST, using monochromated MoKα radiation ($\lambda$ = 0.71073 Å). Bruker Apex 3[1] suite was used for solving preliminary structures by following the sequential steps: indexing, data integration, reduction, absorption correction (multi-scan), space group determination, structure solution (with intrinsic-Phasing method). Even after numerous attempts using crystals of multiple batches over six months, we could not get diffraction having resolution better than 1.10 Å. Although heavy atoms and many carbon atoms could be solved by shelxt using intrinsic phasing method, structures were completed by subsequent refinement using full-matrix least squares method against $F^2$ by SHELXL-2018/3 in Olex2 GUI.[2,3] Disordered six membered phenyl rings are fixed by using several restrain and constrains, such as RIGU, SIMU and AFIX 66. Few carbon atoms were refined with isotropic displacement parameters as their thermal parameters are quite high to refine anisotropically. Finally, H-atoms are refined with a riding model by placing them in calculated positions. Although the sample **4** has two Pd, one at each of the central core position of an icosahedral of the bi-icosahedral structure, they could not be assigned crystallographically as both Ag and Pd have similar total electron number, so their presence in the cluster confirmed by other methods, such as X-ray photoelectron spectroscopy (XPS) and inductively coupled plasma mass spectroscopy.

### S1.3 Additional Information on Crystal Analysis:

#### S1.3.1 Details of 3 (CCDC 2195306):

**Fixed Uiso**

At 1.2 times of: All C(H) groups

**Uiso/Uaniso restraints and constraints**

C2 ≈ C3 ≈ C4 ≈ C5 ≈ C6 ≈ C7 ≈ C8 ≈ C9 ≈ C10 ≈ C12 ≈ C13 ≈ C14 ≈ C15 ≈ C16 ≈ C17 ≈ C19 ≈ C20 ≈ C21≈ C22 ≈ C23 ≈ C24 ≈ C25 ≈ C26 ≈ C27 ≈ C28≈ C29 ≈ C30 ≈ C31 ≈ C32 ≈ C33 ≈ C34 ≈ C36 ≈ C37≈ C38 ≈ C39 ≈ C40 ≈ C41 ≈ C42 ≈ C43 ≈ C44 ≈ C46≈ C47 ≈ C48 ≈ C49 ≈ C50 ≈ C51 ≈ C52 ≈ C53 ≈ C54≈ C55 ≈ C56 ≈ C57 ≈ C58 ≈ C59 ≈ C60 ≈ C61 ≈ C62≈ C63 ≈ C64 ≈ C65 ≈ C68 ≈ C69 ≈ C70 ≈ C71 ≈ C72≈ C73 ≈ C74 ≈ C75 ≈ C76 ≈ C77 ≈ C78 ≈ C79 ≈ C80≈ C81 ≈ C82 ≈ C83 ≈ C84 ≈ C86 ≈ C87 ≈ C88 ≈ C90≈ C91 ≈ C92 ≈ C93 ≈ C95 ≈ C96 ≈ C97 ≈ C98 ≈ C99≈ C100 ≈ C101 ≈ C102 ≈ C103 ≈ C104 ≈ C105 ≈ C106≈ C108 ≈ C109 ≈ C111 ≈ C113 ≈ C115 ≈ C119 ≈ C121≈ C122 ≈ C123 ≈ C124 ≈ C125 ≈ C127 ≈ C129 ≈ C131≈ C133 ≈ C135 ≈ C137 ≈ C138 ≈ C139 ≈ C140 ≈ C141≈ C142 ≈ C143 ≈ C144 ≈ C145 ≈ C146 ≈ C147 ≈ C148≈ C149 ≈ C150 ≈ C151 ≈ C152 ≈ C153 ≈ C154 ≈ C155≈ C156 ≈ C158 ≈ C160 ≈ C161 ≈ C162 ≈ C163 ≈ C165≈ C166 ≈ C168 ≈ C169 ≈ C171 ≈ C175 ≈ C179: within 1.7A with sigma of 0.04 and sigma for terminal atoms of 0.08 within 1.7A: with sigma of 0.05 and sigma for terminal atoms of 0.1

**Rigid body (RIGU) restrains**

C2, C4, C5, C6, C7, C8, C9, C10, C12, C13, C14, C15, C16, C17, C19, C20, C21, C22, C23, C24, C25, C26, C27, C28, C29, C30, C31, C32, C33, C34, C36, C37, C38, C39, C40, C41, C42, C43, C44, C46, C47, C48, C49, C50, C51, C52, C53, C54, C55, C56, C57, C58, C59, C60, C61, C62, C63, C64, C65, C68, C69, C70, C71, C72, C73, C74, C75, C76, C77, C78, C79, C80, C81, C82, C83, C86, C87, C88, C90, C91, C92, C93, C95, C96, C97, C98, C99, C100, C101, C102, C103, C104, C105, C106, C108, C109, C111, C113, C115, C119, C121, C122, C123, C124, C125, C126, C127, C128, C129, C130, C131, C132, C133, C134, C135, C136, C137, C138, C139, C140, C141, C142, C143, C144, C145, C146, C147, C148, C149, C150, C151, C152, C153, C154, C155, C156, C158, C160, C161, C162, C163, C165, C166, C168, C169, C171, C175, C179, C3, C84 with sigma for 1-2 distances of 0.004 and sigma for 1-3 distances of 0.004

**Aromatic H refined with riding coordinates:**

C8(H8), C10(H10), C12(H12), C14(H14), C16(H16), C20(H20), C22(H22), C26(H26), C28(H28), C30(H30), C32(H32), C36(H36), C42(H42), C44(H44), C48(H48), C52(H52), C54(H54), C56(H56), C58(H58), C60(H60), C62(H62), C64(H64), C66(H66), C68(H68), C70(H70), C72(H72), C74(H74), C78(H78), C82(H82), C84(H84), C94(H94), C96(H96), C98(H98), C100(H100), C102(H102), C104(H104), C108(H108), C112(H112), C114(H114), C116(H116), C118(H118), C120(H120), C122(H122), C124(H124), C128(H128), C130(H130), C132(H132), C134(H134), C136(H136), C138(H138), C140(H140), C142(H142), C144(H144), C146(H146), C148(H148), C152(H152), C156(H156), C158(H158), C160(H160), C162(H162), C164(H164), C166(H166), C168(H168), C174(H174), C176(H176), C178(H178), C180(H180), C45(H45), C91(H91), C23(H23), C93(H93), C47(H47), C95(H95), C3(H3), C49(H49), C99(H99), C25(H25), C101(H101), C51(H51), C103(H103), C13(H13), C105(H105), C53(H53), C107(H107), C27(H27), C109(H109), C55(H55), C111(H111), C113(H113), C57(H57), C115(H115), C29(H29), C117(H117), C119(H119), C15(H15), C121(H121),C61(H61), C123(H123), C31(H31), C125(H125), C63(H63), C127(H127), C2(H2), C65(H65), C131(H131), C33(H33), C133(H133), C67(H67), C135(H135), C17(H17), C137(H137), C139(H139), C35(H35), C141(H141), C71(H71), C143(H143), C145(H145), C73(H73), C37(H37), C149(H149), C75(H75), C151(H151), C19(H19), C153(H153),C77(H77), C155(H155), C39(H39), C157(H157), C79(H79), C159(H159), C5(H5), C161(H161), C81(H81), C163(H163), C41(H41), C165(H165), C83(H83), C167(H167), C21(H21), C169(H169), C85(H85), C171(H171), C43(H43), C173(H173), C87(H87), C175(H175), C11(H11), C177(H177), C89(H89), C179(H179)

**Aromatic H refined with riding coordinates:**

**Fitted hexagon refined as free rotating group:**

C6 (C8, C10, C12, C14, C16), C110 (C112, C114, C116, C118, C120), C126 (C128, C130, C132, C134, C136), C172 (C174, C176, C178, C180, C45), C59 (C119, C15, C121, C61, C123), C31 (C125, C63, C127, C2, C129)

**S1.3.2 Details of 4 (CCDC 2195307):**

**Fixed Uiso**

At 1.2 times of: All C(H) groups

**Rigid bond restraints**

C22 with sigma for 1-2 distances of 0.01 and sigma for 1-3 distances of 0.01

**Uiso/Uaniso restraints and constraints**

C87 ≈ C175 ≈ C11 ≈ C177 ≈ C89 ≈ C179: within 1.7A with sigma of 0.04 and sigma for terminal atoms of 0.08 within 1.7A Uanis(C22) ≈ Ueq: with sigma of 0.005 and sigma for terminal atoms of 0.05

**Rigid body (RIGU) restrains**
C87, C175, C11, C177, C89, C179 with sigma for 1-2 distances of 0.004 and sigma for 1-3 distances of 0.004 C125, C163, C45, C27, C28, C178 with sigma for 1-2 distances of 0.002 and sigma for 1-3 distances of 0.002 C144, C172 with sigma for 1-2 distances of 0.001 and sigma for 1-3 distances of 0.0005 C22 with sigma for 1-2 distances of 0.001 and sigma for 1-3 distances of 0.0005

**Aromatic H refined with riding coordinates:**
C1(H1), C8(H8), C12(H12), C16(H16), C148(H148), C61(H61), C19(H19), C171(H171), C101(H101), C26(H26), C30(H30), C32(H32), C34(H34), C38(H38), C40(H40), C42(H42), C48(H48), C50(H50), C52(H52), C56(H56), C60(H60), C62(H62), C72(H72), C76(H76), C78(H78), C80(H80), C82(H82), C86(H86), C88(H88), C90(H90), C94(H94), C98(H98), C153(H153), C91(H91), C15(H15), C65(H65), C114(H114), C102(H102), C104(H104), C108(H108), C118(H118), C120(H120), C122(H122), C126(H126), C128(H128), C130(H130), C132(H132), C134(H134), C136(H136), C138(H138), C140(H140), C142(H142), C144(H144), C146(H146), C150(H150), C152(H152), C154(H154), C156(H156), C158(H158), C162(H162), C164(H164), C166(H166), C168(H168), C170(H170), C172(H172), C174(H174), C176(H176), C178(H178), C180(H180), C45(H45), C23(H23), C93(H93), C47(H47), C95(H95), C3(H3), C97(H97), C49(H49), C99(H99), C25(H25), C51(H51), C103(H103), C13(H13), C105(H105), C53(H53), C107(H107), C27(H27), C109(H109), C55(H55), C111(H111), C7(H7), C113(H113), C57(H57), C115(H115), C29(H29), C117(H117), C59(H59), C119(H119), C121(H121), C123(H123), C31(H31), C125(H125), C63(H63), C127(H127), C2(H2), C129(H129), C131(H131), C33(H33), C133(H133), C67(H67), C135(H135), C17(H17), C137(H137), C69(H69), C139(H139), C35(H35), C141(H141), C71(H71), C143(H143), C9(H9), C145(H145), C73(H73), C147(H147), C37(H37), C149(H149), C75(H75), C151(H151), C77(H77), C155(H155), C39(H39), C157(H157), C79(H79), C159(H159), C5(H5), C161(H161), C81(H81), C163(H163), C41(H41), C165(H165), C83(H83), C167(H167), C21(H21), C169(H169), C85(H85), C43(H43), C173(H173), C175(H175), C11(H11), C177(H177), C89(H89), C179(H179)

**Fitted hexagon refined as free rotating group:**
C22(C148,C61,C19,C171,C101), C100(C153,C91,C15,C65,C114), C124(C126,C128,C130, C132,C134). C87(C175,C11,C177,C89,C179)

## S2. Supplementary Results

### S2.1 Crystal Data:

#### S2.1.1 Data deposition information:

Deposition Numbers CCDC 2195306 (for **3** at 90.15 K), CCDC 2195307 (for **4** at 90.00 K) contain the supplementary crystallographic data for this paper. These data are provided free of charge by the joint Cambridge Crystallographic Data Centre and Fachinformationszentrum (FIZ) Karlsruhe. It can be accessed in following link: https://www.ccdc.cam.ac.uk/structures/

### S2.2 Important structural parameters and refinement details:

Crystal data and some of the important structural refinement parameters for sample **3** and **4** are given below. All the details of crystal data and structural refinement parameters are tabulated in Table S1.

**3 (CCDC 2195306):** $C_{180}H_{150}Ag_{23}Br_7P_{10}Pt_2$ ($M_w$ =6053.25 g/mol): monoclinic, space group $P2_1/m$ (no. 11), $a$ = 21.470(3) Å, $b$ = 29.041(5) Å, $c$ = 29.913(5) Å, $\beta$ = 102.403(4)°, $V$ = 18216(5) Å$^3$, $Z$ = 4, $T$ = 90.15 K, μ(MoKα) = 5.607 mm$^{-1}$, $Dcalc$ = 2.207 g/cm$^3$, 52781 reflections measured (3.884° ≤ 2θ ≤ 37.706°), 14202 unique ($R_{int}$ = 0.1563, $R_{sigma}$ = 0.1243) which were used in all calculations. The final $R_1$ was 0.0600 (I > 2σ(I)) and $wR_2$ was 0.1549 (all data).

**4 (CCDC 2195307):** $C_{180}H_{150}Ag_{23}Br_7P_{10}Pd_2$ ($M_w$ =5875.87 g/mol): monoclinic, space group $P2_1/m$ (no. 11), $a$ = 21.4359(6)Å, $b$ = 28.9930(8) Å, $c$ = 29.7998(8) Å, $\beta$ = 102.6710°, $V$ = 18069 (5) Å$^3$, $Z$ = 4, $T$ = 90.00 K, μ(MoKα) = 4.305 mm$^{-1}$, $Dcalc$ = 2.160 g/cm$^3$, 73984 reflections measured (3.894° ≤ 2θ ≤ 37.802), 14280 unique ($R_{int}$ = 0.0802, $R_{sigma}$ = 0.0507) which were used in all calculations. The final $R_1$ was 0.0351 (I > 2σ(I)) and $wR_2$ was 0.0843 (all data).

## S3 Additional Tables

**Table S1 Details of Crystal Data and Structure Refinement for 3 and 4**

| Sample | 3 | 4 |
|---|---|---|
| Empirical formula | $C_{180}H_{150}Ag_{23}Br_7P_{10}Pt_2$ | $C_{180}H_{150}Ag_{23}Br_7P_{10}Pd_2$ |
| CCDC number | 2195306 | 2195307 |
| Formula weight | 6053.25 | 5875.87 |
| Temperature/K | 90.15 | 90.00 |
| Crystal system | monoclinic | monoclinic |
| Space group | $P2_1/n$ | $P2_1/n$ |
| a/Å | 21.470(3) | 21.4359(6) |
| b/Å | 29.041(5) | 28.9930(8) |
| c/Å | 29.913(5) | 29.7998(8) |
| α/° | 90 | 90 |
| β/° | 102.403(4) | 102.6710(10) |
| γ/° | 90 | 90 |
| Volume/Å$^3$ | 18216(5) | 18069.3(9) |
| Z | 4 | 4 |
| $\rho_{calc}$g/cm$^3$ | 2.207 | 2.160 |
| μ/mm$^{-1}$ | 5.607 | 4.305 |
| F(000) | 11448.0 | 11192.0 |
| Crystal size/mm$^3$ | 0.2 × 0.001 × 0.001 | 0.5 × 0.001 × 0.001 |
| Radiation | MoKα (λ = 0.71073) | MoKα (λ = 0.71073) |
| 2θ range for data collection/° | 3.884 to 37.706 | 3.894 to 37.802 |
| Index ranges | $-19 \leq h \leq 19$, $-25 \leq k \leq 26$, $-27 \leq l \leq 27$ | $-19 \leq h \leq 19$, $-26 \leq k \leq 24$, $-27 \leq l \leq 27$ |
| Reflections collected | 52781 | 73984 |
| Independent reflections | 14202 [$R_{int}$ = 0.1563, $R_{sigma}$ = 0.1243] | 14280 [$R_{int}$ = 0.0802, $R_{sigma}$ = 0.0507] |
| Data/restraints/parameters | 14202/1698/1737 | 14280/117/1951 |
| Goodness-of-fit on F$^2$ | 1.030 | 1.029 |
| Final R indexes [I>=2σ (I)] | $R_1$ = 0.0600, $wR_2$ = 0.1235 | $R_1$ = 0.0351, $wR_2$ = 0.0755 |
| Final R indexes [all data] | $R_1$ = 0.1243, $wR_2$ = 0.1549 | $R_1$ = 0.0573, $wR_2$ = 0.0843 |
| Largest diff. peak/hole / e Å$^{-3}$ | 1.70/−1.64 | 1.30/−0.94 |

**Table S2 Assignments of the Main Transitions in 1’−4’ and the Corresponding Peaks in 1−4**

| Sample numbers | Excitation Energy (eV) | Excitation Energy (nm) | Transition [a] | Corresponding peak (nm) in 1−4 [b] | |
|---|---|---|---|---|---|
| **1’** | 1.833 | 676.50 | HOMO →LUMO+2 | **1** | 559 |
| | 2.284 | 542.92 | HOMO →LUMO+4 | | 451 |
| **2’** | 1.714 | 723.20 | HOMO →LUMO+1 | **2** | 630 |
| | 2.211 | 560.80 | HOMO →LUMO+20 | | 487 |
| **3’** | 1.841 | 673.63 | HOMO →LUMO+4 | **3** | 567 |
| | 2.201 | 563.44 | HOMO → LUMO+22 | | |
| | 2.225 | 557.20 | HOMO → LUMO+23 | | 459 |
| | 2.234 | 555.11 | HOMO → LUMO+22 | | |
| **4’** | 1.712 | 724.07 | HOMO → LUMO+3, 4 | **4** | 637 |
| | 2.086 | 594.28 | HOMO → LUMO+20 | | |
| | 2.134 | 581.01 | HOMO → LUMO+20 | | 496 |
| | 2.187 | 566.91 | HOMO → LUMO+22 | | |

[a] HOMO and LUMO indicates the highest occupied molecular orbital and the lowest unoccupied molecular orbital, respectively. [b] See Fig. 4a–d.

**Table S3 HOMO−LUMO Gaps Calculated for 1’−4’**

| Sample numbers | HOMO−LUMO gap (eV) |
|---|---|
| **1’** | +1.659 |
| **2’** | +1.549 |
| **3’** | +1.657 |
| **4’** | +1.520 |

## S4 Additional Figures

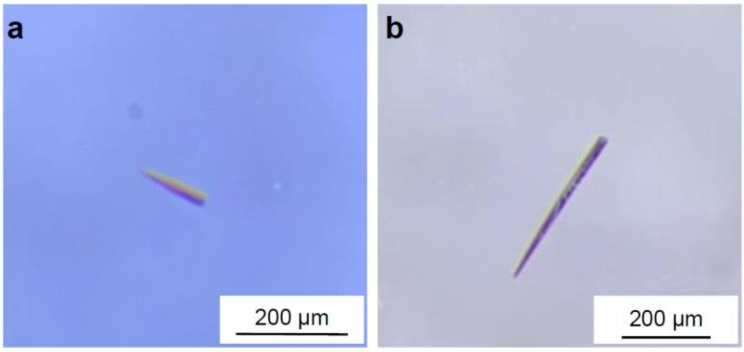


**Fig. S1: Photographs of a crystal. a 3. b 4**.

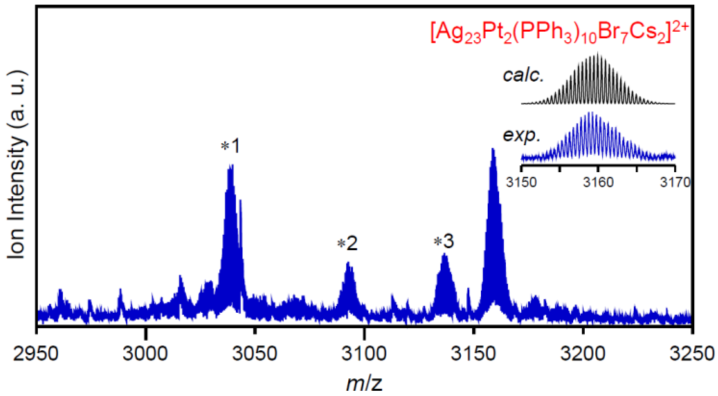


**Fig. S2: Expanded ESI-MS spectra of 3**. The inset shows the comparison between experimental and calculated mass distribution for $[Ag_{23}Pt_2(PPh_3)_{10}Br_7Cs_2]^{2+}$. The peaks *1, *2 and *3 are assigned to $[Ag_{23}Pt_2(PPh_3)_9(PPh)Br_5Cl_2Cs_2]^{2+}$, $[Ag_{23}Pt_2(PPh_3)_{10}Br_4Cl_3Cs_2]^{2+}$ and $[Ag_{23}Pt_2(PPh_3)_{10}Br_6ClCs_2]^{2+}$, respectively. Since Cl was not observed in SC-XRD analysis on **3** (Fig.1c), peaks *1, *2 and *3 seem to be produced by the contamination of Cl ions during ESI-MS. In this mass spectrum, a peak progression was observed due to the addition of $Cs_2CO_3$ as a cation source, similar to the case of the literature.[4]

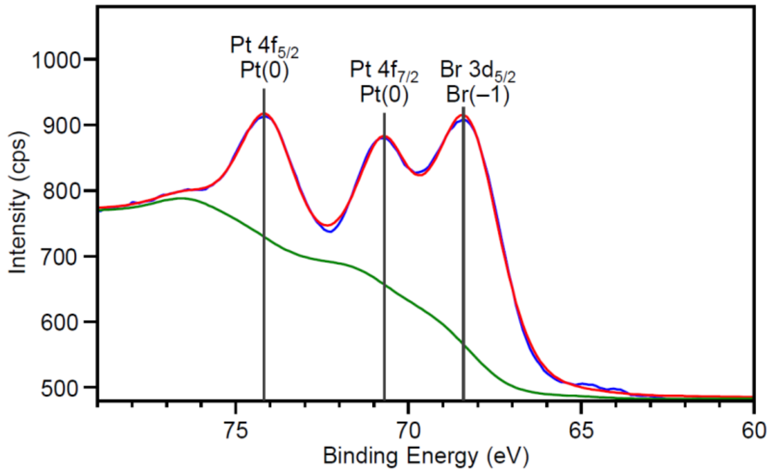


**Fig. S3: Pt 4f spectra of 3.** This spectrum indicate that Pt atom is certainly included in **3**.

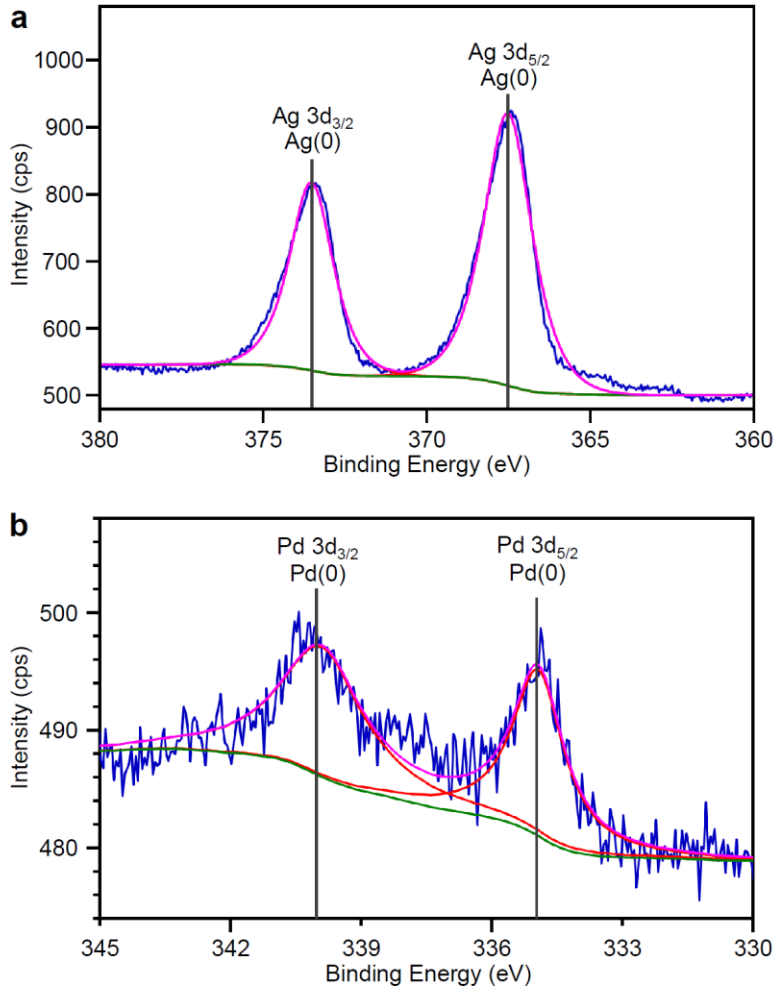


**Fig. S4 X-ray photoelectron spectra of 4. a** Ag 3d spectrum. **b** Pd 3d spectrum. The ratio of Ag:Pd in **4** was estimated from the area of each peak to be 23:1.5, being similar ratio to the expected one for **4** (23:2).

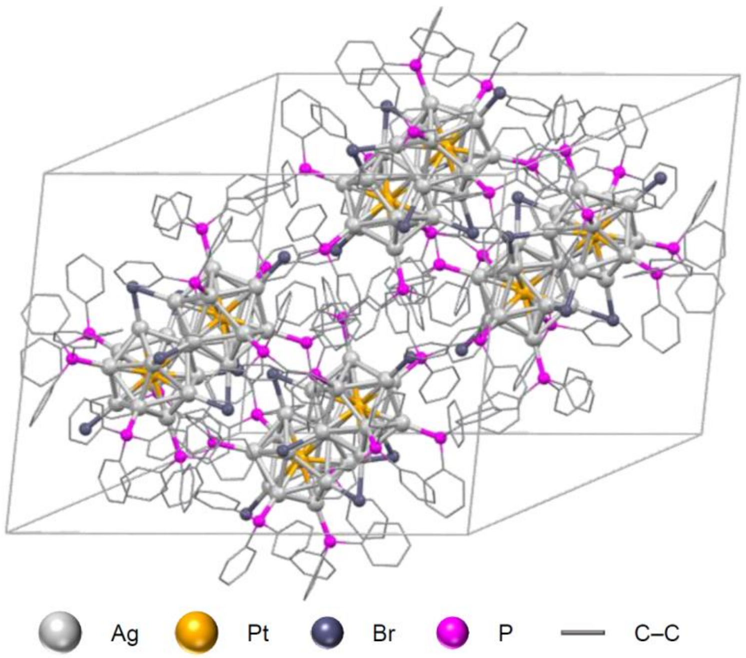


**Fig. S5: Unit cell content in the crystal of 3.** Hydrogen atoms are omitted for clarity.

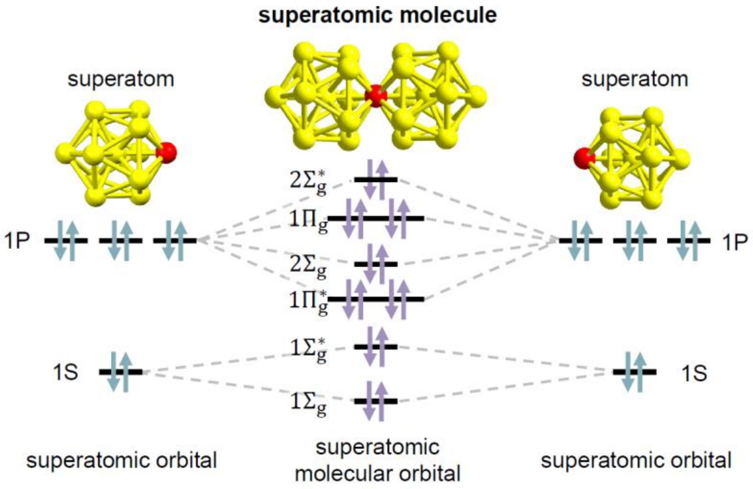


**Fig. S6: Schematic of the energy diagrams for $Au_{13}$ superatom and $Au_{25}$ superatomic molecule.**[5]

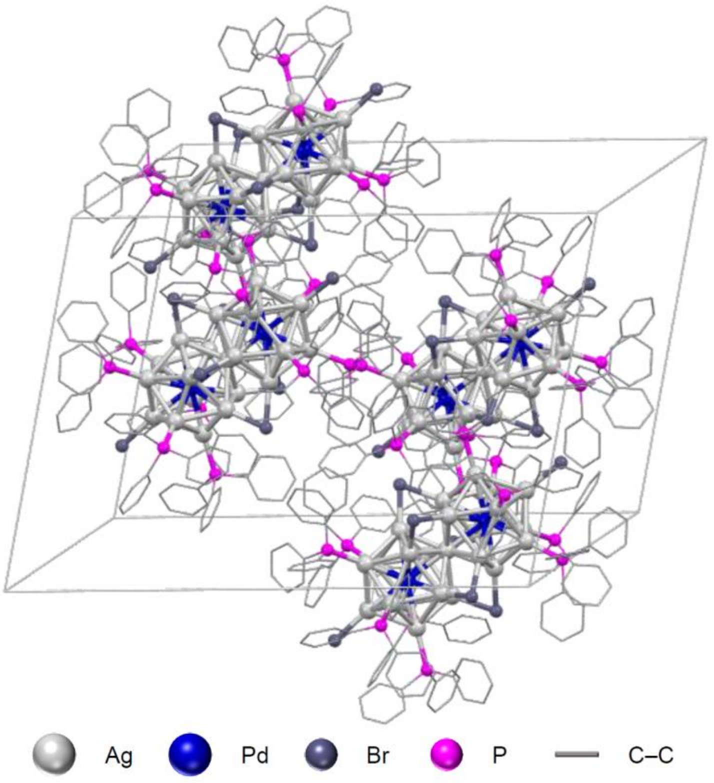


**Fig. S7: Unit cell content in the crystal of 4.** Hydrogen atoms are omitted for clarity.

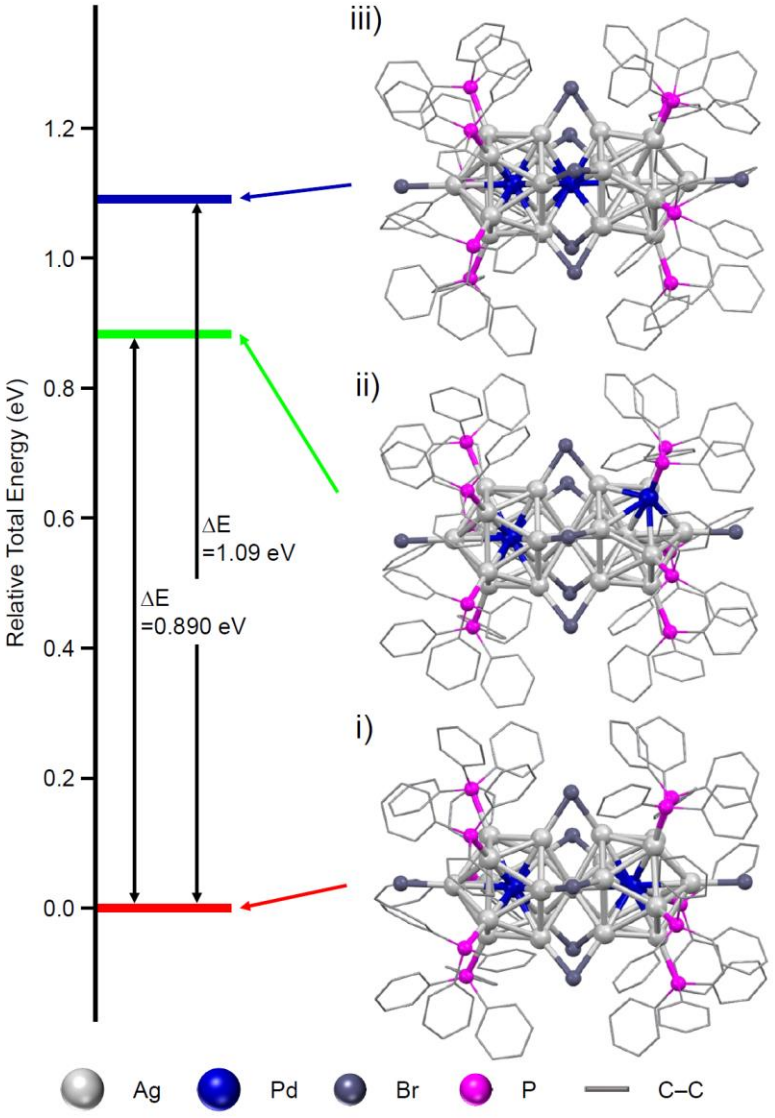


**Fig. S8: Calculated Relative energies of three types of structures for $[Ag_{23}Pd_2(PPh_3)_{10}Br_7]^0$ using PBE as a functional.** Hydrogen atoms are omitted for clarity. i) $[Ag_{23}Pd_2(PPh_3)_{10}Br_7]^0$ with the two Pd atoms at the central positions of the two $Ag_{12}Pd$ icosahedra. ii) One Pd atom at the central position of one $Ag_{12}Pd$ icosahedron and the other Pd atom on the surface of the other $Ag_{12}Pd$ icosahedron. iii) One Pd atom at the central position of one $Ag_{12}Pd$ icosahedron and the other Pd atom at the metal position connecting the two $Ag_{12}Pd$ icosahedra. In this figure, the calculated energy of i) is set to zero.

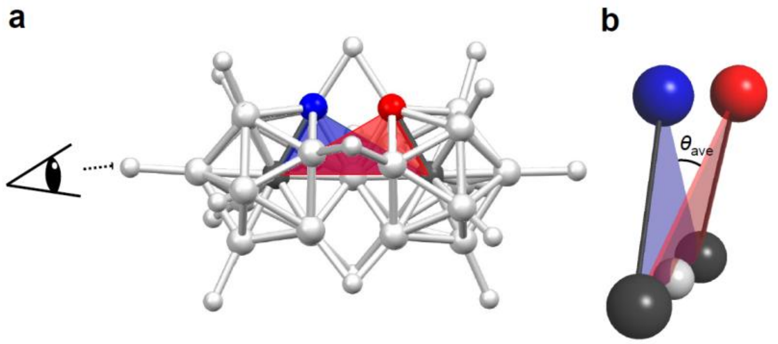


**Fig. S9: Angle showing a twist between two $Au_{12}M$ (M = Pt or Pd) core (Fig. 2a−d). a** Side view. **b** View from the long-axis direction.

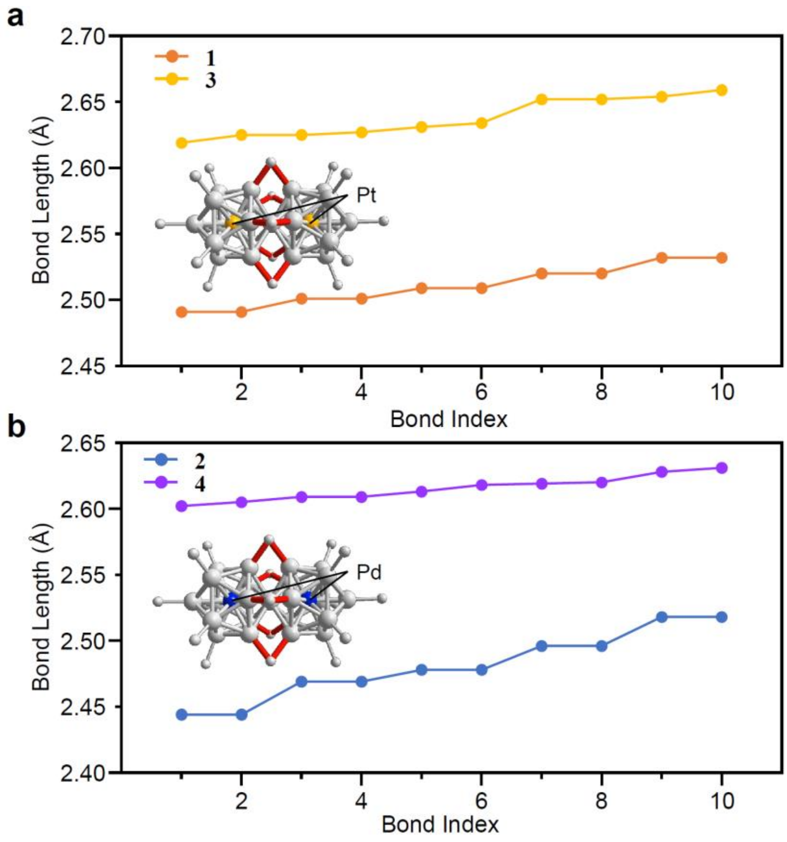


**Fig. S10: Comparison of bond lengths of Ag−X (X = Cl or Br) bonds. a** Comparison between **1** and **3**. **b** Comparison between **2** and **4**. These results demonstrate that Ag−Cl bonds are longer than Ag−Br bonds. This phenomenon is well consistent with that obtained for the normal salt (2.775 Å for Ag−Cl bond and 2.887 Å for Ag−Br bond).[6]

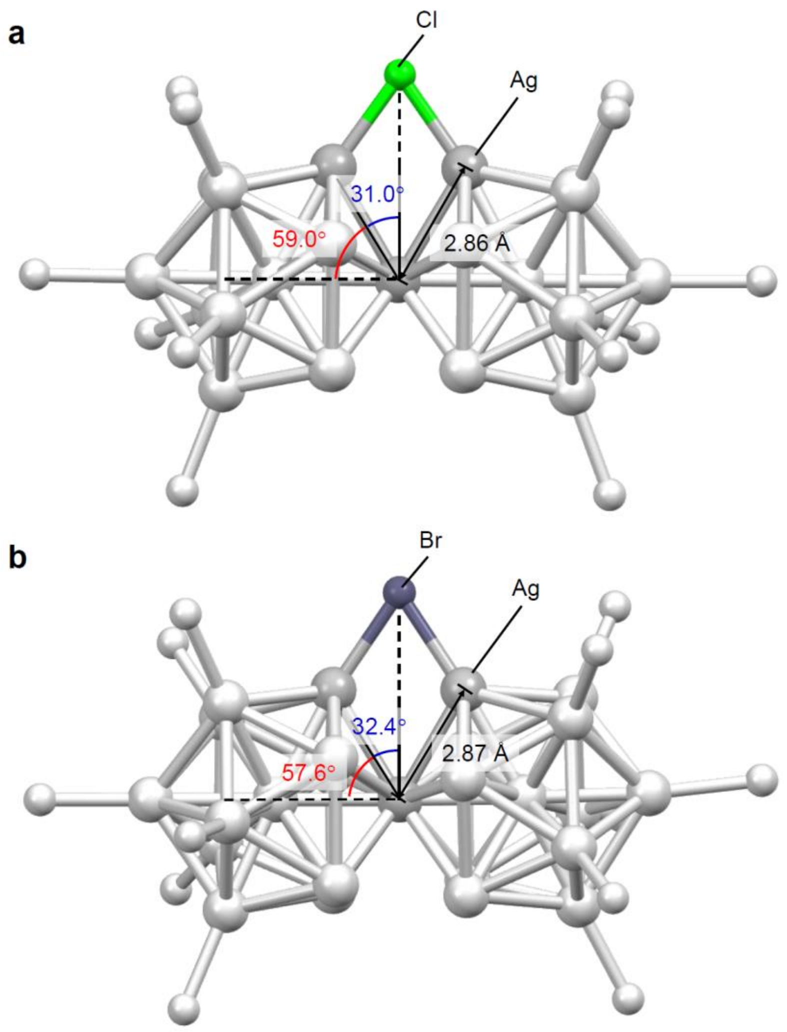


**Fig. S11: Comparison of the geometrical structure. a** Typical Ag−Ag bond length and Ag−Ag···Cl angle at the connected part in **1**. **b** Schematic of Ag−Ag bond length and Ag−Ag···Br angle estimated for the case that a twist does not occur in **3**. The elongation of Ag−Ag bond and the increase in Ag−Ag···X angle (X = halogen) will be induced in **b**. These transformations seem to destabilize each icosahedral $Ag_{12}M$ core.

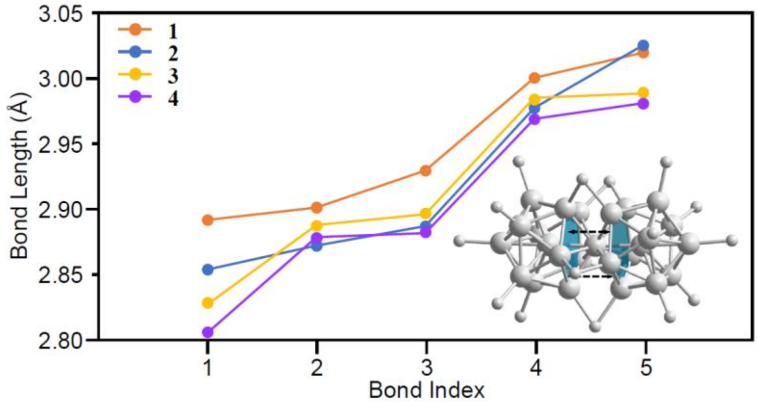


**Fig. S12: Comparison of distances between two $Ag_5$-planes in 1−4.** Since there is a twist between two $Ag_{12}M$ in **3** and **4**, these distances in **3** and **4** become similar to those in **1** and **2**, leading to the similar bond lengths between the jointing Ag and the neighbour Ag in each $Ag_{12}M$ in (see Fig. 2e). These results demonstrate that the deformation of each $Ag_{12}M$ in **3** and **4** are well avoided by the twist between two $Ag_{12}M$.

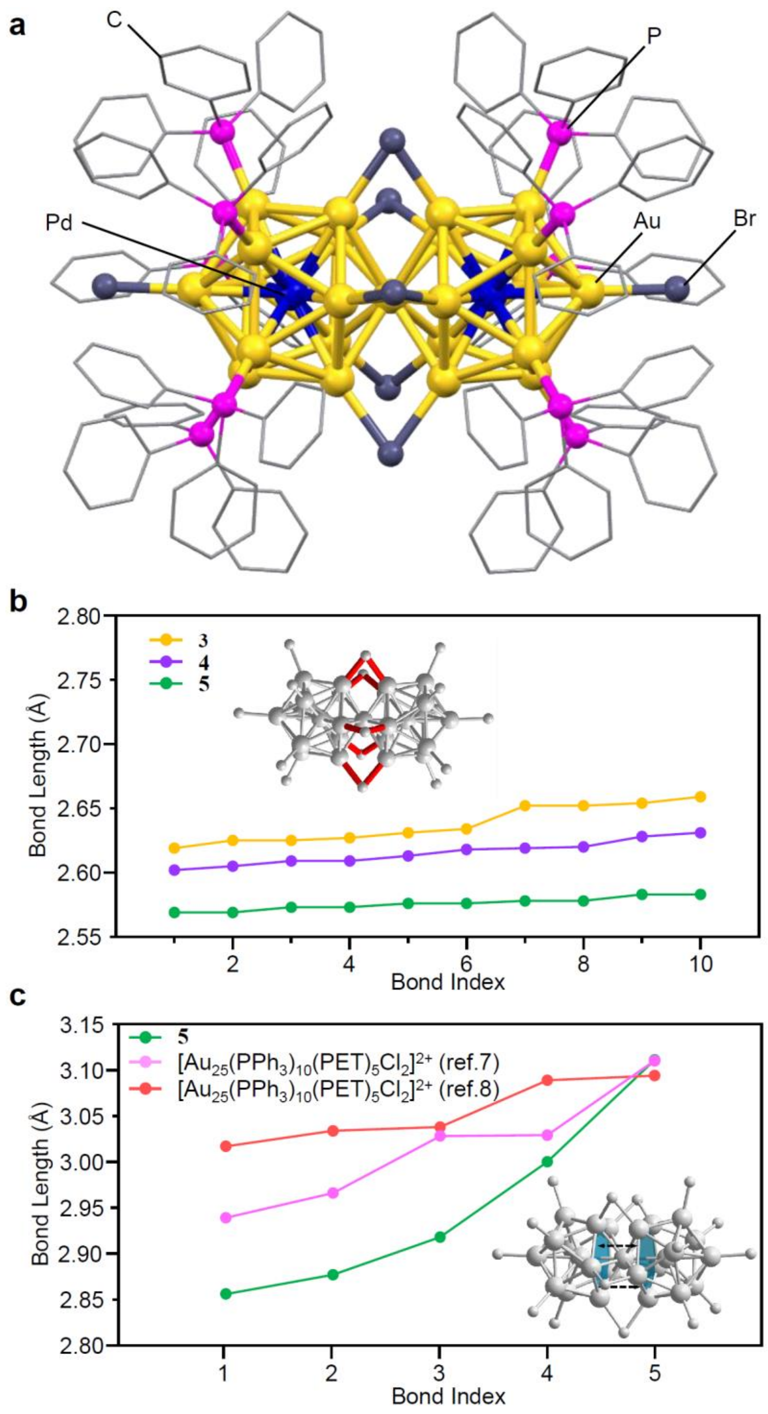


**Fig. S13: Au-based bi-superatomic molecule. a** Total structure of **5**. Hydrogen atoms are omitted for clarity. In this structure, there isn't a twist between $Au_{12}Pd$, different from the case of **3** and **4**. **b** Comparison of bond lengths of M−Br (M = Au or Ag) bonds (red line) between **3−5**, demonstrating that Au−Br bonds are shorter than Ag−Br bonds. **c** Comparison of distances between the surface Au atoms of icosahedral $Ag_{12}M$ (M = Au or Pd) between **5** and $[Au_{25}(PPh_3)_{10}(SR)_5Cl_2]^{2+}$, which is stable Au-based bi-superatomic molecule.[7,8] This figure demonstrates that distances between the surface Au atoms in **5** are shorter than those in $[Au_{25}(PPh_3)_{10}(SR)_5Cl_2]^{2+}$, implying that a twist is not required in **5**.

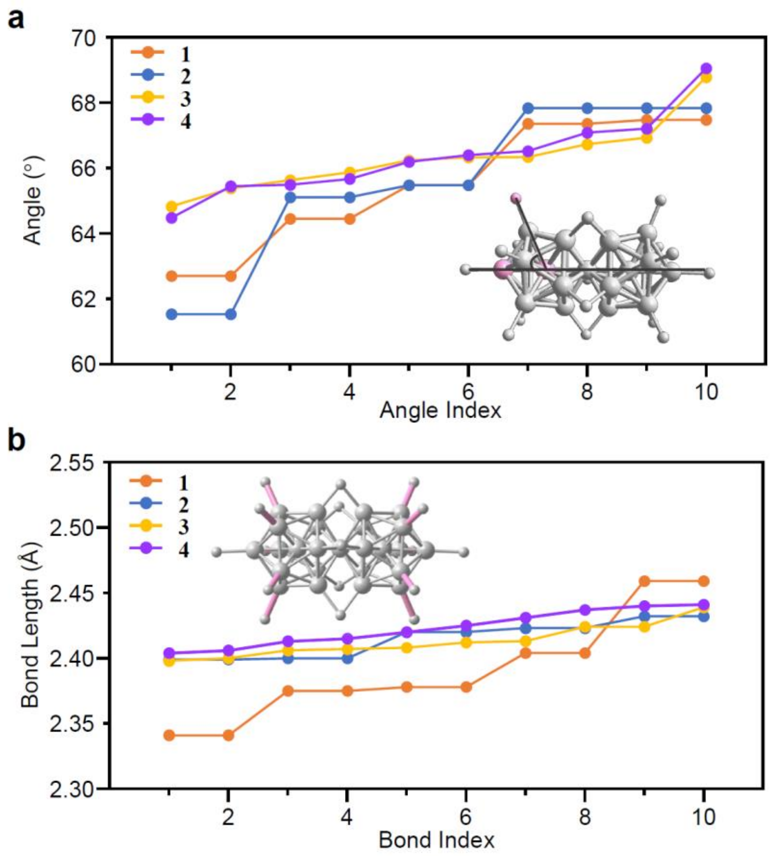


**Fig. S14: Structural analysis of 1−4. a** Comparison of the angle between the central long axis and Ag−P lines ($\theta$). **b** Comparison of Ag−P bonds (magenta line) on the surface of the $Ag_{12}M$ (M = Pt or Pd) in **1**−**4**. In **3** and **4**, the variation in the angle and bond length are small compared to **1** and **2** probably due to the steric repulsion between terminal Br and $PPh_3$. The extension of the average distance between the central long axis and P atoms also seems to occur due to the same reason (see Fig. 3).

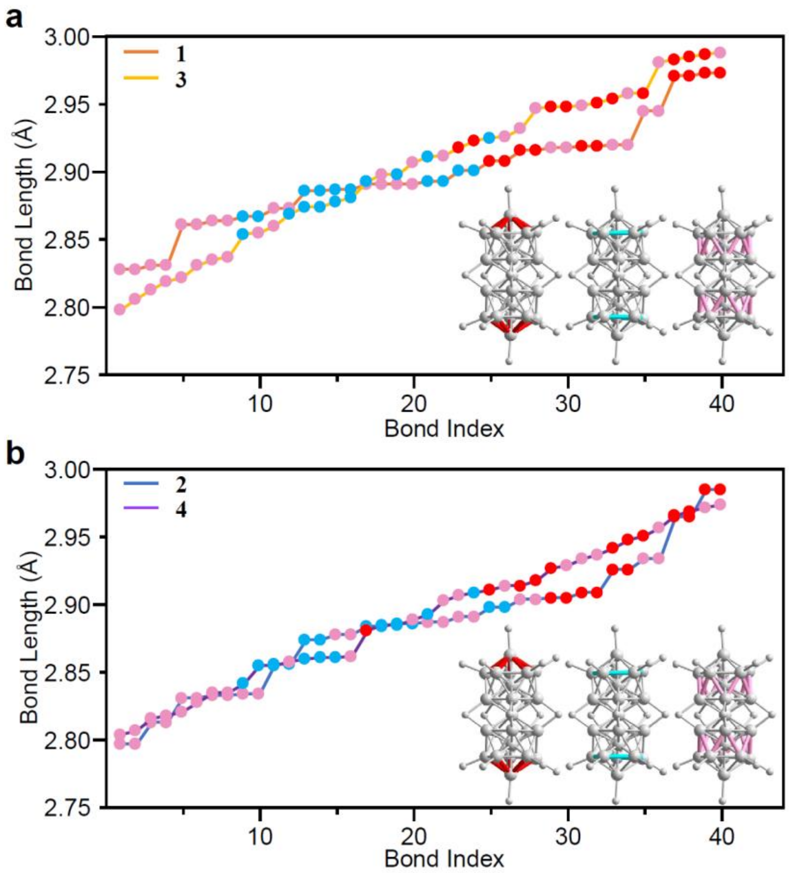


**Fig. S15: Comparison of the Ag−Ag bond lengths. a** $[Ag_{23}Pt_2(PPh_3)_{10}Br_7]^0$ (**3**) and previously reported $[Ag_{23}Pt_2(PPh_3)_{10}Cl_7]^0$ (**1**). **b** $[Ag_{23}Pd_2(PPh_3)_{10}Br_7]^0$ (**4**) and previously reported $[Ag_{23}Pd_2(PPh_3)_{10}Cl_7]^0$ (**2**). The color code of the bond position is shown in the figure.

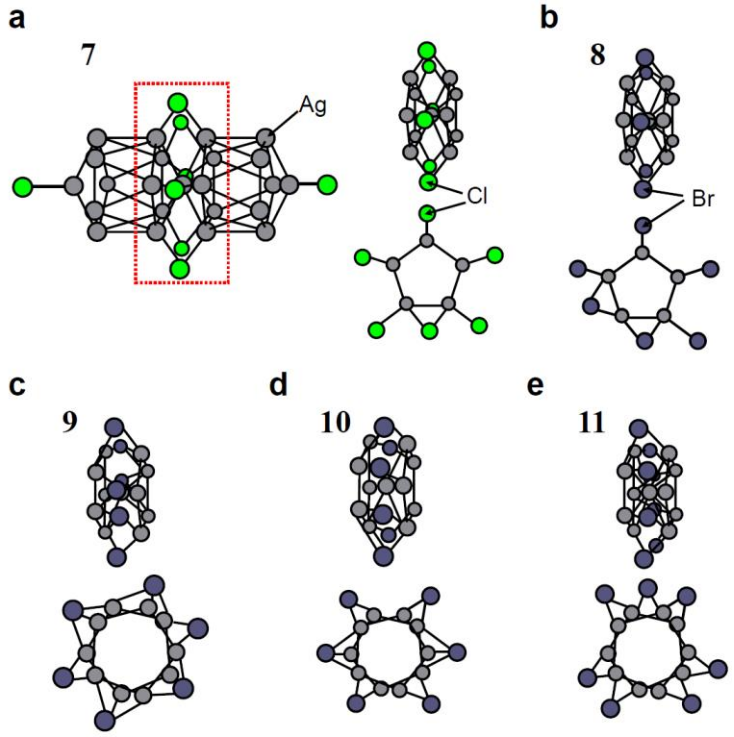


**Fig. S16: Geometry of the connecting part in 7−11. a** (left) framework structure, (upper) side view and (lower) top view of **7**. **b−e**, (upper) side view and (lower) top view for **8−11**, respectively. Reproduced from ref. 9.

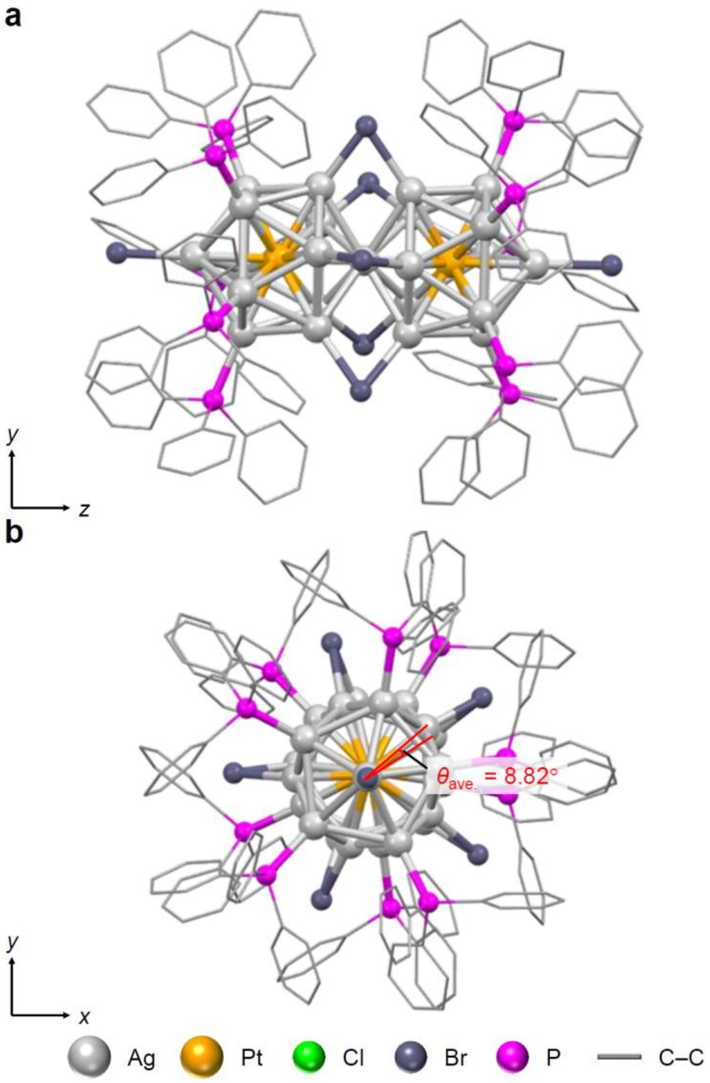


**Fig. S17: Calculated geometrical structure of 3'. a** Side view. **b** View from long-axis direction. Hydrogen atoms are omitted for clarity.

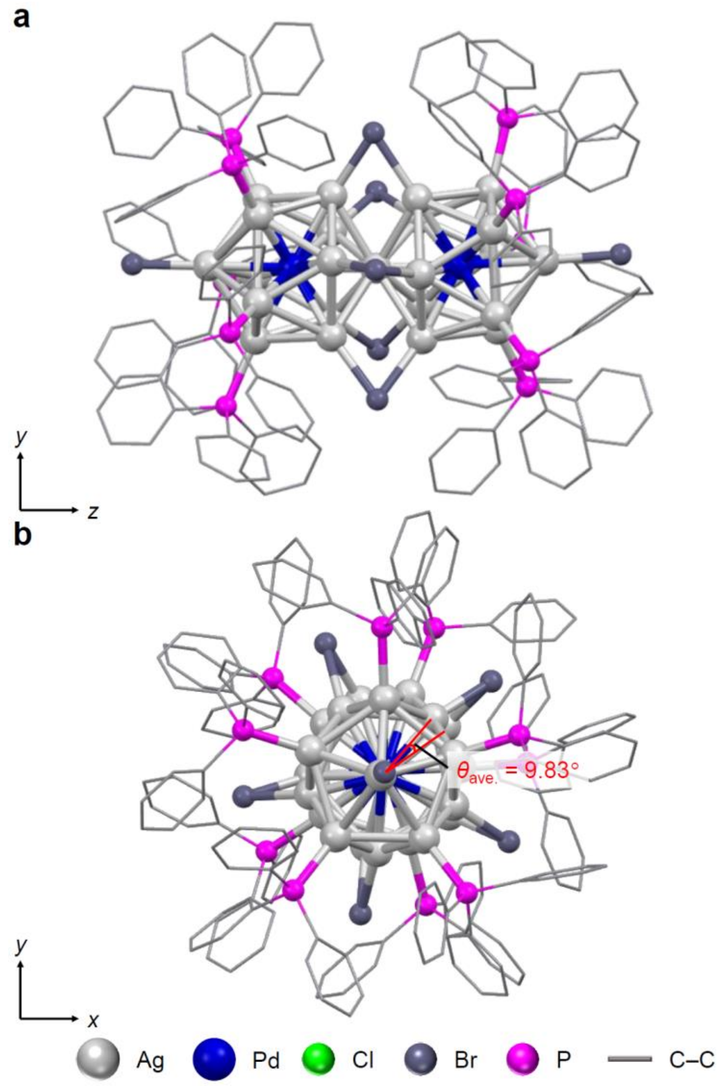


**Fig. S18: Calculated geometrical structure of 4’. a** Side view. **b** View from long-axis direction. Hydrogen atoms are omitted for clarity.

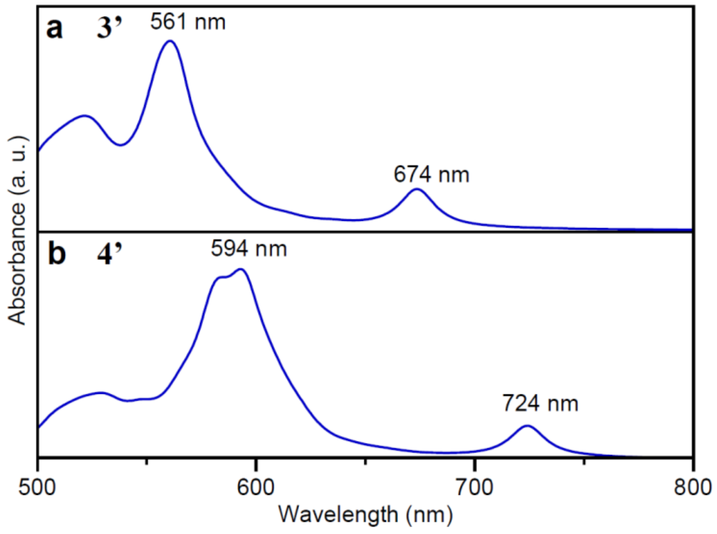


**Fig. S19: Calculated absorption spectrum. a 3’. b 4’**.

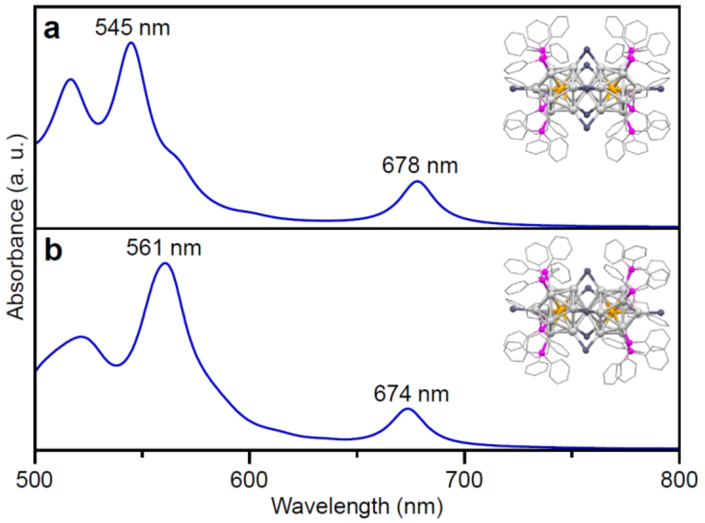


**Fig. S20: Comparison of the calculated optical absorption spectrum between a $[Ag_{23}Pt_2(PPh_3)_{10}Br_7]^0$ without distortion and b $[Ag_{23}Pt_2(PPh_3)_{10}Br_7]^0$ with distortion (3').** In the calculation of $[Ag_{23}Pt_2(PPh_3)_{10}Br_7]^0$ without distortion, Cl of 1' was replaced with Br.

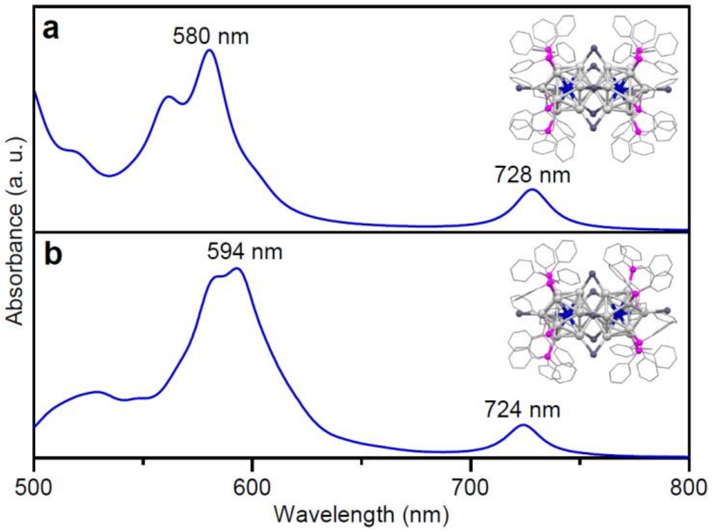


**Fig. S21: Comparison of the calculated optical absorption spectrum between a $[Ag_{23}Pd_2(PPh_3)_{10}Br_7]^0$ without distortion and b $[Ag_{23}Pd_2(PPh_3)_{10}Br_7]^0$ with distortion (4').** In the calculation of $[Ag_{23}Pd_2(PPh_3)_{10}Br_7]^0$ without distortion, Cl of 2' was replaced with Br.

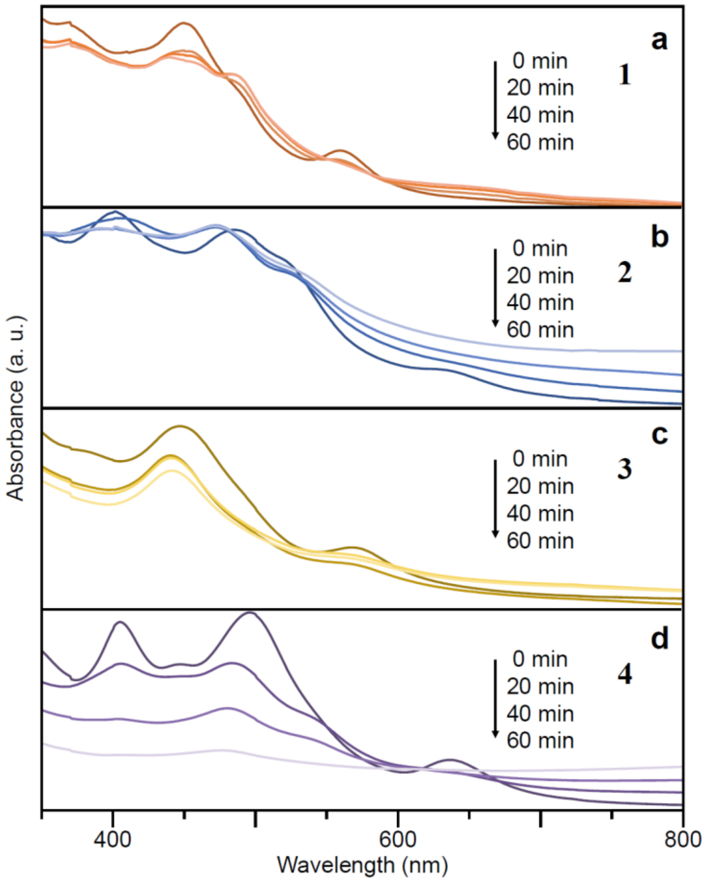


**Fig. 22: Time dependence of the optical absorption spectra of cluster dichloromethane solution. a−d 1**, **2**, **3**, and **4**, respectively.

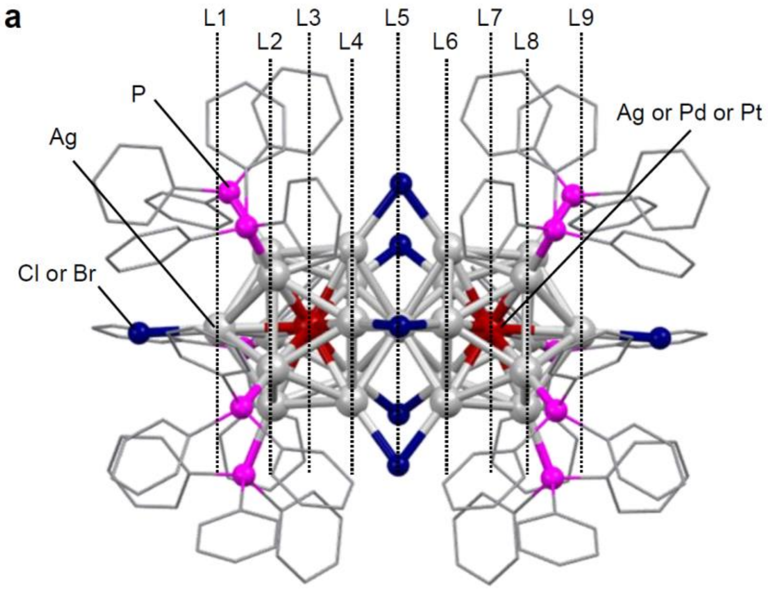


**b**

| | **1’** | **2’** | **5’** |
|---|---|---|---|
| L1 | 0.23 | 0.22 | 0.25 |
| L2 | 0.27 | 0.26 | 0.27 |
| L3 | −1.24 | −1.05 | −0.59 |
| L4 | 0.29 | 0.27 | 0.28 |
| L5 | −0.12 | −0.14 | −0.20 |
| L6 | 0.29 | 0.27 | 0.28 |
| L7 | −1.24 | −1.05 | −0.59 |
| L8 | 0.27 | 0.26 | 0.27 |
| L9 | 0.23 | 0.22 | 0.25 |

| | **3’** | **4’** | **6’** |
|---|---|---|---|
| L1 | 0.24 | 0.22 | 0.25 |
| L2 | 0.28 | 0.27 | 0.27 |
| L3 | −1.22 | −1.03 | −0.56 |
| L4 | 0.27 | 0.26 | 0.26 |
| L5 | −0.12 | −0.14 | −0.21 |
| L6 | 0.27 | 0.26 | 0.26 |
| L7 | −1.22 | −1.03 | −0.56 |
| L8 | 0.28 | 0.27 | 0.27 |
| L9 | 0.24 | 0.23 | 0.25 |

**Fig. S23: Natural charge analysis on the calculated structures 1’−6’. a** Schematic showing the layer index (L1–L9). **b** Average natural charge in each layer of **1’−6’**. Hydrogen atoms are omitted for clarity.

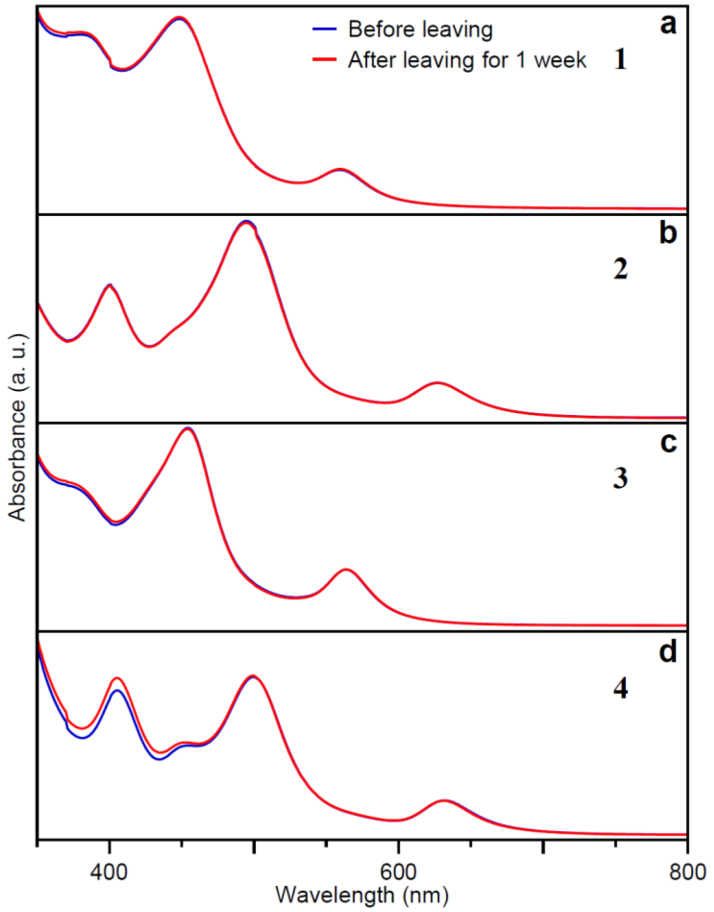


**Fig. S24: Optical absorption spectra of cluster toluene solution prepared by dissolving crystals of 1−4 before and after leaving for 1 week. a 1**, **b 2**, **c 3** and **d 4**. In these experiments, an excess amount of $PPh_3$ (95 mM) was dissolved to suppress the degradation of **1**–**4** in solution. These spectra demonstrate that the stability of **1**–**4** in solution is largely improved by the addition of an excess amount of $PPh_3$ to the solution.

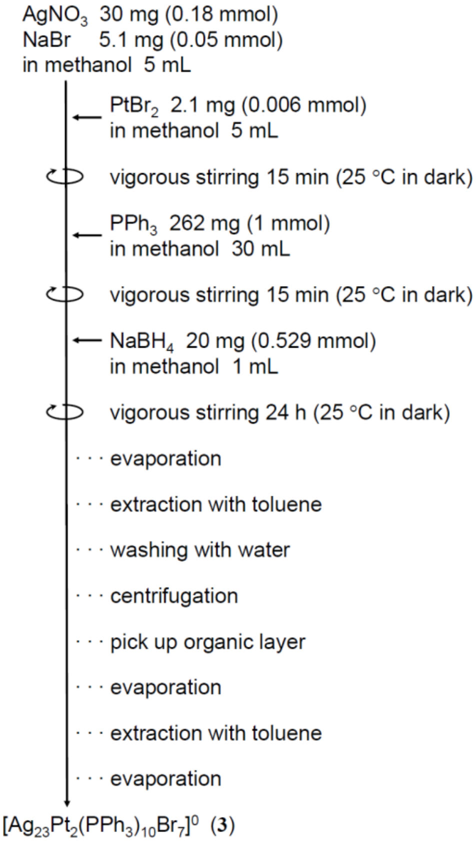


**Fig. S25: Protocol of the synthesis of 3.**

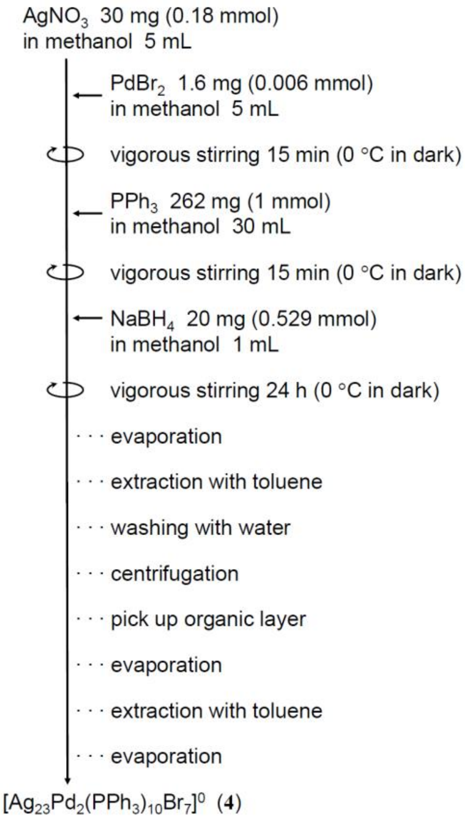


**Fig. S26: Protocol of the synthesis of 4.**

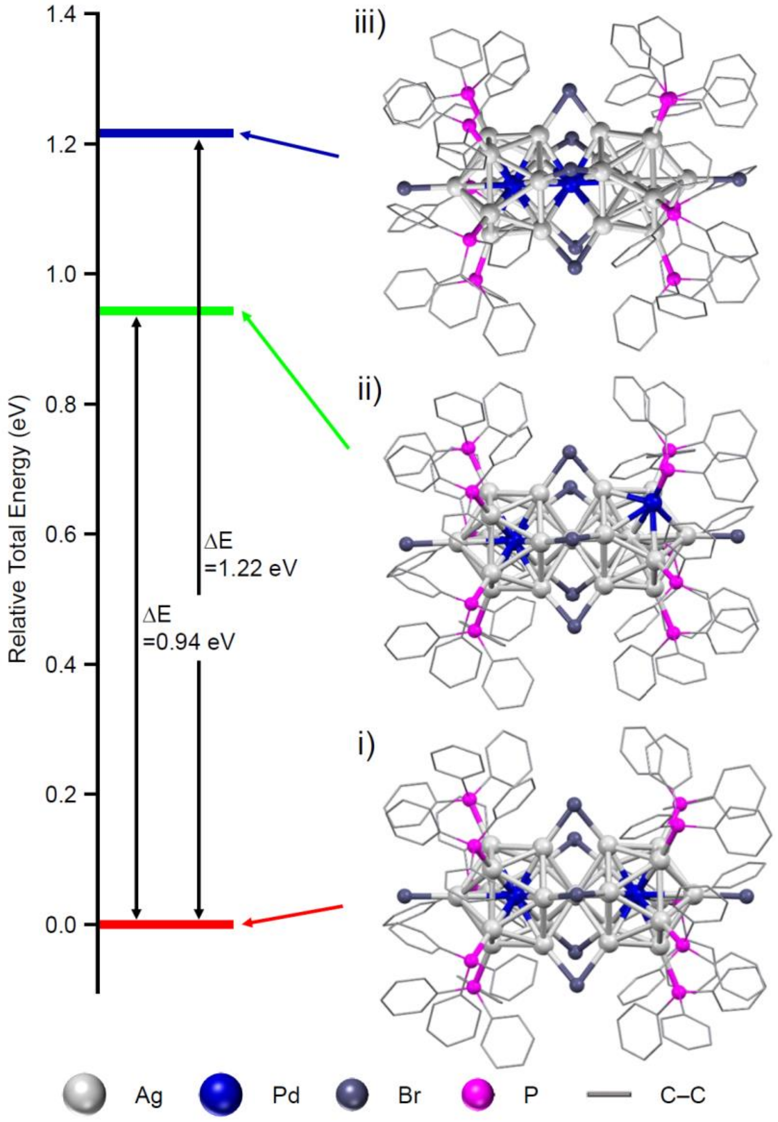


**Fig. S27: Calculated Relative energies of three types of structures for $[Ag_{23}Pd_2(PPh_3)_{10}Br_7]^0$ using CAM-B3LYP instead of PBE as a functional.** Hydrogen atoms are omitted for clarity. i) $[Ag_{23}Pd_2(PPh_3)_{10}Br_7]^0$ with the two Pd atoms at the central positions of the two $Ag_{12}Pd$ icosahedra. ii) One Pd atom at the central position of one $Ag_{12}Pd$ icosahedron and the other Pd atom on the surface of the other $Ag_{12}Pd$ icosahedron. iii) One Pd atom at the central position of one $Ag_{12}Pd$ icosahedron and the other Pd atom at the metal position connecting the two $Ag_{12}Pd$ icosahedra. In this figure, the calculated energy of i) is set to zero.

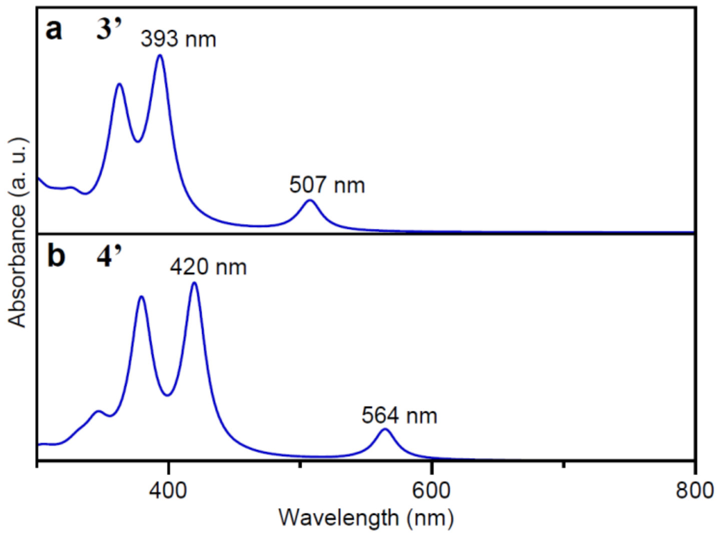


**Fig. S28: Calculated absorption spectrum using CAM-B3LYP instead of PBE as a functional. a 3'**. **b 4'**.

## S5. Supplementary References


1. Bruker APEX3, v2019.1–0, Bruker AXS Inc., Madison, WI, USA, (2019).
2. Sheldrick, G. M. Crystal structure refinement with SHELXL. *Acta Crystallogr., Sect. C: Struct. Chem.* **71**, 3–8 (2015).
3. Dolomanov, O. V., Bourhis, L. J., Gildea, R. J., Howard, J. A. K. & Puschmann, H. OLEX2: a complete structure solution, refinement and analysis program. *J. Appl. Crystallogr.* **42**, 339–341 (2009).
4. Bootharaju, M. S. *et al.* Doping-induced anisotropic self-assembly of silver icosahedra in $[Pt_2Ag_{23}Cl_7(PPh_3)_{10}]$ nanoclusters. *J. Am. Chem. Soc.* **139**, 1053–1056 (2017).
5. Mingos, D. M. P. Structural and bonding patterns in gold clusters. *Dalton Trans.* **44**, 6680-6695 (2015).
6. Haynes, W. M. *Handbook of Chemistry and Physics* (ed. Haynes, W. M.) (CRC Press, New York, 2007).
7. Shichibu, Y. *et al.* Biicosahedral gold clusters $[Au_{25}(PPh_3)_{10}(SC_nH_{2n+1})_5Cl_2]^{2+}$ ($n = 2-18$): a stepping stone to cluster-assembled materials. *J. Phys. Chem. C* **111**, 7845–7847 (2007).
8. Qian, H., Eckenhoff, W. T., Bier, M. E., Pintauer, T. & Jin, R. Crystal structures of $Au_2$ complex and $Au_{25}$ nanocluster and mechanistic insight into the conversion of polydisperse nanoparticles into monodisperse $Au_{25}$ nanoclusters. *Inorg. Chem.* **50**, 10735–10739 (2011).
9. Teo, B. K. & Zhang, H. Polyicosahedricity: icosahedron to icosahedron of icosahedra growth pathway for bimetallic (Au–Ag) and trimetallic (Au–Ag–M; M = Pt, Pd, Ni) supraclusters; synthetic strategies, site preference, and stereochemical principles. *Coord. Chem. Rev.* **143**, 611–636 (1995).
