# Supplementary material for: Key factors for connecting silver-based icosahedral superatoms by vertex sharing
Source: Commun Chem. 2023 Mar 28;6:57. doi: 10.1038/s42004-023-00854-0 (PMC10050180; doi:10.1038/s42004-023-00854-0)
Supplement: Supplementary file 3 — Description of Additional Supplementary Files [file 42004_2023_854_MOESM3_ESM.pdf]

Description of Additional Supplementary Files

File Name: Supplementary Data 1

Description: crystallographic information file (cif) of **3**.

File Name: Supplementary Data 2

Description: cif check of **3**.

File Name: Supplementary Data 3

Description: crystallographic information file (cif) of **4**.

File Name: Supplementary Data 4

Description: cif check of **4**.

File Name: Supplementary Data 5

Description: atomic coordinates of **1′**.

File Name: Supplementary Data 6

Description: atomic coordinates of **2′**.

File Name: Supplementary Data 7

Description: atomic coordinates of **3′**.

File Name: Supplementary Data 8

Description: atomic coordinates of **4′**.

File Name: Supplementary Data 9

Description: atomic coordinates of **6′**.
