# Supplementary material for: Key factors for connecting silver-based icosahedral superatoms by vertex sharing
Source: Commun Chem. 2023 Mar 28;6:57. doi: 10.1038/s42004-023-00854-0 (PMC10050180; doi:10.1038/s42004-023-00854-0)

checkCIF/PLATON report

You have not supplied any structure factors. As a result the full set of tests cannot be run.

THIS REPORT IS FOR GUIDANCE ONLY. IF USED AS PART OF A REVIEW PROCEDURE FOR PUBLICATION, IT SHOULD NOT REPLACE THE EXPERTISE OF AN EXPERIENCED CRYSTALLOGRAPHIC REFEREE.

No syntax errors found. CIF dictionary Interpreting this report

Datablock: 3

```
Bond precision:   C-C = 0.0516 A          Wavelength=0.71073

Cell:              a=21.470(3)     b=29.041(5)          c=29.913(5)
                   alpha=90        beta=102.403(4)      gamma=90
Temperature:       90 K

                 Calculated                         Reported
Volume           18216(5)                           18216(5)
Space group      P 21/n                             P 1 21/n 1
Hall group       -P 2yn                             -P 2yn
Moiety formula   C180 H150 Ag23 Br7 P10 Pt2  C180 H150 Ag23 Br7 P10 Pt2
Sum formula      C180 H150 Ag23 Br7 P10 Pt2  C180 H150 Ag23 Br7 P10 Pt2
Mr               6053.11                            6053.25
Dx,g cm-3        2.207                              2.207
Z                4                                  4
Mu (mm-1)        5.607                              5.607
F000             11448.0                            11448.0
F000'            11351.51
h,k,lmax         19,26,27                           19,26,27
Nref             14341                              14202
Tmin,Tmax        0.993,0.994                        0.637,0.744
Tmin'            0.326

Correction method= # Reported T Limits: Tmin=0.637 Tmax=0.744
AbsCorr = MULTI-SCAN

Data completeness= 0.990            Theta(max)= 18.853

                                                    wR2(reflections)=
R(reflections)= 0.0600( 8583)                       0.1549( 14202)

S = 1.030                  Npar= 1737
```

The following ALERTS were generated. Each ALERT has the format
test-name_ALERT_alert-type_alert-level.
Click on the hyperlinks for more details of the test.

Alert level A

THETM01_ALERT_3_A The value of sine(theta_max)/wavelength is less than 0.550
Calculated sin(theta_max)/wavelength = 0.4547

Author Response: Despite several attempts over six months, resolution of cluster3's diffraction could not be improved better than 1.10 A.

PLAT201_ALERT_2_A Isotropic non-H Atoms in Main Residue(s) .... 38 Report
C1 C11 C18 C35 C37 etc.

Author Response: These carbon atoms have high thermal parameters.

Alert level B

PLAT220_ALERT_2_B NonSolvent Resd 1 C Ueq(max)/Ueq(min) Range 9.1 Ratio

Author Response: This carbon has very high thermal parameters.

PLAT241_ALERT_2_B High 'MainMol' Ueq as Compared to Neighbors of C114 Check

Author Response: This carbon has very high thermal parameters.

PLAT342_ALERT_3_B Low Bond Precision on C-C Bonds .... 0.05156 Ang.

Author Response: Phenyl rings of few PPh3 ligands have disorder.

PLAT601_ALERT_2_B Unit Cell Contains Solvent Accessible VOIDS of . 183 Ang**3

Author Response: Cluster 3 has very few low electron density q peaks (1.70, 1.190,1.130, 1.030, 1.000, 0.940, 0.900)outside main structural frame structure.These q peaks could be solvent of crystallization with fractions of occupancy

Alert level C

```
RINTA01_ALERT_3_C  The value of Rint is greater than 0.12
            Rint given   0.156
PLAT088_ALERT_3_C Poor Data / Parameter Ratio ....................       8.26 Note
PLAT213_ALERT_2_C Atom C4              has ADP max/min Ratio .....        3.5 prolat
PLAT213_ALERT_2_C Atom C77             has ADP max/min Ratio .....        3.2 prolat
PLAT213_ALERT_2_C Atom C95             has ADP max/min Ratio .....        3.4 prolat
PLAT213_ALERT_2_C Atom C119            has ADP max/min Ratio .....        3.5 oblate
PLAT220_ALERT_2_C NonSolvent   Resd 1 Br   Ueq(max)/Ueq(min) Range        3.2 Ratio
```

Author Response: This carbon has very high thermal parameters.

```
PLAT222_ALERT_3_C NonSolvent Resd 1  H   Uiso(max)/Uiso(min) Range        7.4 Ratio
PLAT234_ALERT_4_C Large Hirshfeld Difference P5       --C92      .       0.22 Ang.
PLAT241_ALERT_2_C High   'MainMol' Ueq as Compared to Neighbors of        C85 Check
```

Author Response: This carbon has very high thermal parameters.

```
PLAT241_ALERT_2_C High   'MainMol' Ueq as Compared to Neighbors of        C89 Check
```

Author Response: This carbon has very high thermal parameters.

```
PLAT241_ALERT_2_C High   'MainMol' Ueq as Compared to Neighbors of       C107 Check
```

Author Response: This carbon has very high thermal parameters.

```
PLAT241_ALERT_2_C High   'MainMol' Ueq as Compared to Neighbors of       C127 Check
```

Author Response: This carbon has very high thermal parameters.

```
PLAT241_ALERT_2_C High   'MainMol' Ueq as Compared to Neighbors of       C128 Check
```

Author Response: This carbon has very high thermal parameters.

```
PLAT241_ALERT_2_C High   'MainMol' Ueq as Compared to Neighbors of       C134 Check
```

Author Response: This carbon has very high thermal parameters.

```
PLAT241_ALERT_2_C High   'MainMol' Ueq as Compared to Neighbors of       C157 Check
```

Author Response: This carbon has very high thermal parameters.

```
PLAT241_ALERT_2_C High   'MainMol' Ueq as Compared to Neighbors of       C175 Check
```

Author Response: This carbon has very high thermal parameters.

```
PLAT241_ALERT_2_C High   'MainMol' Ueq as Compared to Neighbors of       C176 Check
```

Author Response: This carbon has very high thermal parameters.

```
PLAT241_ALERT_2_C High   'MainMol' Ueq as Compared to Neighbors of       C177 Check
```

Author Response: This carbon has very high thermal parameters.

```
PLAT241_ALERT_2_C High   'MainMol' Ueq as Compared to Neighbors of       C179 Check
```

Author Response: This carbon has very high thermal parameters.

```
PLAT242_ALERT_2_C Low    'MainMol' Ueq as Compared to Neighbors of         C1 Check
PLAT242_ALERT_2_C Low    'MainMol' Ueq as Compared to Neighbors of        C18 Check
PLAT242_ALERT_2_C Low    'MainMol' Ueq as Compared to Neighbors of        C35 Check
PLAT242_ALERT_2_C Low    'MainMol' Ueq as Compared to Neighbors of        C90 Check
PLAT242_ALERT_2_C Low    'MainMol' Ueq as Compared to Neighbors of       C110 Check
PLAT242_ALERT_2_C Low    'MainMol' Ueq as Compared to Neighbors of       C112 Check
PLAT242_ALERT_2_C Low    'MainMol' Ueq as Compared to Neighbors of       C117 Check
PLAT242_ALERT_2_C Low    'MainMol' Ueq as Compared to Neighbors of       C118 Check
PLAT242_ALERT_2_C Low    'MainMol' Ueq as Compared to Neighbors of       C126 Check
PLAT242_ALERT_2_C Low    'MainMol' Ueq as Compared to Neighbors of       C130 Check
PLAT242_ALERT_2_C Low    'MainMol' Ueq as Compared to Neighbors of       C154 Check
PLAT242_ALERT_2_C Low    'MainMol' Ueq as Compared to Neighbors of       C174 Check
PLAT331_ALERT_2_C Small Aver Phenyl C-C Dist C3       --C84      .       1.36 Ang.
PLAT332_ALERT_2_C Large Phenyl C-C Range     C7        -C102     .       0.16 Ang.
PLAT332_ALERT_2_C Large Phenyl C-C Range     C30       -C55      .       0.16 Ang.
```

Alert level G

```
PLAT003_ALERT_2_G Number of Uiso or Uij Restrained non-H Atoms ...        144 Report
PLAT020_ALERT_3_G The Value of Rint is Greater Than 0.12 .........      0.156 Report
PLAT083_ALERT_2_G SHELXL Second Parameter in WGHT  Unusually Large     398.09 Why ?
PLAT178_ALERT_4_G The CIF-Embedded .res File Contains SIMU Records          1 Report
PLAT186_ALERT_4_G The CIF-Embedded .res File Contains ISOR Records          1 Report
PLAT187_ALERT_4_G The CIF-Embedded .res File Contains RIGU Records          1 Report
PLAT232_ALERT_2_G Hirshfeld Test Diff (M-X)  Ag11     --Br3      .        9.2 s.u.
PLAT232_ALERT_2_G Hirshfeld Test Diff (M-X)  Ag21     --Br5      .        5.4 s.u.
PLAT793_ALERT_4_G Model has Chirality at P6         (Centro SPGR)           S Verify
PLAT793_ALERT_4_G Model has Chirality at P8         (Centro SPGR)           R Verify
PLAT793_ALERT_4_G Model has Chirality at P9         (Centro SPGR)           R Verify
PLAT793_ALERT_4_G Model has Chirality at P10        (Centro SPGR)           S Verify
PLAT860_ALERT_3_G Number of Least-Squares Restraints ............        1698 Note
PLAT941_ALERT_3_G Average HKL Measurement Multiplicity ..........         3.7 Low
```

---

2 **ALERT level A** = Most likely a serious problem - resolve or explain

```
 4 ALERT level B = A potentially serious problem, consider carefully
35 ALERT level C = Check. Ensure it is not caused by an omission or oversight
14 ALERT level G = General information/check it is not something unexpected

 0 ALERT type 1 CIF construction/syntax error, inconsistent or missing data
39 ALERT type 2 Indicator that the structure model may be wrong or deficient
 8 ALERT type 3 Indicator that the structure quality may be low
 8 ALERT type 4 Improvement, methodology, query or suggestion
 0 ALERT type 5 Informative message, check
```

It is advisable to attempt to resolve as many as possible of the alerts in all categories. Often the minor alerts point to easily fixed oversights, errors and omissions in your CIF or refinement strategy, so attention to these fine details can be worthwhile. In order to resolve some of the more serious problems it may be necessary to carry out additional measurements or structure refinements. However, the purpose of your study may justify the reported deviations and the more serious of these should normally be commented upon in the discussion or experimental section of a paper or in the "special_details" fields of the CIF. checkCIF was carefully designed to identify outliers and unusual parameters, but every test has its limitations and alerts that are not important in a particular case may appear. Conversely, the absence of alerts does not guarantee there are no aspects of the results needing attention. It is up to the individual to critically assess their own results and, if necessary, seek expert advice.

Publication of your CIF in IUCr journals

A basic structural check has been run on your CIF. These basic checks will be run on all CIFs submitted for publication in IUCr journals (*Acta Crystallographica*, *Journal of Applied Crystallography*, *Journal of Synchrotron Radiation*); however, if you intend to submit to *Acta Crystallographica Section C* or *E* or *IUCrData*, you should make sure that full publication checks are run on the final version of your CIF prior to submission.

Publication of your CIF in other journals

Please refer to the *Notes for Authors* of the relevant journal for any special instructions relating to CIF submission.

PLATON version of 28/11/2022; check.def file version of 28/11/2022

Datablock 3 - ellipsoid plot

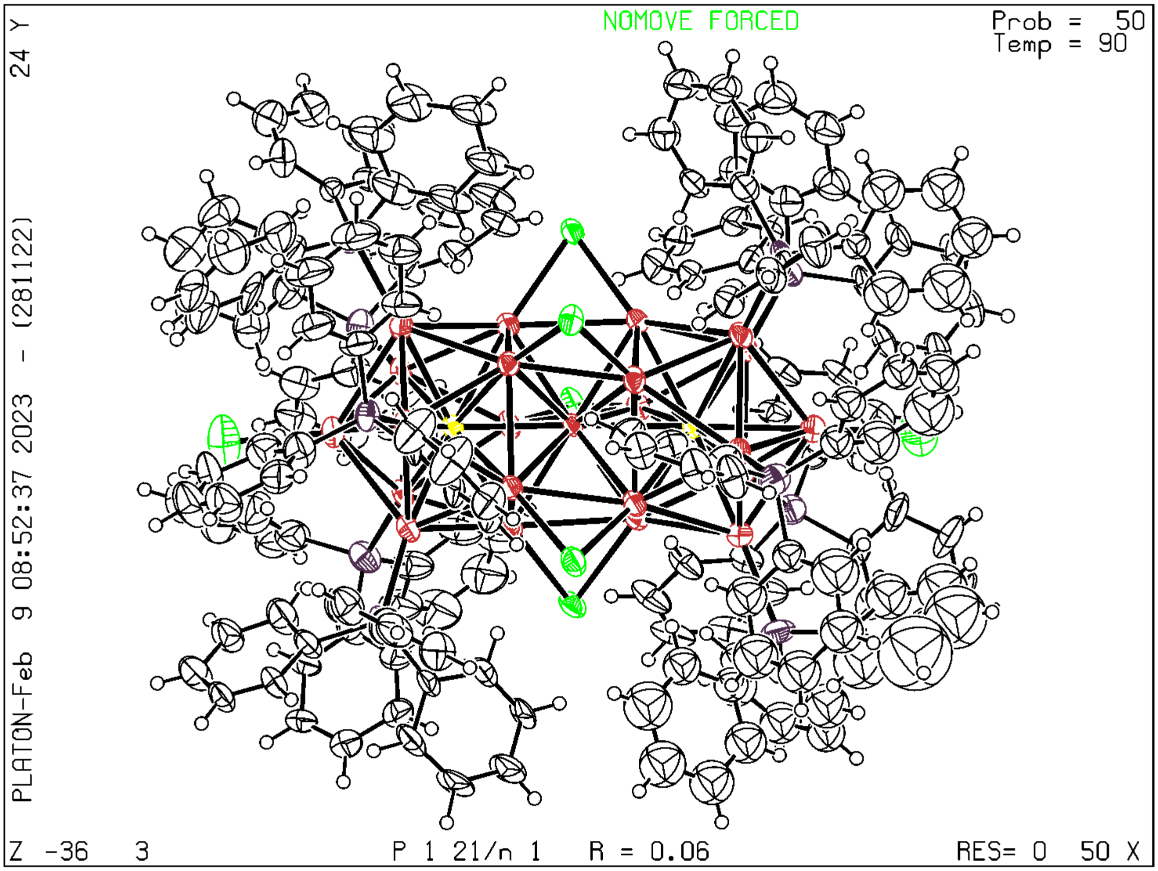

Supplement: Supplementary file 5 — Supplementary Data 2 [file 42004_2023_854_MOESM5_ESM.pdf]
