# Supplementary material for: Key factors for connecting silver-based icosahedral superatoms by vertex sharing
Source: Commun Chem. 2023 Mar 28;6:57. doi: 10.1038/s42004-023-00854-0 (PMC10050180; doi:10.1038/s42004-023-00854-0)

checkCIF/PLATON report

You have not supplied any structure factors. As a result the full set of tests cannot be run.

THIS REPORT IS FOR GUIDANCE ONLY. IF USED AS PART OF A REVIEW PROCEDURE FOR PUBLICATION, IT SHOULD NOT REPLACE THE EXPERTISE OF AN EXPERIENCED CRYSTALLOGRAPHIC REFEREE.

No syntax errors found. CIF dictionary Interpreting this report

Datablock: 4

```
Bond precision:   C-C = 0.0245 A          Wavelength=0.71073

Cell:             a=21.4359(6)     b=28.9930(8)       c=29.7998(8)
                  alpha=90         beta=102.671(1)    gamma=90
Temperature:      90 K

                Calculated                        Reported
Volume          18069.3(9)                        18069.3(9)
Space group     P 21/n                            P 1 21/n 1
Hall group      -P 2yn                            -P 2yn
Moiety formula  C180 H150 Ag23 Br7 P10 Pd2  C180 H150 Ag23 Br7 P10 Pd2
Sum formula     C180 H150 Ag23 Br7 P10 Pd2  C180 H150 Ag23 Br7 P10 Pd2
Mr              5875.75                           5875.87
Dx,g cm-3       2.160                             2.160
Z               4                                 4
Mu (mm-1)       4.305                             4.305
F000            11192.0                           11192.0
F000'           11100.44
h,k,lmax        19,26,27                          19,26,27
Nref            14337                             14280
Tmin,Tmax       0.995,0.996                       0.641,0.744
Tmin'           0.116

Correction method= # Reported T Limits: Tmin=0.641 Tmax=0.744
AbsCorr = MULTI-SCAN

Data completeness= 0.996            Theta(max)= 18.901

                                                    wR2(reflections)=
R(reflections)= 0.0351( 10844)                      0.0843( 14280)

S = 1.029                    Npar= 1951
```

The following ALERTS were generated. Each ALERT has the format
test-name_ALERT_alert-type_alert-level.
Click on the hyperlinks for more details of the test.

Alert level A

THETM01_ALERT_3_A The value of sine(theta_max)/wavelength is less than 0.550
Calculated sin(theta_max)/wavelength = 0.4558

Author Response: Despite several attempts over six months, resolution of cluster4's diffraction could not be improved better than 1.10 A.

Alert level B

PLAT088_ALERT_3_B Poor Data / Parameter Ratio .... 7.35 Note

Author Response: Despite several attempts over six months, resolution of cluster4's diffraction could not be improved better than 1.10 A.

PLAT342_ALERT_3_B Low Bond Precision on C-C Bonds .... 0.02454 Ang.

Author Response: Few phenyl rings of PPh3 ligands have disorder.

PLAT601_ALERT_2_B Unit Cell Contains Solvent Accessible VOIDS of . 169 Ang**3

Author Response: Cluster 4 has very few low electron density q peaks (1.300, 1.070, 0.800, 0.780, 0.760, 0.680, 0.630)outside main structural frame structure. These q peaks could be solvent of crystallization with fractions of occupancy.

Alert level C

```
PLAT213_ALERT_2_C Atom C71              has ADP max/min Ratio .....         3.2 prolat
PLAT220_ALERT_2_C NonSolvent    Resd 1   C    Ueq(max)/Ueq(min) Range       4.2 Ratio
PLAT234_ALERT_4_C Large Hirshfeld Difference P011     --C160     .        0.17 Ang.
PLAT234_ALERT_4_C Large Hirshfeld Difference C1       --C14      .        0.23 Ang.
PLAT234_ALERT_4_C Large Hirshfeld Difference C1       --C108     .        0.20 Ang.
PLAT234_ALERT_4_C Large Hirshfeld Difference C8       --C54      .        0.21 Ang.
PLAT234_ALERT_4_C Large Hirshfeld Difference C10      --C103     .        0.23 Ang.
PLAT234_ALERT_4_C Large Hirshfeld Difference C13      --C115     .        0.19 Ang.
PLAT234_ALERT_4_C Large Hirshfeld Difference C15      --C91      .        0.21 Ang.
PLAT234_ALERT_4_C Large Hirshfeld Difference C20      --C52      .        0.21 Ang.
PLAT234_ALERT_4_C Large Hirshfeld Difference C36      --C90      .        0.20 Ang.
PLAT234_ALERT_4_C Large Hirshfeld Difference C65      --C114     .        0.24 Ang.
PLAT234_ALERT_4_C Large Hirshfeld Difference C72      --C138     .        0.24 Ang.
PLAT234_ALERT_4_C Large Hirshfeld Difference C73      --C151     .        0.25 Ang.
PLAT234_ALERT_4_C Large Hirshfeld Difference C73      --C176     .        0.20 Ang.
PLAT234_ALERT_4_C Large Hirshfeld Difference C79      --C103     .        0.24 Ang.
```

```
PLAT234_ALERT_4_C Large Hirshfeld Difference C101     --C171     .       0.24 Ang.
PLAT234_ALERT_4_C Large Hirshfeld Difference C106     --C107     .       0.19 Ang.
PLAT234_ALERT_4_C Large Hirshfeld Difference C106     --C146     .       0.21 Ang.
PLAT234_ALERT_4_C Large Hirshfeld Difference C115     --C119     .       0.23 Ang.
PLAT234_ALERT_4_C Large Hirshfeld Difference C117     --C127     .       0.24 Ang.
PLAT234_ALERT_4_C Large Hirshfeld Difference C142     --C160     .       0.18 Ang.
PLAT234_ALERT_4_C Large Hirshfeld Difference C147     --C155     .       0.24 Ang.
PLAT234_ALERT_4_C Large Hirshfeld Difference C147     --C162     .       0.22 Ang.
PLAT241_ALERT_2_C High   'MainMol' Ueq as Compared to Neighbors of        C21 Check
PLAT241_ALERT_2_C High   'MainMol' Ueq as Compared to Neighbors of        C79 Check
PLAT241_ALERT_2_C High   'MainMol' Ueq as Compared to Neighbors of       C159 Check
PLAT241_ALERT_2_C High   'MainMol' Ueq as Compared to Neighbors of       C167 Check
PLAT242_ALERT_2_C Low    'MainMol' Ueq as Compared to Neighbors of         C4 Check
PLAT242_ALERT_2_C Low    'MainMol' Ueq as Compared to Neighbors of        C22 Check
PLAT242_ALERT_2_C Low    'MainMol' Ueq as Compared to Neighbors of        C32 Check
PLAT242_ALERT_2_C Low    'MainMol' Ueq as Compared to Neighbors of       C110 Check
PLAT331_ALERT_2_C Small Aver Phenyl C-C Dist C10      --C103     .       1.37 Ang.
PLAT331_ALERT_2_C Small Aver Phenyl C-C Dist C17      --C121     .       1.37 Ang.
```

Alert level G

```
PLAT003_ALERT_2_G Number of Uiso or Uij Restrained non-H Atoms ...          7 Report
PLAT083_ALERT_2_G SHELXL Second Parameter in WGHT  Unusually Large      95.85 Why ?
PLAT177_ALERT_4_G The CIF-Embedded .res File Contains DELU Records          1 Report
PLAT178_ALERT_4_G The CIF-Embedded .res File Contains SIMU Records          1 Report
PLAT186_ALERT_4_G The CIF-Embedded .res File Contains ISOR Records          1 Report
PLAT187_ALERT_4_G The CIF-Embedded .res File Contains RIGU Records          4 Report
PLAT232_ALERT_2_G Hirshfeld Test Diff (M-X)  Ag0J     --Br3      .        8.8 s.u.
PLAT232_ALERT_2_G Hirshfeld Test Diff (M-X)  Ag0O     --Br5      .        8.3 s.u.
PLAT720_ALERT_4_G Number of Unusual/Non-Standard Labels ..........         34 Note
PLAT767_ALERT_4_G INS Embedded LIST 6 Instruction Should be LIST 4     Please Check
PLAT793_ALERT_4_G Model has Chirality at P00Y        (Centro SPGR)          S Verify
PLAT793_ALERT_4_G Model has Chirality at P012        (Centro SPGR)          S Verify
PLAT860_ALERT_3_G Number of Least-Squares Restraints ............         117 Note
```

```
  1 ALERT level A = Most likely a serious problem - resolve or explain
  3 ALERT level B = A potentially serious problem, consider carefully
 34 ALERT level C = Check. Ensure it is not caused by an omission or oversight
 13 ALERT level G = General information/check it is not something unexpected

  0 ALERT type 1 CIF construction/syntax error, inconsistent or missing data
 17 ALERT type 2 Indicator that the structure model may be wrong or deficient
  4 ALERT type 3 Indicator that the structure quality may be low
 30 ALERT type 4 Improvement, methodology, query or suggestion
  0 ALERT type 5 Informative message, check
```

It is advisable to attempt to resolve as many as possible of the alerts in all categories. Often the minor alerts point to easily fixed oversights, errors and omissions in your CIF or refinement strategy, so attention to these fine details can be worthwhile. In order to resolve some of the more serious problems it may be necessary to carry out additional measurements or structure refinements. However, the purpose of your study may justify the reported deviations and the more serious of these should normally be commented upon in the discussion or experimental section of a paper or in the "special_details" fields of the CIF. checkCIF was carefully designed to identify outliers and unusual parameters, but every test has its limitations and alerts that are not important in a particular case may appear. Conversely, the absence of alerts does not guarantee there are no aspects of the results needing attention. It is up to the individual to critically assess their own results and, if necessary, seek expert advice.

Publication of your CIF in IUCr journals

A basic structural check has been run on your CIF. These basic checks will be run on all CIFs submitted for publication in IUCr journals (*Acta Crystallographica*, *Journal of Applied Crystallography*, *Journal of Synchrotron Radiation*); however, if you intend to submit to *Acta Crystallographica Section C* or *E* or *IUCrData*, you should make sure that full publication checks are run on the final version of your CIF prior to submission.

Publication of your CIF in other journals

Please refer to the *Notes for Authors* of the relevant journal for any special instructions relating to CIF submission.

PLATON version of 12/09/2022; check.def file version of 09/08/2022

Datablock 4 - ellipsoid plot

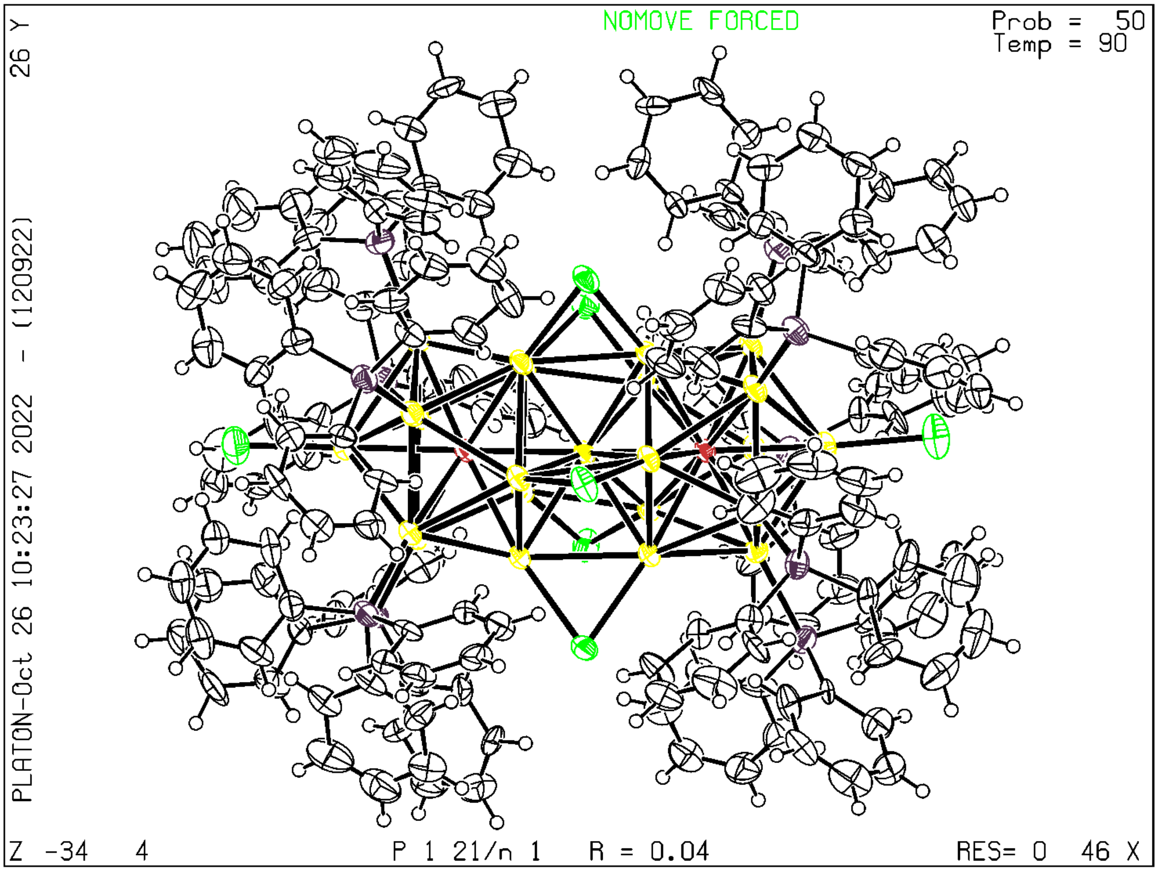

Supplement: Supplementary file 7 — Supplementary Data 4 [file 42004_2023_854_MOESM7_ESM.pdf]
