# Supplementary material for: Key factors for connecting silver-based icosahedral superatoms by vertex sharing
Source: Commun Chem. 2023 Mar 28;6:57. doi: 10.1038/s42004-023-00854-0 (PMC10050180; doi:10.1038/s42004-023-00854-0)
Supplement: Supplementary file 8 — Supplementary Data 5 [file 42004_2023_854_MOESM8_ESM.pdf]

element	*x*	*y*	*z*
Pt	-0.00826743110785	0.00986700086782	5.44579389447565
Ag	-0.00095180664651	-0.00597798885208	10.72240313329300
Ag	-0.45224854011211	4.73409447521514	7.65733465927448
Ag	4.36133144667696	1.89364187892504	7.63137426330821
Ag	3.14667554628917	-3.55813484517308	7.62140088482148
Ag	-2.42311237298453	-4.08625431887980	7.63238733891470
Ag	-4.64907644664449	1.05096878609765	7.64478773812263
Ag	-3.25241936900750	3.40884034113633	2.90626997588009
Ag	2.20304936375773	4.16367665685748	2.90272163933155
Ag	4.61353490609762	-0.80189976919632	2.88841347124561
Ag	0.64213999673643	-4.63778293806019	2.88523118665031
Ag	-4.23317747685269	-2.02262453944406	2.89757576246165
Ag	-0.00449764737005	0.01944478722634	0.00000000000000
Cl	0.01202215768006	-0.03993147464087	15.41822996142630
Cl	-6.05161372592058	6.05893711482293	0.00000000000000
Cl	3.76056994519357	7.69974387123553	0.00000000000000
Cl	8.48149107804815	-1.04506745085303	0.00000000000000
Cl	1.52655710184336	-8.41965598336067	0.00000000000000
Cl	-7.60135680333837	-3.92103871837429	0.00000000000000
P	-0.78891820366147	8.99222455252042	9.57638306127105
P	8.33190890688355	3.51341919458304	9.51215323289544
P	5.94169899845143	-6.82348072778779	9.47847945835685
P	-4.67231117580028	-7.72898778769220	9.53245866164673
P	-8.80734046305908	2.05901008652147	9.56080753107072
C	2.21601812624056	10.66824501621160	10.18931742683270
C	3.78940324648641	11.19535532514860	8.09688968930654
H	3.24139360597050	10.56040102848450	6.18508497093392
C	6.04473346494943	12.54530881855330	8.43130789274995
H	7.25731479787013	12.94281787007120	6.78609924717059
C	6.76661823175367	13.36331559579780	10.85298442013390
H	8.54284903995685	14.42239726681550	11.10959281763840
C	5.22993066270184	12.80839615449100	12.94140048918230
H	5.79293564919038	13.42339605653880	14.85050098544710
C	2.96199676810764	11.46563436891640	12.61830276686780
H	1.76608473802446	11.04395278565340	14.26885422394050
C	-2.35645501445169	8.93531873844432	12.69217193702620
C	-1.99195736120686	6.75879327929599	14.19224084262700
H	-0.90804737622676	5.13135691991081	13.46489160695970

C	-3.01754290333639	6.61727545287157	16.63457482706650
H	-2.70485643397617	4.88679902456090	17.75024492300390
C	-4.43030549849447	8.64751460780548	17.59710560841910
H	-5.24285480170104	8.54300901189025	19.51368546318030
C	-4.82719139187760	10.80755699258420	16.10252754614570
H	-5.95601931076948	12.39680021349010	16.84023647746460
C	-3.80227713513271	10.95545534655530	13.65942565340940
H	-4.14860173176922	12.64782764272810	12.49823118105460
C	8.68590564640247	-7.67896546963544	7.49857020684808
C	9.35463709464149	-6.08056431472529	5.47877705219571
H	8.24763588215372	-4.35265359327255	5.10250551525739
C	11.38641277378280	-6.69457805832323	3.88723420768168
H	11.83643865408290	-5.42158271574699	2.30288194096273
C	12.77474073887350	-8.91480962950417	4.30542504927384
H	14.36181615447480	-9.40964676698773	3.04782527566151
C	12.11078618968320	-10.53398277707710	6.30555035031250
H	13.17578071682320	-12.29708185200500	6.62175318732672
C	10.06798374948000	-9.93304384678959	7.88491518455928
H	9.51850719353123	-11.24982934429250	9.40338723960616
C	4.50762632163752	-9.96046643636908	10.03923278212300
C	3.61289326745204	-11.31456581789110	7.91744272612826
H	3.73419683694222	-10.47817800326150	6.00907045867715
C	2.58213797894476	-13.73650624473260	8.22104492797123
H	1.89240313919584	-14.77482994345170	6.55310159199104
C	2.40681605978593	-14.82349861759210	10.63829636806530
H	1.59208757385459	-16.72745688907540	10.86912691741000
C	3.25276985745987	-13.46757066440830	12.75408403450410
H	3.10125529310437	-14.29556966786670	14.65974329392950
C	4.29959149262087	-11.04456627366210	12.46318087839480
H	4.96169386541132	-9.99784418134001	14.13589947793900
C	7.18033846750399	-5.90621577709461	12.60849076011880
C	5.61635675088320	-4.37330169556766	14.13216430409980
H	3.78455193026743	-3.67456493501409	13.41920952937650
C	6.37139151178466	-3.69296712635302	16.58303640106330
H	5.10895547655565	-2.48562296637784	17.71816240392520
C	8.70443415834659	-4.52995527287599	17.53179535471050
H	9.30488633516496	-3.99630111459296	19.45492049778420
C	10.28422645778380	-6.03252558170092	16.01460459097530
H	12.12886390194610	-6.67635539271500	16.74013681759300

C	9.53488432854433	-6.71524769161267	13.56222335859200
H	10.80416548438000	-7.86973149876428	12.38324827229030
C	-4.69154908172153	-10.60451021281820	7.55237016125690
C	-2.97318085557199	-10.77814976096430	5.52666692511286
H	-1.64711644818887	-9.21510822514358	5.14214413127850
C	-2.97140298198498	-12.90420570721270	3.93923389946843
H	-1.63011698379865	-12.96737813240880	2.34759054619280
C	-4.68672096620512	-14.88080853313470	4.36585058218901
H	-4.70162977094547	-16.54520307398670	3.11098863972293
C	-6.42249165508620	-14.71684492758100	6.37159288662866
H	-7.79704276650336	-16.24942062844480	6.69544061337295
C	-6.44019349971385	-12.58499998580110	7.94764035973164
H	-7.85343125271384	-12.43980872473830	9.47151929689456
C	-8.08753363048056	-7.30897150738490	10.14181915048100
C	-9.66501283195243	-6.79292820300429	8.04978694133675
H	-8.84435217981060	-6.59217347766149	6.14075345819988
C	-12.28318331364690	-6.55588073656298	8.38016750365800
H	-13.49343952820190	-6.15432972482012	6.73436042596866
C	-13.35296971506790	-6.80458045830989	10.79845025600430
H	-15.41229888805990	-6.61323936543320	11.05346264061340
C	-11.78813023814890	-7.27546765274710	12.88684131561860
H	-12.60949327545460	-7.45107514786894	14.79329732991830
C	-9.16414367836301	-7.52747311141766	12.56746675095020
H	-7.95333970893529	-7.90318384205971	14.21771217671130
C	-3.38091337158520	-8.64375525862886	12.64114838569260
C	-2.37596740236829	-6.69480910892685	14.16071529156810
H	-2.27737064896088	-4.73370784513816	13.45501713127380
C	-1.46576254597836	-7.21925234595708	16.59626661055380
H	-0.68461012905778	-5.65554792703065	17.72861036323660
C	-1.53975551482657	-9.70160292544948	17.53211606430330
H	-0.82279465650298	-10.12250721346970	19.44367331355160
C	-2.50971889906073	-11.65652727873830	16.01753618786820
H	-2.54956923836397	-13.61327219874650	16.73448914337530
C	-3.42280539045622	-11.13798601674880	13.58083571597950
H	-4.15455825003624	-12.69066538499560	12.40318038604770
C	-2.47677705687042	11.33652576616500	7.61648287127044
C	-3.92623040457934	10.47519026830620	5.55722115909365
H	-4.03976210411276	8.43470464385022	5.13828937442018
C	-5.18483652786803	12.19697164259730	3.97875629046443

H	-6.27227484052541	11.46388153868430	2.36135439259792
C	-5.00875942756725	14.80062961624290	4.44949096765061
H	-5.98244553337695	16.15842741667610	3.20316658119497
C	-3.54792511494195	15.68144244533560	6.48740908531368
H	-3.37415311885972	17.72735155517570	6.84352073669440
C	-2.27233214731973	13.96438275447920	8.05376957541321
H	-1.07289686638432	14.67549985564670	9.60194560110362
C	10.01781063784560	5.85626135604063	7.54762447373010
C	8.73138221364689	6.97530054390611	5.50298029029876
H	6.75396725701205	6.45045537967684	5.09777721869877
C	9.96413481359996	8.71493235595404	3.92344015288669
H	8.91625501415381	9.53000383736292	2.31904021203866
C	12.49738302278370	9.35486077517732	4.37625967526238
H	13.47624030098470	10.70689855389920	3.12779254477209
C	13.80460273783080	8.23291319150693	6.40019551874673
H	15.80503416924990	8.70482251779607	6.74485175572827
C	12.58158155451870	6.47969123068089	7.96897839618773
H	13.64191871267150	5.55623324686209	9.50631075053549
C	10.86286153594390	1.17759143720867	10.09792136253430
C	11.88850380981770	-0.09905207521729	7.98819356712023
H	11.14273417377370	0.26934116979541	6.07384211016771
C	13.87262421901340	-1.82572751159673	8.30973662585365
H	14.65781148895690	-2.80733553851257	6.64978984043113
C	14.83676019376280	-2.31880372721553	10.73520883595200
H	16.39451738038630	-3.68029982538316	10.98257469446700
C	13.79462004797460	-1.08702310584855	12.83951767493100
H	14.52374506333360	-1.47843802181938	14.75121446548170
C	11.81544146610220	0.65622114531544	12.52982192054580
H	11.01625888358040	1.61757868313270	14.19357799548500
C	7.81130441561034	4.97324896830733	12.63763621363820
C	5.86252052112683	3.94809652834256	14.14391896759410
H	4.64933964901184	2.41260519787728	13.41877453483710
C	5.41680398836547	4.87567336283217	16.58854361704690
H	3.87176262141372	4.03799772222353	17.70631681306670
C	6.90947276332377	6.84863692215893	17.54816271517900
H	6.56089893155787	7.58774737870388	19.46565515758960
C	8.83488593114966	7.89747868072363	16.04901696476530
H	9.99668351952458	9.46359545734436	16.78503094556020
C	9.28633757277001	6.97264707305761	13.60340964424650

H	10.78482187721370	7.83008650574807	12.44051267716350
C	-11.55368495746030	1.17884736757857	7.59202532622709
C	-11.17603708095790	-0.46092550004873	5.52997911823295
H	-9.26954695277781	-1.20016645259162	5.11702484580425
C	-13.19701181308080	-1.12107084299060	3.94281015924847
H	-12.83010480009760	-2.37880046221868	2.32443242639521
C	-15.62053756488450	-0.14998315127303	4.40682525788239
H	-17.20807005080030	-0.65233340904038	3.15289704964008
C	-16.01378982923580	1.50486338150983	6.44891219361583
H	-17.90745510643030	2.29998953019877	6.80190530849987
C	-13.99169424182840	2.18261862919980	8.02436220893880
H	-14.30462504456580	3.53740694431137	9.57627166905627
C	-9.47755887834441	5.43446626080090	10.16995250652430
C	-9.50619263588908	7.08959814669794	8.07393170863711
H	-9.07907377426872	6.36868465603246	6.16244170999374
C	-10.09869263235150	9.65061831650208	8.40560582955151
H	-10.11351669399660	10.92198135967140	6.75692992623688
C	-10.64481504858340	10.59402298258290	10.82793138029130
H	-11.10765068459500	12.60980620795980	11.08238091282530
C	-10.57748178648020	8.96568158610166	12.91984260793150
H	-10.98064708466350	9.69467431104042	14.82930780228690
C	-9.99582937569404	6.39446617904891	12.59983737665730
H	-9.95271763047483	5.13192707095098	14.25429773686380
C	-9.24604011374539	0.54705814068398	12.67508477333460
C	-7.06835159564315	0.21437795003723	14.18005946717780
H	-5.18288018974028	0.74393531775427	13.46128467106410
C	-7.25800496187421	-0.81780162747196	16.61643393938170
H	-5.51772091280241	-1.06164844039007	17.73484731162570
C	-9.62743354961512	-1.53956546301601	17.56949633567830
H	-9.78231999763759	-2.35685238989360	19.48060970939680
C	-11.80016825605010	-1.23990941877238	16.07121992951190
H	-13.66286237582260	-1.82474838984963	16.80058109412120
C	-11.61651977576450	-0.20810152805550	13.63330110306730
H	-13.33144631842950	-0.01018728840308	12.46965009093830
Pt	-0.00826743110785	0.00986700086782	-5.44579389447565
Ag	-0.00095180664651	-0.00597798885208	-10.72240313329300
Ag	-0.45224854011211	4.73409447521514	-7.65733465927448
Ag	4.36133144667696	1.89364187892504	-7.63137426330821
Ag	3.14667554628917	-3.55813484517308	-7.62140088482148

Ag	-2.42311237298453	-4.08625431887980	-7.63238733891470
Ag	-4.64907644664449	1.05096878609765	-7.64478773812263
Ag	-3.25241936900750	3.40884034113633	-2.90626997588009
Ag	2.20304936375773	4.16367665685748	-2.90272163933155
Ag	4.61353490609762	-0.80189976919632	-2.88841347124561
Ag	0.64213999673643	-4.63778293806019	-2.88523118665031
Ag	-4.23317747685269	-2.02262453944406	-2.89757576246165
Cl	0.01202215768006	-0.03993147464087	-15.41822996142630
P	-0.78891820366147	8.99222455252042	-9.57638306127105
P	8.33190890688355	3.51341919458304	-9.51215323289544
P	5.94169899845143	-6.82348072778779	-9.47847945835685
P	-4.67231117580028	-7.72898778769220	-9.53245866164673
P	-8.80734046305908	2.05901008652147	-9.56080753107072
C	2.21601812624056	10.66824501621160	-10.18931742683270
C	3.78940324648641	11.19535532514860	-8.09688968930654
H	3.24139360597050	10.56040102848450	-6.18508497093392
C	6.04473346494943	12.54530881855330	-8.43130789274995
H	7.25731479787013	12.94281787007120	-6.78609924717059
C	6.76661823175367	13.36331559579780	-10.85298442013390
H	8.54284903995685	14.42239726681550	-11.10959281763840
C	5.22993066270184	12.80839615449100	-12.94140048918230
H	5.79293564919038	13.42339605653880	-14.85050098544710
C	2.96199676810764	11.46563436891640	-12.61830276686780
H	1.76608473802446	11.04395278565340	-14.26885422394050
C	-2.35645501445169	8.93531873844432	-12.69217193702620
C	-1.99195736120686	6.75879327929599	-14.19224084262700
H	-0.90804737622676	5.13135691991081	-13.46489160695970
C	-3.01754290333639	6.61727545287157	-16.63457482706650
H	-2.70485643397617	4.88679902456090	-17.75024492300390
C	-4.43030549849447	8.64751460780548	-17.59710560841910
H	-5.24285480170104	8.54300901189025	-19.51368546318030
C	-4.82719139187760	10.80755699258420	-16.10252754614570
H	-5.95601931076948	12.39680021349010	-16.84023647746460
C	-3.80227713513271	10.95545534655530	-13.65942565340940
H	-4.14860173176922	12.64782764272810	-12.49823118105460
C	8.68590564640247	-7.67896546963544	-7.49857020684808
C	9.35463709464149	-6.08056431472529	-5.47877705219571
H	8.24763588215372	-4.35265359327255	-5.10250551525739
C	11.38641277378280	-6.69457805832323	-3.88723420768168

H	11.83643865408290	-5.42158271574699	-2.30288194096273
C	12.77474073887350	-8.91480962950417	-4.30542504927384
H	14.36181615447480	-9.40964676698773	-3.04782527566151
C	12.11078618968320	-10.53398277707710	-6.30555035031250
H	13.17578071682320	-12.29708185200500	-6.62175318732672
C	10.06798374948000	-9.93304384678959	-7.88491518455928
H	9.51850719353123	-11.24982934429250	-9.40338723960616
C	4.50762632163752	-9.96046643636908	-10.03923278212300
C	3.61289326745204	-11.31456581789110	-7.91744272612826
H	3.73419683694222	-10.47817800326150	-6.00907045867715
C	2.58213797894476	-13.73650624473260	-8.22104492797123
H	1.89240313919584	-14.77482994345170	-6.55310159199104
C	2.40681605978593	-14.82349861759210	-10.63829636806530
H	1.59208757385459	-16.72745688907540	-10.86912691741000
C	3.25276985745987	-13.46757066440830	-12.75408403450410
H	3.10125529310437	-14.29556966786670	-14.65974329392950
C	4.29959149262087	-11.04456627366210	-12.46318087839480
H	4.96169386541132	-9.99784418134001	-14.13589947793900
C	7.18033846750399	-5.90621577709461	-12.60849076011880
C	5.61635675088320	-4.37330169556766	-14.13216430409980
H	3.78455193026743	-3.67456493501409	-13.41920952937650
C	6.37139151178466	-3.69296712635302	-16.58303640106330
H	5.10895547655565	-2.48562296637784	-17.71816240392520
C	8.70443415834659	-4.52995527287599	-17.53179535471050
H	9.30488633516496	-3.99630111459296	-19.45492049778420
C	10.28422645778380	-6.03252558170092	-16.01460459097530
H	12.12886390194610	-6.67635539271500	-16.74013681759300
C	9.53488432854433	-6.71524769161267	-13.56222335859200
H	10.80416548438000	-7.86973149876428	-12.38324827229030
C	-4.69154908172153	-10.60451021281820	-7.55237016125690
C	-2.97318085557199	-10.77814976096430	-5.52666692511286
H	-1.64711644818887	-9.21510822514358	-5.14214413127850
C	-2.97140298198498	-12.90420570721270	-3.93923389946843
H	-1.63011698379865	-12.96737813240880	-2.34759054619280
C	-4.68672096620512	-14.88080853313470	-4.36585058218901
H	-4.70162977094547	-16.54520307398670	-3.11098863972293
C	-6.42249165508620	-14.71684492758100	-6.37159288662866
H	-7.79704276650336	-16.24942062844480	-6.69544061337295
C	-6.44019349971385	-12.58499998580110	-7.94764035973164

H	-7.85343125271384	-12.43980872473830	-9.47151929689456
C	-8.08753363048056	-7.30897150738490	-10.14181915048100
C	-9.66501283195243	-6.79292820300429	-8.04978694133675
H	-8.84435217981060	-6.59217347766149	-6.14075345819988
C	-12.28318331364690	-6.55588073656298	-8.38016750365800
H	-13.49343952820190	-6.15432972482012	-6.73436042596866
C	-13.35296971506790	-6.80458045830989	-10.79845025600430
H	-15.41229888805990	-6.61323936543320	-11.05346264061340
C	-11.78813023814890	-7.27546765274710	-12.88684131561860
H	-12.60949327545460	-7.45107514786894	-14.79329732991830
C	-9.16414367836301	-7.52747311141766	-12.56746675095020
H	-7.95333970893529	-7.90318384205971	-14.21771217671130
C	-3.38091337158520	-8.64375525862886	-12.64114838569260
C	-2.37596740236829	-6.69480910892685	-14.16071529156810
H	-2.27737064896088	-4.73370784513816	-13.45501713127380
C	-1.46576254597836	-7.21925234595708	-16.59626661055380
H	-0.68461012905778	-5.65554792703065	-17.72861036323660
C	-1.53975551482657	-9.70160292544948	-17.53211606430330
H	-0.82279465650298	-10.12250721346970	-19.44367331355160
C	-2.50971889906073	-11.65652727873830	-16.01753618786820
H	-2.54956923836397	-13.61327219874650	-16.73448914337530
C	-3.42280539045622	-11.13798601674880	-13.58083571597950
H	-4.15455825003624	-12.69066538499560	-12.40318038604770
C	-2.47677705687042	11.33652576616500	-7.61648287127044
C	-3.92623040457934	10.47519026830620	-5.55722115909365
H	-4.03976210411276	8.43470464385022	-5.13828937442018
C	-5.18483652786803	12.19697164259730	-3.97875629046443
H	-6.27227484052541	11.46388153868430	-2.36135439259792
C	-5.00875942756725	14.80062961624290	-4.44949096765061
H	-5.98244553337695	16.15842741667610	-3.20316658119497
C	-3.54792511494195	15.68144244533560	-6.48740908531368
H	-3.37415311885972	17.72735155517570	-6.84352073669440
C	-2.27233214731973	13.96438275447920	-8.05376957541321
H	-1.07289686638432	14.67549985564670	-9.60194560110362
C	10.01781063784560	5.85626135604063	-7.54762447373010
C	8.73138221364689	6.97530054390611	-5.50298029029876
H	6.75396725701205	6.45045537967684	-5.09777721869877
C	9.96413481359996	8.71493235595404	-3.92344015288669
H	8.91625501415381	9.53000383736292	-2.31904021203866

C	12.49738302278370	9.35486077517732	-4.37625967526238
H	13.47624030098470	10.70689855389920	-3.12779254477209
C	13.80460273783080	8.23291319150693	-6.40019551874673
H	15.80503416924990	8.70482251779607	-6.74485175572827
C	12.58158155451870	6.47969123068089	-7.96897839618773
H	13.64191871267150	5.55623324686209	-9.50631075053549
C	10.86286153594390	1.17759143720867	-10.09792136253430
C	11.88850380981770	-0.09905207521729	-7.98819356712023
H	11.14273417377370	0.26934116979541	-6.07384211016771
C	13.87262421901340	-1.82572751159673	-8.30973662585365
H	14.65781148895690	-2.80733553851257	-6.64978984043113
C	14.83676019376280	-2.31880372721553	-10.73520883595200
H	16.39451738038630	-3.68029982538316	-10.98257469446700
C	13.79462004797460	-1.08702310584855	-12.83951767493100
H	14.52374506333360	-1.47843802181938	-14.75121446548170
C	11.81544146610220	0.65622114531544	-12.52982192054580
H	11.01625888358040	1.61757868313270	-14.19357799548500
C	7.81130441561034	4.97324896830733	-12.63763621363820
C	5.86252052112683	3.94809652834256	-14.14391896759410
H	4.64933964901184	2.41260519787728	-13.41877453483710
C	5.41680398836547	4.87567336283217	-16.58854361704690
H	3.87176262141372	4.03799772222353	-17.70631681306670
C	6.90947276332377	6.84863692215893	-17.54816271517900
H	6.56089893155787	7.58774737870388	-19.46565515758960
C	8.83488593114966	7.89747868072363	-16.04901696476530
H	9.99668351952458	9.46359545734436	-16.78503094556020
C	9.28633757277001	6.97264707305761	-13.60340964424650
H	10.78482187721370	7.83008650574807	-12.44051267716350
C	-11.55368495746030	1.17884736757857	-7.59202532622709
C	-11.17603708095790	-0.46092550004873	-5.52997911823295
H	-9.26954695277781	-1.20016645259162	-5.11702484580425
C	-13.19701181308080	-1.12107084299060	-3.94281015924847
H	-12.83010480009760	-2.37880046221868	-2.32443242639521
C	-15.62053756488450	-0.14998315127303	-4.40682525788239
H	-17.20807005080030	-0.65233340904038	-3.15289704964008
C	-16.01378982923580	1.50486338150983	-6.44891219361583
H	-17.90745510643030	2.29998953019877	-6.80190530849987
C	-13.99169424182840	2.18261862919980	-8.02436220893880
H	-14.30462504456580	3.53740694431137	-9.57627166905627

C	-9.47755887834441	5.43446626080090	-10.16995250652430
C	-9.50619263588908	7.08959814669794	-8.07393170863711
H	-9.07907377426872	6.36868465603246	-6.16244170999374
C	-10.09869263235150	9.65061831650208	-8.40560582955151
H	-10.11351669399660	10.92198135967140	-6.75692992623688
C	-10.64481504858340	10.59402298258290	-10.82793138029130
H	-11.10765068459500	12.60980620795980	-11.08238091282530
C	-10.57748178648020	8.96568158610166	-12.91984260793150
H	-10.98064708466350	9.69467431104042	-14.82930780228690
C	-9.99582937569404	6.39446617904891	-12.59983737665730
H	-9.95271763047483	5.13192707095098	-14.25429773686380
C	-9.24604011374539	0.54705814068398	-12.67508477333460
C	-7.06835159564315	0.21437795003723	-14.18005946717780
H	-5.18288018974028	0.74393531775427	-13.46128467106410
C	-7.25800496187421	-0.81780162747196	-16.61643393938170
H	-5.51772091280241	-1.06164844039007	-17.73484731162570
C	-9.62743354961512	-1.53956546301601	-17.56949633567830
H	-9.78231999763759	-2.35685238989360	-19.48060970939680
C	-11.80016825605010	-1.23990941877238	-16.07121992951190
H	-13.66286237582260	-1.82474838984963	-16.80058109412120
C	-11.61651977576450	-0.20810152805550	-13.63330110306730
H	-13.33144631842950	-0.01018728840308	-12.46965009093830
