# Supplementary material for: Key factors for connecting silver-based icosahedral superatoms by vertex sharing
Source: Commun Chem. 2023 Mar 28;6:57. doi: 10.1038/s42004-023-00854-0 (PMC10050180; doi:10.1038/s42004-023-00854-0)
Supplement: Supplementary file 9 — Supplementary Data 6 [file 42004_2023_854_MOESM9_ESM.pdf]

element	*x*	*y*	*z*
Pd	-0.00862023815215	0.00809644970865	5.41958666390627
Ag	-0.00406016319607	-0.00530192827805	10.68350838534880
Ag	-0.45688150085933	4.71365150469436	7.63682382786122
Ag	4.33842664404816	1.88775375226582	7.61083626093670
Ag	3.13677947673596	-3.54100372224677	7.60142499430734
Ag	-2.41010958026211	-4.06998875221582	7.61095281916374
Ag	-4.63193822312788	1.04455897565197	7.62366345764928
Ag	-3.24715070339556	3.40016866954882	2.90368186840627
Ag	2.19603856114554	4.15488944129853	2.90003888595507
Ag	4.60396518801192	-0.80080846601737	2.88573854067464
Ag	0.64551134897133	-4.62859661879867	2.88246490961571
Ag	-4.22368473621556	-2.01996694593081	2.89439988911070
Ag	-0.00355867741668	0.01826431490981	0.00000000000000
Cl	0.00997954224871	-0.04022995938954	15.39465586244450
Cl	-6.06375279221555	6.05933125516261	0.00000000000000
Cl	3.74496474027780	7.71746662187021	0.00000000000000
Cl	8.49208730571143	-1.02882550063910	0.00000000000000
Cl	1.55029934407928	-8.42625002125615	0.00000000000000
Cl	-7.60590050916158	-3.93874969910896	0.00000000000000
P	-0.78623669471530	8.99223632555103	9.56400242142370
P	8.33049857059052	3.51016867182921	9.49777157578789
P	5.94203756594980	-6.82506196279971	9.46383602951814
P	-4.67501138478028	-7.72711195284914	9.51785990285271
P	-8.80843411092783	2.06398191855454	9.54607521452226
C	2.21866448636756	10.66880442848530	10.17424613526120
C	3.78707345724239	11.20266908362830	8.07966821084503
H	3.23491732675090	10.57370597391510	6.16700449233904
C	6.04262834803072	12.55255538539110	8.41284568299089
H	7.25076368786701	12.95602977054570	6.76583521295360
C	6.77006742594516	13.36354035204290	10.83521799096430
H	8.54645651721893	14.42259645082720	11.09074174355420
C	5.23856670036791	12.80158295780760	12.92561994505670
H	5.80560250972085	13.41145129572630	14.83518359784560
C	2.97051397178433	11.45875575151000	12.60394569880650
H	1.77900307611586	11.03132354662460	14.25620046957900
C	-2.35126998612791	8.93541078537363	12.68089043516240
C	-1.98617323233902	6.75833210143775	14.17993438679620
H	-0.90222423958727	5.13124962026774	13.45168177775310

C	-3.01051972468452	6.61559906626392	16.62273608659000
H	-2.69745590648645	4.88445364122798	17.73727915902510
C	-4.42274975099140	8.64547397169684	17.58675412787860
H	-5.23433060783416	8.54022659050137	19.50370272871820
C	-4.82048755636771	10.80610704882220	16.09314257291690
H	-5.94908870273952	12.39496310442640	16.83206054627660
C	-3.79679344700640	10.95511527120680	13.64965142563520
H	-4.14348168420966	12.64801987708120	12.48934202539190
C	8.68827566405248	-7.67597742944151	7.48524757809421
C	9.35798782422389	-6.07347996072935	5.46901737341161
H	8.25131705111752	-4.34479687980251	5.09530770750126
C	11.39069288023450	-6.68384813976144	3.87734728389708
H	11.84099228520170	-5.40750963135047	2.29581897820452
C	12.77927368064700	-8.90457703061041	4.29193890133399
H	14.36723316726840	-9.39648732528264	3.03430900593522
C	12.11460863118090	-10.52784640973140	6.28853330375102
H	13.17989504431890	-12.29127709516030	6.60191066476971
C	10.07080115072620	-9.93051066397675	7.86791376300996
H	9.52085054745349	-11.25023793995370	9.38362873476431
C	4.50942041048992	-9.96316223355167	10.01999827415170
C	3.62328093286744	-11.31876232944020	7.89539456972238
H	3.75294102327926	-10.48434681278360	5.98666679107475
C	2.59060002344162	-13.74017845127770	8.19659145623946
H	1.90792452483548	-14.77968238706520	6.52647746822498
C	2.40440191702840	-14.82525304365410	10.61389626239970
H	1.58786904171877	-16.72867112470820	10.84269078719090
C	3.24207480668294	-13.46801507007120	12.73216763604320
H	3.08232398264078	-14.29443760491780	14.63781886228200
C	4.29093791692981	-11.04561850608470	12.44387065231060
H	4.94655058263982	-9.99805084412815	14.11859823226280
C	7.17760822947646	-5.91034766039514	12.59570940981600
C	5.61224828603114	-4.37798028601991	14.11843078373000
H	3.78091929604427	-3.67918604255884	13.40409581845690
C	6.36460274936473	-3.69855240014898	16.57040400510830
H	5.10109623098533	-2.49137157980309	17.70452742003790
C	8.69661693547312	-4.53606703872217	17.52117897241260
H	9.29512850609167	-4.00326813987573	19.44514460808510
C	10.27802494047420	-6.03797149695465	16.00488792912610
H	12.12191088351060	-6.68208519209853	16.73211446442320

C	9.53130259606047	-6.71965853284840	13.55147509983860
H	10.80160031357140	-7.87388570738268	12.37332441645150
C	-4.68922378599403	-10.60310255355650	7.53877022166062
C	-2.96629157984920	-10.77602947480400	5.51684773771247
H	-1.63948063407393	-9.21283875391907	5.13522795884306
C	-2.95994905331867	-12.90183237734080	3.92921060683027
H	-1.61501295972371	-12.96393703966330	2.34063416792297
C	-4.67527649774245	-14.87922095974930	4.35191190416561
H	-4.68652064667120	-16.54361016476400	3.09699563258131
C	-6.41552194664633	-14.71622335172870	6.35386111281882
H	-7.79008751240679	-16.24947790621580	6.67447988412064
C	-6.43766390156154	-12.58461235117150	7.93010097522690
H	-7.85392543419122	-12.44044877260750	9.45126558489449
C	-8.09094926968796	-7.30968793381623	10.12339284567120
C	-9.66788826655028	-6.80179204155791	8.02882579779621
H	-8.84681395802775	-6.60696145345880	6.11926908014013
C	-12.28643661522870	-6.56611057872344	8.35716031741354
H	-13.49619581346450	-6.17168808456797	6.70928836436270
C	-13.35730016805750	-6.80757317217926	10.77569951149040
H	-15.41695914502290	-6.61755591187580	11.02896357525980
C	-11.79301096807040	-7.26986238301919	12.86647510345330
H	-12.61517668994830	-7.43997338095446	14.77309652884240
C	-9.16867192142819	-7.52054858571976	12.54930830763980
H	-7.95841989315557	-7.88942992302647	14.20152399507270
C	-3.38589606696929	-8.64048589306624	12.62779651139570
C	-2.38228357309046	-6.69066992462876	14.14708194848380
H	-2.28552667888347	-4.72940050270613	13.44143231546140
C	-1.47123958344780	-7.21391269694725	16.58259431252160
H	-0.69054779011264	-5.64946120280125	17.71423762013310
C	-1.54333148030802	-9.69616946660795	17.51878909437170
H	-0.82569411945430	-10.11629680072710	19.43026614395560
C	-2.51214943142590	-11.65203771986270	16.00460314021710
H	-2.55043152131019	-13.60869541302850	16.72190461866730
C	-3.42583521480106	-11.13465422019640	13.56792947169740
H	-4.15675579767268	-12.68799359261940	12.39061719431130
C	-2.47629886401149	11.33546083815530	7.60508173939396
C	-3.92697374427694	10.47232649562380	5.54743079954410
H	-4.04117919334563	8.43151349576007	5.13013869014405
C	-5.18653353323194	12.19243072649050	3.96801916441499

H	-6.27442052923965	11.45733443182530	2.35188826874172
C	-5.01036570729203	14.79649044964700	4.43628835615926
H	-5.98480912797090	16.15305081962090	3.18920486202629
C	-3.54865708375814	15.67926627692560	6.47276099431560
H	-3.37487921896905	17.72552309029630	6.82688656213469
C	-2.27217252552265	13.96379160103680	8.04006882431826
H	-1.07239637136406	14.67638171364000	9.58728929818629
C	10.01262317177070	5.85625053536516	7.53430220312363
C	8.72216220313173	6.97710120113222	5.49314312627407
H	6.74393222578119	6.45272249914337	5.09082521931437
C	9.95124694824823	8.71848604301301	3.91281588434617
H	8.89969044123658	9.53447268745623	2.31134889583371
C	12.48523234353790	9.35845755126309	4.36126773686832
H	13.46132060430440	10.71195099172020	3.11220423330141
C	13.79660309994320	8.23498457787432	6.38168938975564
H	15.79769028431280	8.70681202882608	6.72266690699143
C	12.57700307450200	6.48029867727079	7.95143794162976
H	13.64030489854490	5.55622484553194	9.48632839735344
C	10.86363648191650	1.17601166955798	10.07964728078460
C	11.89454693759530	-0.09247173661974	7.96745631542182
H	11.15326911749220	0.28260888000611	6.05259215310948
C	13.87894617812270	-1.81919630132463	8.28703422027882
H	14.66851081753340	-2.79388112148004	6.62509742156576
C	14.83800149744360	-2.32097499473945	10.71271538204060
H	16.39592400740850	-3.68258507022233	10.95840011791510
C	13.79074777228860	-1.09727610169473	12.81923672662880
H	14.51594564934120	-1.49525712234541	14.73106361774030
C	11.81149454085780	0.64618107190784	12.51167057443520
H	11.00873721863080	1.60133431575436	14.17728595913230
C	7.81144891104838	4.96668525116079	12.62491585974110
C	5.86304848808213	3.94006148231153	14.13061744440390
H	4.64916647727466	2.40581142618357	13.40391632673560
C	5.41853530185460	4.86406734802333	16.57683406297200
H	3.87344927896661	4.02541214775721	17.69381785040630
C	6.91236852821996	6.83505714324985	17.53866097558090
H	6.56536202690013	7.57110661037729	19.45761501688680
C	8.83701723988636	7.88599461622494	16.03986909848090
H	9.99961381176855	9.45064943858296	16.77774045588260
C	9.28690061954079	6.96493771608337	13.59260055876280

H	10.78457016446830	7.82406739830290	12.42990013830700
C	-11.55372837651370	1.17978951438218	7.57817513828934
C	-11.17460163157640	-0.46492929713143	5.52032170246591
H	-9.26795234712109	-1.20553575374436	5.11023695700813
C	-13.19415684550240	-1.12895082688422	3.93307548473731
H	-12.82548047943150	-2.39046981256789	2.31810194097894
C	-15.61799689866760	-0.15666693392284	4.39279001392124
H	-17.20453021739500	-0.66216350626299	3.13886512882954
C	-16.01291640402720	1.50305623648930	6.43062280905282
H	-17.90689338643730	2.29897941943604	6.78015270713665
C	-13.99218423614870	2.18451198510511	8.00618522606371
H	-14.30636756654420	3.54262759256972	9.55490439077490
C	-9.47943430610442	5.43954281001967	10.15161140427070
C	-9.51479464227167	7.09160577624125	8.05312365038066
H	-9.09369011973297	6.36820013038455	6.14119902819789
C	-10.10629474557460	9.65309100600135	8.38293909288184
H	-10.12746051738530	10.92170584265330	6.73221427741083
C	-10.64382070286300	10.60039641394310	10.80566648339620
H	-11.10559228248150	12.61661033459990	11.05857222844880
C	-10.56950299350080	8.97518998566219	12.89982936938090
H	-10.96629959412250	9.70706555467388	14.80952025148820
C	-9.98902768700917	6.40350754481634	12.58185108366190
H	-9.93991414012610	5.14363107786517	14.23817546771500
C	-9.24678310118753	0.55441301350292	12.66137891504460
C	-7.06873464713685	0.22238921472885	14.16592332851870
H	-5.18357571055845	0.75271440224316	13.44682724670100
C	-7.25734014598420	-0.80962091316830	16.60246916725430
H	-5.51657527089577	-1.05351063200547	17.72014131000680
C	-9.62638629153163	-1.53174061407371	17.55616362935700
H	-9.78065848622633	-2.34866688613044	19.46747828437440
C	-11.79956992431010	-1.23303376096478	16.05823483778900
H	-13.66189297762940	-1.81843014303074	16.78811424022660
C	-11.61685899644370	-0.20152955448548	13.62016333270760
H	-13.33205486504100	-0.00407924857193	12.45680990930440
Pd	-0.00862023815215	0.00809644970865	-5.41958666390627
Ag	-0.00406016319607	-0.00530192827805	-10.68350838534880
Ag	-0.45688150085933	4.71365150469436	-7.63682382786122
Ag	4.33842664404816	1.88775375226582	-7.61083626093670
Ag	3.13677947673596	-3.54100372224677	-7.60142499430734

Ag	-2.41010958026211	-4.06998875221582	-7.61095281916374
Ag	-4.63193822312788	1.04455897565197	-7.62366345764928
Ag	-3.24715070339556	3.40016866954882	-2.90368186840627
Ag	2.19603856114554	4.15488944129853	-2.90003888595507
Ag	4.60396518801192	-0.80080846601737	-2.88573854067464
Ag	0.64551134897133	-4.62859661879867	-2.88246490961571
Ag	-4.22368473621556	-2.01996694593081	-2.89439988911070
Cl	0.00997954224871	-0.04022995938954	-15.39465586244450
P	-0.78623669471530	8.99223632555103	-9.56400242142370
P	8.33049857059052	3.51016867182921	-9.49777157578789
P	5.94203756594980	-6.82506196279971	-9.46383602951814
P	-4.67501138478028	-7.72711195284914	-9.51785990285271
P	-8.80843411092783	2.06398191855454	-9.54607521452226
C	2.21866448636756	10.66880442848530	-10.17424613526120
C	3.78707345724239	11.20266908362830	-8.07966821084503
H	3.23491732675090	10.57370597391510	-6.16700449233904
C	6.04262834803072	12.55255538539110	-8.41284568299089
H	7.25076368786701	12.95602977054570	-6.76583521295360
C	6.77006742594516	13.36354035204290	-10.83521799096430
H	8.54645651721893	14.42259645082720	-11.09074174355420
C	5.23856670036791	12.80158295780760	-12.92561994505670
H	5.80560250972085	13.41145129572630	-14.83518359784560
C	2.97051397178433	11.45875575151000	-12.60394569880650
H	1.77900307611586	11.03132354662460	-14.25620046957900
C	-2.35126998612791	8.93541078537363	-12.68089043516240
C	-1.98617323233902	6.75833210143775	-14.17993438679620
H	-0.90222423958727	5.13124962026774	-13.45168177775310
C	-3.01051972468452	6.61559906626392	-16.62273608659000
H	-2.69745590648645	4.88445364122798	-17.73727915902510
C	-4.42274975099140	8.64547397169684	-17.58675412787860
H	-5.23433060783416	8.54022659050137	-19.50370272871820
C	-4.82048755636771	10.80610704882220	-16.09314257291690
H	-5.94908870273952	12.39496310442640	-16.83206054627660
C	-3.79679344700640	10.95511527120680	-13.64965142563520
H	-4.14348168420966	12.64801987708120	-12.48934202539190
C	8.68827566405248	-7.67597742944151	-7.48524757809421
C	9.35798782422389	-6.07347996072935	-5.46901737341161
H	8.25131705111752	-4.34479687980251	-5.09530770750126
C	11.39069288023450	-6.68384813976144	-3.87734728389708

H	11.84099228520170	-5.40750963135047	-2.29581897820452
C	12.77927368064700	-8.90457703061041	-4.29193890133399
H	14.36723316726840	-9.39648732528264	-3.03430900593522
C	12.11460863118090	-10.52784640973140	-6.28853330375102
H	13.17989504431890	-12.29127709516030	-6.60191066476971
C	10.07080115072620	-9.93051066397675	-7.86791376300996
H	9.52085054745349	-11.25023793995370	-9.38362873476431
C	4.50942041048992	-9.96316223355167	-10.01999827415170
C	3.62328093286744	-11.31876232944020	-7.89539456972238
H	3.75294102327926	-10.48434681278360	-5.98666679107475
C	2.59060002344162	-13.74017845127770	-8.19659145623946
H	1.90792452483548	-14.77968238706520	-6.52647746822498
C	2.40440191702840	-14.82525304365410	-10.61389626239970
H	1.58786904171877	-16.72867112470820	-10.84269078719090
C	3.24207480668294	-13.46801507007120	-12.73216763604320
H	3.08232398264078	-14.29443760491780	-14.63781886228200
C	4.29093791692981	-11.04561850608470	-12.44387065231060
H	4.94655058263982	-9.99805084412815	-14.11859823226280
C	7.17760822947646	-5.91034766039514	-12.59570940981600
C	5.61224828603114	-4.37798028601991	-14.11843078373000
H	3.78091929604427	-3.67918604255884	-13.40409581845690
C	6.36460274936473	-3.69855240014898	-16.57040400510830
H	5.10109623098533	-2.49137157980309	-17.70452742003790
C	8.69661693547312	-4.53606703872217	-17.52117897241260
H	9.29512850609167	-4.00326813987573	-19.44514460808510
C	10.27802494047420	-6.03797149695465	-16.00488792912610
H	12.12191088351060	-6.68208519209853	-16.73211446442320
C	9.53130259606047	-6.71965853284840	-13.55147509983860
H	10.80160031357140	-7.87388570738268	-12.37332441645150
C	-4.68922378599403	-10.60310255355650	-7.53877022166062
C	-2.96629157984920	-10.77602947480400	-5.51684773771247
H	-1.63948063407393	-9.21283875391907	-5.13522795884306
C	-2.95994905331867	-12.90183237734080	-3.92921060683027
H	-1.61501295972371	-12.96393703966330	-2.34063416792297
C	-4.67527649774245	-14.87922095974930	-4.35191190416561
H	-4.68652064667120	-16.54361016476400	-3.09699563258131
C	-6.41552194664633	-14.71622335172870	-6.35386111281882
H	-7.79008751240679	-16.24947790621580	-6.67447988412064
C	-6.43766390156154	-12.58461235117150	-7.93010097522690

H	-7.85392543419122	-12.44044877260750	-9.45126558489449
C	-8.09094926968796	-7.30968793381623	-10.12339284567120
C	-9.66788826655028	-6.80179204155791	-8.02882579779621
H	-8.84681395802775	-6.60696145345880	-6.11926908014013
C	-12.28643661522870	-6.56611057872344	-8.35716031741354
H	-13.49619581346450	-6.17168808456797	-6.70928836436270
C	-13.35730016805750	-6.80757317217926	-10.77569951149040
H	-15.41695914502290	-6.61755591187580	-11.02896357525980
C	-11.79301096807040	-7.26986238301919	-12.86647510345330
H	-12.61517668994830	-7.43997338095446	-14.77309652884240
C	-9.16867192142819	-7.52054858571976	-12.54930830763980
H	-7.95841989315557	-7.88942992302647	-14.20152399507270
C	-3.38589606696929	-8.64048589306624	-12.62779651139570
C	-2.38228357309046	-6.69066992462876	-14.14708194848380
H	-2.28552667888347	-4.72940050270613	-13.44143231546140
C	-1.47123958344780	-7.21391269694725	-16.58259431252160
H	-0.69054779011264	-5.64946120280125	-17.71423762013310
C	-1.54333148030802	-9.69616946660795	-17.51878909437170
H	-0.82569411945430	-10.11629680072710	-19.43026614395560
C	-2.51214943142590	-11.65203771986270	-16.00460314021710
H	-2.55043152131019	-13.60869541302850	-16.72190461866730
C	-3.42583521480106	-11.13465422019640	-13.56792947169740
H	-4.15675579767268	-12.68799359261940	-12.39061719431130
C	-2.47629886401149	11.33546083815530	-7.60508173939396
C	-3.92697374427694	10.47232649562380	-5.54743079954410
H	-4.04117919334563	8.43151349576007	-5.13013869014405
C	-5.18653353323194	12.19243072649050	-3.96801916441499
H	-6.27442052923965	11.45733443182530	-2.35188826874172
C	-5.01036570729203	14.79649044964700	-4.43628835615926
H	-5.98480912797090	16.15305081962090	-3.18920486202629
C	-3.54865708375814	15.67926627692560	-6.47276099431560
H	-3.37487921896905	17.72552309029630	-6.82688656213469
C	-2.27217252552265	13.96379160103680	-8.04006882431826
H	-1.07239637136406	14.67638171364000	-9.58728929818629
C	10.01262317177070	5.85625053536516	-7.53430220312363
C	8.72216220313173	6.97710120113222	-5.49314312627407
H	6.74393222578119	6.45272249914337	-5.09082521931437
C	9.95124694824823	8.71848604301301	-3.91281588434617
H	8.89969044123658	9.53447268745623	-2.31134889583371

C	12.48523234353790	9.35845755126309	-4.36126773686832
H	13.46132060430440	10.71195099172020	-3.11220423330141
C	13.79660309994320	8.23498457787432	-6.38168938975564
H	15.79769028431280	8.70681202882608	-6.72266690699143
C	12.57700307450200	6.48029867727079	-7.95143794162976
H	13.64030489854490	5.55622484553194	-9.48632839735344
C	10.86363648191650	1.17601166955798	-10.07964728078460
C	11.89454693759530	-0.09247173661974	-7.96745631542182
H	11.15326911749220	0.28260888000611	-6.05259215310948
C	13.87894617812270	-1.81919630132463	-8.28703422027882
H	14.66851081753340	-2.79388112148004	-6.62509742156576
C	14.83800149744360	-2.32097499473945	-10.71271538204060
H	16.39592400740850	-3.68258507022233	-10.95840011791510
C	13.79074777228860	-1.09727610169473	-12.81923672662880
H	14.51594564934120	-1.49525712234541	-14.73106361774030
C	11.81149454085780	0.64618107190784	-12.51167057443520
H	11.00873721863080	1.60133431575436	-14.17728595913230
C	7.81144891104838	4.96668525116079	-12.62491585974110
C	5.86304848808213	3.94006148231153	-14.13061744440390
H	4.64916647727466	2.40581142618357	-13.40391632673560
C	5.41853530185460	4.86406734802333	-16.57683406297200
H	3.87344927896661	4.02541214775721	-17.69381785040630
C	6.91236852821996	6.83505714324985	-17.53866097558090
H	6.56536202690013	7.57110661037729	-19.45761501688680
C	8.83701723988636	7.88599461622494	-16.03986909848090
H	9.99961381176855	9.45064943858296	-16.77774045588260
C	9.28690061954079	6.96493771608337	-13.59260055876280
H	10.78457016446830	7.82406739830290	-12.42990013830700
C	-11.55372837651370	1.17978951438218	-7.57817513828934
C	-11.17460163157640	-0.46492929713143	-5.52032170246591
H	-9.26795234712109	-1.20553575374436	-5.11023695700813
C	-13.19415684550240	-1.12895082688422	-3.93307548473731
H	-12.82548047943150	-2.39046981256789	-2.31810194097894
C	-15.61799689866760	-0.15666693392284	-4.39279001392124
H	-17.20453021739500	-0.66216350626299	-3.13886512882954
C	-16.01291640402720	1.50305623648930	-6.43062280905282
H	-17.90689338643730	2.29897941943604	-6.78015270713665
C	-13.99218423614870	2.18451198510511	-8.00618522606371
H	-14.30636756654420	3.54262759256972	-9.55490439077490

C	-9.47943430610442	5.43954281001967	-10.15161140427070
C	-9.51479464227167	7.09160577624125	-8.05312365038066
H	-9.09369011973297	6.36820013038455	-6.14119902819789
C	-10.10629474557460	9.65309100600135	-8.38293909288184
H	-10.12746051738530	10.92170584265330	-6.73221427741083
C	-10.64382070286300	10.60039641394310	-10.80566648339620
H	-11.10559228248150	12.61661033459990	-11.05857222844880
C	-10.56950299350080	8.97518998566219	-12.89982936938090
H	-10.96629959412250	9.70706555467388	-14.80952025148820
C	-9.98902768700917	6.40350754481634	-12.58185108366190
H	-9.93991414012610	5.14363107786517	-14.23817546771500
C	-9.24678310118753	0.55441301350292	-12.66137891504460
C	-7.06873464713685	0.22238921472885	-14.16592332851870
H	-5.18357571055845	0.75271440224316	-13.44682724670100
C	-7.25734014598420	-0.80962091316830	-16.60246916725430
H	-5.51657527089577	-1.05351063200547	-17.72014131000680
C	-9.62638629153163	-1.53174061407371	-17.55616362935700
H	-9.78065848622633	-2.34866688613044	-19.46747828437440
C	-11.79956992431010	-1.23303376096478	-16.05823483778900
H	-13.66189297762940	-1.81843014303074	-16.78811424022660
C	-11.61685899644370	-0.20152955448548	-13.62016333270760
H	-13.33205486504100	-0.00407924857193	-12.45680990930440
