# Supplementary material for: Key factors for connecting silver-based icosahedral superatoms by vertex sharing
Source: Commun Chem. 2023 Mar 28;6:57. doi: 10.1038/s42004-023-00854-0 (PMC10050180; doi:10.1038/s42004-023-00854-0)
Supplement: Supplementary file 10 — Supplementary Data 7 [file 42004_2023_854_MOESM10_ESM.pdf]

element	*x*	*y*	*z*
Pt	-4.10269686765402	0.71091963801962	3.51517586610415
Pt	4.02681276442390	-0.68539792196580	-3.55448265181842
Ag	-0.04308001808856	0.01371878934370	-0.02224421525424
Ag	-4.03122778362178	3.67591714026096	-0.95121880600608
Ag	0.79622560843488	-0.35942514709990	5.40688017029703
Ag	4.28816132094527	3.41008410602864	-0.09101692076505
Ag	-0.38005754499457	4.51822620500639	3.12728718264207
Ag	-2.14951706988351	-4.21531521902908	2.79040964910834
Ag	-5.16217097453410	-1.72384915309183	-1.12687439313268
Ag	0.67580056358634	3.39597217560669	-4.30851679224682
Ag	-3.70220752060792	-2.15508264549469	7.87205767486614
Ag	1.66174960794098	-5.00900987998156	-1.48890319034856
Ag	4.90532849116048	-1.78125265828655	1.61421553363437
Ag	-2.81719298843517	3.36384871818542	7.84258050292598
Ag	6.25725667455346	3.78002450577748	-5.14946552841860
Ag	-0.96441417872508	-1.80835030844444	-5.13134711841207
Ag	-7.41116651574959	-3.34391597971308	3.83851779768222
Ag	2.90962010746282	0.67098017237399	-8.48054255805969
Ag	6.96062591883255	-4.92767298135427	-2.68844303969855
Ag	8.79412035414426	0.22921176439701	-1.62465752454609
Ag	-5.89699725409072	5.62996961023015	3.76624489292622
Ag	-8.78766586793675	1.50469942850841	1.36454393861358
Ag	3.30994244771875	-4.66858457784088	-6.90619499950502
Ag	-8.04990658039411	1.39031394609197	6.95485552498540
Ag	7.94768157538121	-1.34466521520097	-7.02084671778496
Br	3.72128421067347	7.41334046398500	2.95939196322061
Br	5.42425910054403	-1.96719111514433	6.64151382561211
Br	-5.64663043607453	-3.52271493205926	-5.81331594796005
Br	-3.07120421397325	6.70906763767653	-4.87519584560345
Br	-0.56224775885701	-8.69549042519325	1.10082106086563
Br	-11.66539557228990	2.08339487443936	10.16831615592680
Br	11.46321461055230	-1.95513598193632	-10.36351895917530
P	13.00692121549340	0.91873960586928	0.29319498912011
P	-7.47908542694515	10.03765032550960	3.60901842193950
P	-12.94315336630130	2.08136901762829	-0.68046365694823
P	8.21607509933763	7.95216170265409	-6.07615446522555
P	-9.95085255474696	-7.29095197906935	4.07949119852125
P	-1.50699520821904	5.90568369539524	11.54581686147860

P	1.79953650138915	2.01242551842871	-12.81967937454100
P	-3.02223094334493	-4.63778420220688	11.78168162028560
P	2.41481786855867	-8.14867401379978	-9.92240874698457
P	9.37275385633418	-8.84003070309694	-1.82096558359859
C	15.33246151735410	2.08397529478445	-2.03534288540368
C	3.30013571409245	0.12219409348423	-15.33556360456880
C	-15.08562805965280	4.22017685372641	1.05005600228509
C	-16.94499592102790	5.72476779573169	-0.12564180393923
H	-17.09064219474750	5.77901699640141	-2.20156767552142
C	-18.61020527641670	7.17429665807270	1.34704376412872
H	-20.05761582665280	8.34383607585187	0.40845493996189
C	-18.43393738307400	7.14368188809160	3.99681602433567
H	-19.74941066710050	8.28351997584200	5.14319454915994
C	-16.56623314680740	5.67323002935157	5.17841721989292
H	-16.37210372698280	5.63685903616799	7.25249324429610
C	-14.89402046024360	4.22693527525905	3.71207773972568
H	-13.42572263805070	3.10044569488844	4.67867537243035
C	8.27326753184254	-11.74161104258050	-3.43106490559108
C	3.49191361946148	10.27900193721150	-5.51237766194675
H	2.98208418857226	8.53991564695158	-4.47842149948286
C	-14.46953452105010	-8.92143870460542	6.61474250970326
H	-13.73105604418650	-10.86585269322360	6.50206596994268
C	-1.62801349288067	2.08485907927399	-13.47636765974160
C	1.39170460438400	-2.38644032692353	13.62495513194030
H	2.11539791408652	-2.69154362356672	11.69044008388340
C	10.93632734157520	4.83283141249665	3.22690371761010
H	9.36035699162929	4.77178020598821	1.86078755286557
C	14.38137542429520	-5.16691817082630	4.94954598100131
H	13.33811595358260	-6.14377870697380	6.46372984791732
C	-1.12119898810184	-8.61930992634642	8.82432062444985
H	-2.06160999162151	-7.64132134049335	7.23933271278918
C	-13.04101559869800	-6.88304619475997	5.64210953164049
C	13.25359042905010	-3.12001900293653	3.69971785950825
H	11.34340356756670	-2.49490753126372	4.26323131648398
C	-10.57144970592510	-8.77810583851872	0.97307616790323
C	10.36705470875870	7.91676648700991	-8.81966975268893
C	-8.84429887172834	-8.30429570678310	-1.00013007873088
H	-7.26705418116480	-6.96736419926367	-0.71625383940265
C	2.32775403644605	14.46102770893220	-7.01992281347271

H	0.93950777098360	16.00862670382810	-7.16646507016549
C	-6.00453422264833	-5.46127569949454	13.39552438543600
C	14.96661506060200	3.24041899246610	4.76902921685474
H	16.56531029567340	1.91164023140582	4.62592162446329
C	14.56135305850340	-1.84914576546555	1.74788326201887
C	1.74313983349498	7.25215382072903	-12.10242012540030
H	0.44328699087670	6.81967134779463	-10.52742267130830
C	8.94007412067676	-11.38114364661110	6.61828535434324
H	8.77536606944576	-11.97895559424970	8.60800856026378
C	-12.59150631585070	-10.46789478563600	0.55232992020917
H	-13.98521676836920	-10.83483524442940	2.05374846808831
C	4.71011471303205	14.69455961670690	-8.17599945075250
H	5.19390742170056	16.42190783814790	-9.23716182902954
C	-9.97760913983946	9.20194938305031	8.16548686117971
H	-9.94671810548380	7.19744112680758	7.58378278843372
C	-16.80638292038260	-8.47469509879718	7.78500433682308
H	-17.90524475294400	-10.07761732994570	8.53735349135886
C	2.37900866625719	9.77150516173843	-12.61595667203150
H	1.58549087980709	11.28939134632820	-11.43277196995480
C	14.73258658588790	-9.76406112008416	-1.54543514759011
H	14.39006144662670	-10.87175999688770	0.18330277881621
C	-13.73420237134890	-2.84966818967978	-2.40570631058531
H	-11.82697707334220	-2.64743867310865	-3.23069867707695
C	-2.65882655567038	3.50309130395408	-15.49169739408940
H	-1.40868096588467	4.61458546147752	-16.73425091033410
C	4.05093453110035	10.35153620867050	-14.59752768209770
H	4.56100227476729	12.33454309439600	-14.98380150440450
C	0.27613470589354	-10.82276165246330	8.34339996594481
H	0.40273926858704	-11.53196147939090	6.38968107881410
C	-5.10016120721687	12.40900308294630	2.64768984923276
C	-17.49357805756080	-5.40221948021870	-1.70093593094680
H	-18.53694129800660	-7.18593611666986	-1.96666877111322
C	-10.01923624380230	10.68739269808910	1.29773684905259
C	6.03761593460929	3.89432368755132	11.38126270089650
H	7.15985330493583	2.45249793258292	10.38082681203840
C	8.16854390424057	-8.94813137445960	5.89608758169095
H	7.37283902227251	-7.61377151436759	7.28344569549195
C	13.01098647522680	3.18689619628102	2.95005789023609
C	-14.81740110246270	-0.84659439381435	-1.01015045900514

C	-1.27134410319637	-7.61954371152103	11.28887719681740
C	1.91899564486240	5.81397672613874	12.21426660960150
C	8.91865504679122	-15.46407729346710	-6.07040929641928
H	10.16561582059180	-16.60754976574850	-7.28669493234947
C	2.97256929625032	-1.29547877150350	15.45047066917280
H	4.91019743063252	-0.74133283154515	14.92666538050200
C	-6.27996567010108	-7.59204353247917	14.97617768684340
H	-4.68081505936173	-8.89492782473765	15.26555898390660
C	6.48607585005188	12.73393000540420	-7.98967000255737
H	8.33704972670741	12.93786332302040	-8.91785712091051
C	4.43831704263746	5.87047484285198	-15.57272719561490
H	5.25045914823162	4.35099911379745	-16.74047038180110
C	5.74290585968680	-0.82880814750731	-14.83962900435100
H	6.63553424421676	-0.56274147763625	-12.97050859879550
C	-8.46623493685695	-9.92677503258901	5.83860447128858
C	-2.52976402049494	11.71540175954120	2.63231474802336
H	-1.96289037249397	9.79334463127837	3.21319889251727
C	5.31922528343487	-9.74380544428530	-11.00993993986200
C	5.45965159322342	-12.33914500101110	-11.60087495552390
H	3.80228704013055	-13.56819814710150	-11.32871408532830
C	7.72521631059110	-13.37509513806900	-12.51575919356360
H	7.81557447992790	-15.40747605365860	-12.96816023472360
C	9.86815362592106	-11.84079585534370	-12.84146347773390
H	11.64320935257270	-12.66622648915090	-13.55734499896350
C	9.74971888677735	-9.26102332951448	-12.22831782728720
H	11.41296987754950	-8.02327640736388	-12.43132624960530
C	7.49057834725848	-8.21995799313436	-11.30874901386450
H	7.45117453445202	-6.19485746894452	-10.80765420667840
C	3.40937064636921	3.95935548111590	11.02003599337950
H	2.52034558577274	2.56941957882477	9.74279274580200
C	16.98473028268450	-2.69361143119603	1.03485069078525
H	18.01147338518140	-1.74018271362169	-0.50385282117506
C	0.78260354962374	-7.26138070639830	-12.88354416452370
C	1.76813383698043	-7.78908392222579	-15.30081565023420
H	3.63617570919097	-8.68664269516676	-15.49294733046970
C	0.36328498719847	-7.18303577825180	-17.47121701435220
H	1.14984344346881	-7.60818980151897	-19.35298438453270
C	-2.02151860017694	-6.04452828012916	-17.24856268301400
H	-3.12119390252743	-5.57521397561638	-18.95526859445030

C	-2.99600099360020	-5.48303578699477	-14.84220326156190
H	-4.85239676983496	-4.55910159081446	-14.64919560585160
C	-1.60197364011718	-6.07839565537842	-12.66843557813960
H	-2.39051519627902	-5.63977460461508	-10.78607378832130
C	14.85317067893330	4.94282832001132	6.79862140861483
H	16.38707226107910	4.96625201399228	8.20979409060042
C	-16.30738004432480	-3.95774218116008	7.09804412785465
H	-16.97569574765040	-1.99938304645127	7.32889999324893
C	10.81411392074650	6.51789677045026	5.27179089621098
H	9.14371378200569	7.74567569858872	5.47013459274084
C	-9.48210671398210	10.25877061444550	-1.28302228978129
H	-7.62437201230441	9.50162271451228	-1.86058662727435
C	-5.89562522987124	0.82522481116083	-12.23264527703800
H	-7.13728308072906	-0.19089924897310	-10.90366282116480
C	7.19832775018965	5.68146132845204	12.96051597259880
H	9.26434170343083	5.64453771969169	13.23457402627070
C	10.14750248664040	9.30959545675780	-3.49231014103324
C	-0.67030026299232	13.42612141251250	1.81895993057742
H	1.32018992858286	12.81102526681600	1.78748770320958
C	-2.05343985685816	9.35170680934698	11.32914561474110
C	-11.29524148852950	10.81733687115130	-3.13271388331561
H	-10.85112184230650	10.47523180181730	-5.13846763896424
C	-0.82652290333578	10.67951681745920	9.36347465271099
H	0.36895166631240	9.65443410583626	7.99291531612390
C	-6.10451953608761	-13.87399673392840	8.55357932744423
H	-5.18631608283293	-15.41397166570430	9.61405973814431
C	10.20896689797930	5.84575305452517	-10.49043456840760
H	8.86083870684968	4.30058063208271	-10.10637910554530
C	5.75329859953305	-12.51367414175140	-3.00139424373390
H	4.49381003129948	-11.36223922425670	-1.79861005444504
C	1.71556716141226	12.24526859593690	-5.69544089235427
H	-0.15307521641210	12.00696113930300	-4.80703519845277
C	-6.90156143129716	2.20678911355998	-14.26206372941780
H	-8.96369468476019	2.26847438335130	-14.56086828033970
C	12.70862126664320	-8.56167442281657	-2.79385075023040
C	0.25345288455181	-10.61828423954790	-8.72115165219267
C	-0.32560629825539	-10.67122687802700	-6.12369116461697
H	0.53076087580650	-9.27432531428334	-4.83285924082820
C	-2.04157809649358	-12.45020981961920	-5.15662199968827

H	-2.48840789233423	-12.41536697695920	-3.12226570273136
C	-3.18807093614401	-14.20416955228490	-6.78315379326040
H	-4.54641947094609	-15.59501756344250	-6.03141578367942
C	-2.63513706270367	-14.15722547824380	-9.38255139486879
H	-3.55598791285907	-15.51029756829000	-10.67293403078830
C	-0.94102259513072	-12.36378761554230	-10.35375791588440
H	-0.58044562329268	-12.29299707946610	-12.40485970800460
C	3.10728583938159	7.62912885323833	13.77393585580490
H	1.98370091093699	9.14558533700778	14.65673225758640
C	-8.60324414588680	-8.07909386516477	16.16320503445200
H	-8.80259689694177	-9.75336064753464	17.38902315927340
C	1.52972817068578	-12.05529578347740	10.32933790201630
H	2.64589794023210	-13.77434158564370	9.95133349243523
C	-3.85656923402448	13.32073714786110	12.82492878881850
H	-5.05994274737760	14.34681945408640	14.18118463903260
C	-9.10395565649586	-9.52195983593142	-3.34634292634724
H	-7.73353806198934	-9.10559261430394	-4.85852321471502
C	8.36436273277381	-8.19947664805254	3.35729808275871
H	7.71642335848269	-6.29508895280363	2.80163612337471
C	5.89719143184091	10.51344045305590	-6.63572642468875
C	2.19777889019660	-0.30057149216594	-17.72465283611680
H	0.27409737679590	0.38507296080087	-18.13005709862210
C	5.08577173799430	8.39939920101726	-16.06361744860110
H	6.41193219558434	8.83818180791966	-17.60964893800100
C	9.04515980921390	10.95021237008200	-1.69934428153438
H	7.04094128343113	11.48614814325630	-1.86214171423836
C	-13.97581418403230	-4.40160811516063	5.91185277451047
H	-12.84364503844990	-2.78718722080208	5.23082439685285
C	-5.28069089457556	3.54512075534329	-15.88867642792400
H	-6.06599202002975	4.65571821112978	-17.46775917223190
C	12.17740280219240	9.82640376890818	-9.28858943115581
H	12.39327462880200	11.41602467109340	-7.95997800652992
C	-15.07578466797360	-5.10518253450438	-2.76089048048014
H	-14.20670128005680	-6.65330935166476	-3.84793631087422
C	-18.56346836330420	-3.43209777988923	-0.28502554539195
H	-20.45209387906970	-3.65819258014872	0.56594917028212
C	-7.46381323715726	-11.99375618790260	9.84087779530602
H	-7.62340005894266	-12.04351678863300	11.91589264773190
C	17.43167195084590	3.59650962314923	-1.38579909579567

H	17.73732861831710	4.16507287773900	0.59443986124727
C	-7.06981128826509	-11.80547433897830	4.55795950144658
H	-6.89479428376412	-11.74932144181720	2.48536281679942
C	2.08157096732970	-0.89054821995094	17.92017373523390
H	3.32778984823227	-0.02983434484713	19.35112286182740
C	16.81397950502610	-5.97877431892899	4.25667598125082
H	17.69784106096050	-7.58786219180062	5.24226603249633
C	11.79916427009300	5.69247854337093	-12.60927811050990
H	11.67140642957770	4.02898839344895	-13.85444526318040
C	2.75248637265321	5.28087277417145	-13.59568187987970
C	18.10276428281350	-4.74872429210455	2.29180166749804
H	20.00146001834530	-5.38867892258506	1.72069866778175
C	10.49044324428730	11.91466442827340	0.30402315991948
H	9.59518249579321	13.18808820267500	1.68862922435569
C	-1.09498984980574	13.30780659189580	9.15699911661963
H	-0.13031880306062	14.31891834288170	7.61315171642273
C	-8.62263095241813	-10.01876024036580	8.50250552312355
H	-9.67076423984008	-8.55381094489785	9.55013209856091
C	18.73377132407250	3.72572528776589	-5.80311707193566
H	20.05659917480380	4.38347168919025	-7.27291617114007
C	-3.17291066035359	4.98891495529007	14.46811687039250
C	-5.71036154902407	4.20074740094855	14.21615346532130
H	-6.60192820641014	4.05205222748225	12.33410119771930
C	-7.14401757951819	3.57792745524808	16.35888583685250
H	-9.11824153305437	2.96495232850750	16.10069630761250
C	-6.05082483552759	3.71968034772542	18.77344500428950
H	-7.17031668778276	3.22374698581220	20.46005507091780
C	-3.51802404113635	4.47443838094401	19.03426836102880
H	-2.64314056713613	4.56681019345642	20.92372315672650
C	-2.07886850579604	5.10421250235004	16.89534070587270
H	-0.08776769199679	5.66872202870979	17.12188026398390
C	-10.49586234771400	4.23476740368685	-4.87257510444410
H	-8.85698222307867	4.39973184146613	-3.59200273717892
C	-10.25493403944740	5.01732476093274	-7.39692119441503
H	-8.43143213133548	5.78446738489182	-8.05086549542660
C	-12.31043228112170	4.77585529415255	-9.05726843468270
H	-12.12293055070220	5.38082351878015	-11.04326797031900
C	-14.59523364517100	3.72537452781640	-8.19612843643926
H	-16.20403311785230	3.50408076723771	-9.50206940162768

C	-14.82792125320750	2.91862723369660	-5.68050575403166
H	-16.60043386583020	2.02986088917351	-5.04083333892143
C	-12.78085645557150	3.19288789715597	-3.98629962456976
C	-13.66650112502910	11.78677289134330	-2.43125817450900
H	-15.08897460925750	12.22359987665400	-3.88999437913870
C	-3.58867521228136	10.68826036431200	13.04695442058470
H	-4.57483093529859	9.67458729348791	14.57415690419100
C	-2.60348969674666	14.63635208079310	10.89326026761300
H	-2.81394166597810	16.70284274946110	10.72638815098870
C	1.39421074386767	-11.07258362518200	12.79492072100450
H	2.39550476522833	-12.02103470955690	14.35760506265090
C	17.20264792235970	-9.53170961487596	-2.48858160732873
H	18.77361341213550	-10.47606673583530	-1.49654628956134
C	-5.89784483144612	-13.76242426610310	5.91162225783073
H	-4.81305476132187	-15.21148464869840	4.88001085616620
C	10.04723146580280	-12.33341149975740	2.25170633890777
H	10.70828154105790	-13.69863272572490	0.82329751371756
C	5.99054693778568	-2.52196675744420	-19.10406825728630
H	7.03951847230312	-3.54929848106101	-20.58343253601920
C	-1.10082672092060	-3.10589998579014	14.26171576923560
C	12.70993722722620	8.61579149154014	-3.20978101688693
H	13.60584539285680	7.30750417614509	-4.56124766546399
C	17.67760177342840	-8.09650386654008	-4.67205949982871
H	19.62232966141700	-7.91876348950493	-5.40052118673545
C	-0.40534456297216	-1.56403031300299	18.54857672282510
H	-1.12674579860450	-1.22914102945373	20.47396243066640
C	-8.84683347387896	13.60954353243130	7.48218794553282
H	-7.91833543340249	15.09047622118300	6.35164408316563
C	-10.40715127349450	-4.32501365142072	14.21942584453760
H	-11.99963755508580	-3.02599466594426	13.87673841087530
C	9.31263474710391	-9.87238279740789	1.51494611770088
C	-1.36689844133910	15.85847670112900	1.02125969671790
H	0.08693400736592	17.20086538031360	0.36721693212009
C	-10.02889606928780	14.25242560375310	9.76915309065149
H	-10.02783568687810	16.23775000909590	10.40364981989890
C	-8.83085132952331	11.07656385476640	6.65135911553780
C	-8.08718584762934	-3.83736322532353	13.02678918147340
H	-7.93260141280564	-2.17019321446707	11.77923587471940
C	5.72957982973364	7.54556056775449	14.15790793966250

H	6.63796665482497	8.97057091949683	15.37761477797000
C	14.14818956348550	9.60516398400471	-1.21275349397287
H	16.14853427209400	9.05611978971524	-1.03322567308104
C	-10.66911407772760	-6.44977327693195	15.78709049928630
H	-12.49194770522700	-6.84839126399708	16.71513070321520
C	13.75271374463910	9.67855447559741	-11.41659596627400
H	15.15693410115220	11.17838263512100	-11.76572226429190
C	13.56265725818230	7.61588348380673	-13.08219443028330
H	14.82056795951050	7.49625383671867	-14.73943642889720
C	-12.85078506168790	-11.68118678007390	-1.79139965573330
H	-14.43717980067230	-12.99790114517450	-2.09834410955624
C	12.77647263298190	6.58064313388783	7.05469948149720
H	12.67475299605370	7.89157970134489	8.67232112471587
C	-14.22138414959080	12.17633466403950	0.13233921365818
H	-16.08410972123650	12.91500816780090	0.70163900983893
C	14.96136498874460	1.40109955532563	-4.58714125003238
H	13.32275296040510	0.23779517544243	-5.15058707542378
C	-5.78994810726586	14.84737338969650	1.80153753669502
H	-7.79875997705343	15.39178370571000	1.70965300900265
C	6.42674992762250	-16.24437946084010	-5.60611682542733
H	5.70622424156102	-18.00770931776400	-6.45100656892659
C	-3.27063351089043	0.76396851433197	-11.84861086028480
H	-2.50086009271478	-0.29351577397509	-10.22269750305530
C	9.88172729150229	-13.07004797270570	4.79392393776646
H	10.46171635292260	-14.99337773658870	5.34894291921079
C	7.08545961282869	-2.13463808999504	-16.71777923787520
H	8.99062673657078	-2.85018230048261	-16.27208723688600
C	-17.73080731718560	-5.99463864765951	8.02381498166760
H	-19.55731372777840	-5.65082324273523	8.96651165054785
C	-12.41057150128090	11.62882661592060	1.99595027011825
H	-12.86424806389490	11.94588786489980	4.00241747655653
C	-3.92928489640326	16.56502405752120	1.01428593422826
H	-4.49050748715781	18.46206732038080	0.35869193108765
C	-17.23724231045010	-1.15651122442426	0.05844336774633
H	-18.09697466759720	0.38491576590203	1.16213650661641
C	13.20410436077710	-7.09547667392552	-4.96877249927317
H	11.65842098918520	-6.09440416273390	-5.95132868731088
C	13.04953420177160	11.26019468844540	0.54580129207706
H	14.18347972477850	12.02033792406920	2.11927488957624

C	-1.99764398003184	-2.66601489759801	16.73090554558060
H	-3.94589032100666	-3.18793282546447	17.24478552666780
C	9.84469778623501	-13.22086846588980	-4.99188503346203
H	11.80476875246300	-12.62754397790280	-5.36441979757748
C	3.54370862587206	-1.61487846853867	-19.59605891532350
H	2.66336087673136	-1.93790449336739	-21.45760057583360
C	-11.10344458341970	-11.22013618447140	-3.74099519239393
H	-11.31178563376050	-12.17852571566440	-5.57996383902728
C	19.12037278367970	4.41119964888172	-3.26415450595659
H	20.74926151677420	5.60100461042739	-2.73939907505745
C	-11.19595390295150	12.38103831551600	11.24962769665010
H	-12.11872299200210	12.89848583915790	13.04549288064720
C	0.01864387923018	-8.85651197900489	13.27476862104540
H	-0.00991850507741	-8.05606178808809	15.19822582666180
C	4.84312381176351	-14.75777217931490	-4.07732307475307
H	2.87253042462818	-15.33420898654260	-3.73016195992878
C	15.67390938788460	-6.86930594491761	-5.90752546294500
H	16.00015716358460	-5.70091367352911	-7.60093407699980
C	16.65450656463150	2.21372827241279	-6.46204585034967
H	16.29904062849420	1.66140722830446	-8.43912794885032
C	-11.16120194910630	9.85119864390423	10.44872522644060
H	-12.03094982282210	8.34136953489359	11.59081504543640
