# Supplementary material for: Key factors for connecting silver-based icosahedral superatoms by vertex sharing
Source: Commun Chem. 2023 Mar 28;6:57. doi: 10.1038/s42004-023-00854-0 (PMC10050180; doi:10.1038/s42004-023-00854-0)
Supplement: Supplementary file 11 — Supplementary Data 8 [file 42004_2023_854_MOESM11_ESM.pdf]

element	*x*	*y*	*z*
Ag	0.03941033319690	-0.01268585404113	0.01870430356657
Pd	-3.99074401133307	0.66838748239673	3.53982448761313
Pd	4.05836646785310	-0.69050007969068	-3.50676712124820
Ag	-0.83121861871381	0.33311113319428	-5.39750938493084
Ag	4.04585350140843	-3.63720727490736	0.94815126448362
Ag	0.38956172307590	-4.51941680076588	-3.10580899668999
Ag	5.13500353001790	1.76357381865590	1.10078130010103
Ag	-0.67471520503847	-3.42918761730721	4.26353798534351
Ag	-2.88270039380589	-0.71457267128263	8.44097209782836
Ag	2.09172842349256	4.21224036222696	-2.81416747388613
Ag	2.78586866670237	-3.34296385129837	-7.82200634196126
Ag	3.64549870967839	2.15684987975458	-7.85507042434238
Ag	7.35120411979274	3.35818514920257	-3.85273899739857
Ag	-4.29251421834688	-3.38682285934787	0.06361121096769
Ag	-4.88704909388073	1.81506462619570	-1.59702460328347
Ag	-6.22827135676606	-3.78058924587633	5.12047957574121
Ag	0.97809908798355	1.75306040087001	5.13028596481924
Ag	-6.91833970982023	4.90112718293852	2.71734586288829
Ag	7.98568819606569	-1.35314488046638	-6.94862701283578
Ag	-1.62942884151117	4.99882729988548	1.52335219978915
Ag	-8.75118500664969	-0.22826134820336	1.63030165317001
Ag	5.87884380036834	-5.58422836592342	-3.77611604253615
Ag	-3.27689582232001	4.62037243079794	6.90898495046387
Ag	-7.89320498790610	1.31228103137192	6.99927552847210
Ag	8.74490252147287	-1.45984943349878	-1.39082622965443
Br	-3.72823709103899	-7.41990301589837	-2.97159304492533
Br	3.09834264969891	-6.74886931554682	4.83192907735654
Br	5.64218008730271	3.54623310456322	5.80882085596330
Br	0.54523863441630	8.71740800361924	-1.10338867284995
Br	-5.46646857833400	1.97111303748534	-6.63729049591287
Br	-11.40930577214630	1.91962629372594	10.36263719250220
Br	11.60798076116730	-2.04537627670190	-10.17570216276800
P	-1.76360579285238	-2.08920605146366	12.78915135820830
P	7.48028158923422	-10.00898432533370	-3.60537767826835
P	9.87805751739075	7.34181119149902	-4.10269435819325
P	-12.99030251954080	-0.89652917140520	-0.28563039163185
P	2.94511680681731	4.64491976275888	-11.78363487725740
P	1.46535269232263	-5.90723656476088	-11.53297373909480

P	-2.34575832291961	8.09192339584895	9.96102207895698
P	12.92194170485280	-2.02916504433524	0.65677345615332
P	-8.20121914491123	-7.98016959720650	6.01477787258038
P	-9.32301875399540	8.84810006760139	1.87671170060573
C	-13.28460094901180	3.16454826922909	-3.66215777702557
H	-11.39459871405560	2.52630625666430	-4.27652587494420
C	2.00657817396054	-9.35313505073351	-11.30724002066310
C	1.66490458072215	-2.16718725273948	13.43838256561410
C	-1.46407843289886	2.39659192602598	-13.64296761520390
H	-2.20116603116490	2.71712824805432	-11.71591545966670
C	5.10658731117840	-12.38291383833800	-2.63830406029438
C	-8.37716575016218	8.20694714502388	-3.31183616444209
H	-7.75527264498852	6.29055769354538	-2.76750324479798
C	-14.55820030486090	1.88852863172902	-1.69094048771215
C	13.69588977207490	2.90571755666175	2.37722762832430
H	11.78997082440950	2.69733525604702	3.20401927170472
C	12.96718762988630	6.94175284486786	-5.66944553979532
C	8.38209966833851	9.97301477245634	-5.85771919608341
C	-0.19108523797561	10.56557289656940	8.75668237387752
C	0.34624906666508	10.64686741740510	6.15100739331305
H	-0.54092518997694	9.27203710278114	4.85717493460729
C	2.05930308393596	12.42513247094430	5.17786725546430
H	2.47455684056567	12.41053811982930	3.13656494596279
C	3.24416903931790	14.15125689021990	6.80656729825444
H	4.60110685673136	15.54072127816680	6.04974601021297
C	2.73147741794968	14.07762527905540	9.41358751604708
H	3.68129881910980	15.40949755136130	10.70503878457710
C	1.04078700400802	12.28402280198450	10.39044130332160
H	0.71317576823208	12.19079422711370	12.44612348195880
C	10.50198646496660	8.82897276757038	-0.99699132017667
C	-1.62731833242792	12.04784675659500	-10.32294238714680
H	-2.74672623753352	13.76432104227120	-9.94277231218840
C	10.02813618697230	-10.65610677711820	-1.30237641955037
C	15.02897142847060	5.16684806937560	2.72886816899455
H	14.15458352939280	6.71269260667236	3.81488074585415
C	-10.82243113221130	-6.43962130933312	-5.33626686698125
H	-9.15496278660240	-7.66811708442570	-5.55388508802058
C	1.71095069695473	6.03502401182533	12.65849950622710
H	2.50380225849605	5.64624247144731	10.76692634617680

C	-10.12067194546200	-9.32579922331624	3.41615872326335
C	2.53448186155699	-11.69587437046490	-2.62953358984697
H	1.96333121267973	-9.77755955114346	-3.21893510728081
C	-7.23723933656301	-5.65876414026975	-12.95899158106520
H	-9.30259826673436	-5.61432223588532	-13.23699297409410
C	14.38732957783590	8.98215079567810	-6.65010277768250
H	13.64264485832860	10.92438145641250	-6.54101362045309
C	8.82326762272341	-11.04841885504850	-6.65132817988586
C	-13.00600877374050	-3.13092428697968	-2.97003029615378
C	-0.36781248207329	10.81784827584790	-8.33914247357434
H	-0.49363918099035	11.52539131193060	-6.38475939130271
C	3.55649121577743	-10.69314977936130	-13.00926263387010
H	4.55319930731819	-9.68303861799690	-14.53193720070230
C	6.97601145498864	11.84195935857050	-4.57302081971934
H	6.80203235126444	11.78093556478430	-2.50052671851457
C	1.03413513852641	3.10748203770728	-14.26760657713230
C	-4.43455886281588	-5.95414682461177	15.49921219454980
H	-5.26269788857416	-4.43739930607675	16.65923960371010
C	-1.95960962192875	-5.80903834761791	-12.20443003324830
C	-14.96918961984220	-3.16264946890163	-4.78129260955668
H	-16.56602892586900	-1.83439759901929	-4.61603083508206
C	16.72378264610400	8.53993240443408	-7.82293062222307
H	17.81626726640050	10.14453152760000	-8.58099050063167
C	-2.72073820264160	-5.36007920116395	13.54761647681960
C	-10.36411895974270	-7.95475708581801	8.74905851181403
C	3.13417186741905	-5.00029918629730	-14.45676524332460
C	14.78614381832590	0.90506294868638	0.98352899863899
C	-6.07183735814427	-3.86925167084498	-11.38588863498210
H	-7.18951746575408	-2.41872043276134	-10.39295553865550
C	-3.25452557095290	-0.21161768018380	15.32026787666150
C	-16.95581906996540	2.74979104701243	-0.91323611300625
H	-17.95597989091710	1.79172025296519	0.64006472108688
C	-10.93428162164340	-4.77608869389234	-3.27328384525737
H	-9.35269135586008	-4.73223418339751	-1.91299843414136
C	5.92701103897633	-0.87732363197785	12.20655847567270
H	7.16409272001726	0.16020433146490	10.88982449203740
C	3.30143593443492	-0.81909908886838	11.82684727073410
H	2.52634373058760	0.25934657293638	10.21716697743510
C	12.76804549971600	-3.14341189020893	3.96192978569995

C	10.01824503844170	-14.22547310178830	-9.76896647835929
H	10.01953528741990	-16.21156544886130	-10.40107022210940
C	-10.21737188969940	-5.88567095054363	10.42323871721410
H	-8.86926494189634	-4.33806339211977	10.04858771086430
C	-9.00452688711703	-10.94396206478010	1.61123726772543
H	-6.99840114500188	-11.47204291065440	1.77536281009432
C	-0.68999557525410	7.17945314595930	12.90098230713950
C	-1.69174140017237	-7.32811823214019	12.06320449787140
H	-0.37121271036282	-6.89256401087525	10.50640121617060
C	-9.27794760637242	9.89072850936219	-1.45557346324187
C	8.84124401048146	-13.58225409877860	-7.47952674553710
H	7.91882041637775	-15.06348025657770	-6.34434150282081
C	-12.65447501971550	8.59254793980250	2.87144124539229
C	-14.66942504161300	9.85805361234750	1.67126239441874
H	-14.32363195893400	11.00252766365980	-0.03264124174265
C	-17.13419855205630	9.64398448055825	2.63158384516652
H	-18.69792266827210	10.63845935315500	1.67787920075304
C	-17.61350827798890	8.16477033012329	4.78490540955562
H	-19.55426664469110	8.00250631001213	5.52747557377426
C	-15.61973214983600	6.87595265790664	5.97236007200049
H	-15.94918490481830	5.67509701325093	7.64231085599618
C	-13.15494972998120	7.08348214215421	5.01529596328083
H	-11.61698775049880	6.03590631781575	5.96102665313013
C	-5.68922558301065	0.76275736153824	14.83062577119580
H	-6.58192630670871	0.52053953133753	12.95833236387750
C	17.65616475748900	6.06236845835525	-8.05654104886989
H	19.48224919300380	5.72205940277816	-9.00129486815664
C	5.92598436787532	5.48236155150889	-13.39313210413690
C	-14.42174918112760	5.23337440527883	-4.86663391734112
H	-13.40517820054350	6.21379050688399	-6.39659948070177
C	15.06972806340210	-4.16203704609488	-1.07417381273087
C	-8.18945952160148	11.73153419011810	3.49439833587215
C	-15.29975230760510	-2.09756086720484	2.04083065064801
C	14.81045619939920	-2.85010321639610	5.65854118001913
H	16.57493364587910	-1.94390138402737	5.02112570010209
C	8.53540570336116	10.06984841451030	-8.52173392500911
H	9.58995115723853	8.61202483061669	-9.57281506191746
C	-3.03362286682096	1.29433952684497	-15.47145118462680
H	-4.97595738009631	0.74762637476397	-14.95732628691600

C	-5.88462136121376	-10.54353887339040	6.57346514186709
C	-6.47396618140084	-12.76517619350410	7.92534028625753
H	-8.32508576218267	-12.96966350597640	8.85308925643731
C	-4.69841541239915	-14.72639597393540	8.10955635640484
H	-5.18288264518327	-16.45490781401790	9.16854458733496
C	-2.31587167294394	-14.49218731021540	6.95401104973805
H	-0.92785842834970	-16.04012286431230	7.09934893611497
C	-1.70286958096713	-12.27511981801460	5.63210621562115
H	0.16593069721130	-12.03608126376540	4.74424260022048
C	-3.47883515747985	-10.30839768904620	5.45111627970822
H	-2.96845322825179	-8.56846412995729	4.41866977094979
C	-5.77435565634634	-7.53459148643807	-14.14523593400790
H	-6.68679479750916	-8.96168315164448	-15.35947049804760
C	-3.44445900555714	-3.94333253221325	-11.02071803546740
H	-2.55183981634557	-2.55079000793383	-9.74873023113006
C	5.66955743076913	-4.20595462237819	-14.20434269092850
H	6.55792856587562	-4.04673185385024	-12.32167266447350
C	1.03494784072457	8.61865805435515	-8.82327177842280
H	1.98086769849356	7.64345051569816	-7.23973303524106
C	5.99630411002141	13.91154796883040	-8.56456675993246
H	5.06879039024406	15.44809199431160	-9.62189853672450
C	6.19470426411564	7.61580340986167	-14.97130306146580
H	4.59020746007805	8.91173533092186	-15.26218340844480
C	13.91043318180690	4.46282969710155	-5.93337014527695
H	12.78571675786020	2.84630029762882	-5.24476126450563
C	7.36483016816002	12.04078758527400	-9.85590774114182
H	7.52244054819463	12.09476352191790	-11.93097708434630
C	-14.86598519014160	-4.84325036515658	-6.82952107297664
H	-16.40628088573010	-4.85041631259763	-8.23386376673657
C	-5.08892627065467	-8.48406926256038	15.97539682983390
H	-6.43627125439547	-8.92605094294177	17.50209840485440
C	12.51693372834010	10.52614247822650	-0.58089983855279
H	13.90306842527290	10.90159662905360	-2.08722702783973
C	1.18424623839087	7.61994819866044	-11.28838053224850
C	-2.15256858541648	0.17831942364114	17.71518097688410
H	-0.23469643443261	-0.52619408071578	18.11623797727030
C	0.76568361585349	-10.67657930906540	-9.34715694733284
H	-0.44102651322915	-9.64916924291018	-7.98812827635482
C	3.11611410941832	5.41486570152670	14.81798489912720

H	4.98572150004416	4.52284977523706	14.60370462533510
C	8.78455053473548	8.34458683777871	0.98202939792507
H	7.21057945244050	7.00308975916698	0.70134279661219
C	-17.39620298001240	-3.61076019652969	1.38407295955653
H	-17.71067772730880	-4.15536943569218	-0.60152262168540
C	5.79247087738548	13.79464441260280	-5.92257846085870
H	4.70036864160221	15.23582257631450	-4.88769200879386
C	-18.08282847344920	4.82722016263946	-2.12461066186484
H	-19.96100300725710	5.48016344183381	-1.50295208481190
C	-9.97817006318208	12.36572851002150	-2.17901416098644
H	-10.60132666373710	13.73831768328740	-0.74070279764941
C	12.42285197716490	-11.58262345496350	-2.00926371103962
H	12.87354912055190	-11.89067866463980	-4.01779087463387
C	3.82560783049445	-13.32460221268320	-12.77726169486530
H	5.04094571409308	-14.35323358441420	-14.12082339479350
C	11.31479254215860	-10.79540509670010	3.12469051405239
H	10.87321984975990	-10.46299374831920	5.13264835115037
C	18.42311629766220	-7.08032370334432	-4.02012287534513
H	19.74004444526720	-8.21875001255319	-5.16622965686728
C	-2.33475233856317	-9.84848910049143	12.56262754786920
H	-1.52494276518964	-11.36381408616700	11.38717506581880
C	10.49330973557160	-4.21053013304347	4.84461690649984
H	8.85773383013987	-4.39243550310828	3.56211452856159
C	6.93968458356722	-2.28283311856745	14.21610525701570
H	9.00225301481150	-2.34069608911185	14.51257670307750
C	-12.17464801670340	-9.86701568593401	9.20646920360615
H	-12.38153862861470	-11.45554364085530	7.87510307471255
C	1.37903203147909	-15.83542374060530	-1.00465929542937
H	-0.07260584880504	-17.17845821815170	-0.34709545533042
C	-11.81917452059320	-5.73609792257658	12.53359846149780
H	-11.69969298039680	-4.07342084367444	13.78080079760900
C	16.24131195735970	4.02329131186944	-7.12243611751837
H	16.91596113488700	2.06656286547707	-7.34873828607614
C	-8.19423984488107	8.95840344681036	-5.85064986527657
H	-7.43414301072356	7.61473999034471	-7.24895523440455
C	2.04457446209583	-5.12887722951435	-16.88541968005970
H	0.05493539386969	-5.69820129457730	-17.11281968156850
C	5.80081602015823	-14.81681257075500	-1.78278243459736
H	7.81071171674100	-15.35642764348510	-1.68711458876338

C	9.04887414825598	9.55810701418925	3.32987982445830
H	7.68655608150247	9.13285477772044	4.84685526763755
C	-10.43811199274760	-11.89535818535730	-0.40662750814280
H	-9.53131069900036	-13.14976158536090	-1.80098326894149
C	2.55894979208759	-14.63602304258000	-10.85152446659010
H	2.77018154753731	-16.70179933982420	-10.67693754221860
C	8.01515922107119	3.86731696119872	-13.02254208333730
H	7.86554242188669	2.19761759640896	-11.77775939464190
C	9.49471522712759	-10.23899406357450	1.28115808614510
H	7.63443175710971	-9.49359537031371	1.86610480880986
C	-14.91773349162590	-1.44447746474667	4.59885389545207
H	-13.28180483485170	-0.27999914349772	5.16807145588005
C	-9.70935157733193	13.15842392781210	5.15233509694827
H	-11.64928083025010	12.54091094867180	5.58569392343472
C	-0.11196859699694	8.85425170943122	-13.27191561680200
H	-0.08305996029530	8.05514205985126	-15.19589269940010
C	10.33518370955950	4.36629579581646	-14.21039902853850
H	11.93268256169350	3.07380697997428	-13.86631950077890
C	-0.26335407018029	7.01032265557798	17.48581413371070
H	-1.05378004320325	7.38427004387839	19.37666601265610
C	-4.03390135868297	-10.43302005661910	14.51953975283030
H	-4.54951100867968	-12.41685133691180	14.89393601957290
C	11.04310931940030	11.26348462224690	3.71989271485329
H	11.25513625901590	12.21861425040010	5.56014487057470
C	-3.15294545475355	-7.62683141018729	-13.75722165940270
H	-2.03399259694785	-9.15149108565550	-14.63171671010780
C	10.25758899623790	-4.99831191754771	7.36772235157215
H	8.44152097355527	-5.78546469295342	8.01860512427668
C	-16.59706657864120	-2.28742151956055	6.47281602625814
H	-16.23393199262120	-1.75727563525559	8.45449172841252
C	12.78045991840110	11.73606076941130	1.76404927885028
H	14.36239319895050	13.05902733396890	2.06711296414259
C	14.87483409698030	-4.17364503947123	-3.73592036718567
H	13.40249696036200	-3.05258002786637	-4.70280395897476
C	0.67781929412599	-13.40788799203380	-1.81285154697522
H	-1.31412453635231	-12.79721608176840	-1.78689694438978
C	17.44490923522780	5.47201068103432	1.66705860510639
H	18.48132386500810	7.26022164930459	1.92978489071465
C	14.24050264090170	-12.12768456659350	-0.15164785452452

H	16.10566464718180	-12.85486044819620	-0.72773947225691
C	10.59049595404080	6.49393541415508	-15.77521706548000
H	12.41331461927500	6.90178435564309	-16.69923772442090
C	12.30847074011650	-4.73642800291784	9.03072001624798
H	12.12505914192940	-5.34544935981231	11.01586638368280
C	1.03535777725872	-13.30397414650440	-9.13120033963051
H	0.05984166872729	-14.31170834464540	-7.59200241032627
C	-16.82882582080680	6.06285000854104	-4.10843422978114
H	-17.71938832435580	7.68982112399251	-5.05788379073949
C	-12.79236479720980	-6.48077211916029	-7.11157045984279
H	-12.69935583601110	-7.77483560679562	-8.74324850347979
C	-1.49305612964116	11.06623739052400	-12.78903951950830
H	-2.49945014910478	12.01237701362330	-14.34984288308860
C	0.36640340620743	1.53416870455338	-18.54750831075930
H	1.10068133722736	1.18420682501191	-20.46530377096620
C	-12.68560505122500	-8.64171483081866	3.13147400931876
H	-13.59255275176450	-7.35074458877206	4.49209456721257
C	-13.58336539575380	-7.66176961454458	12.99463791960250
H	-14.85070425371400	-7.54509473841417	14.64491740467480
C	16.54961458972820	-5.61738655240214	-5.20189145666317
H	16.35321308063870	-5.58506409191024	-7.27582705362178
C	1.94747855225109	2.64762578492118	-16.72717859936840
H	3.89992785761065	3.16313404531941	-17.23145596631650
C	16.93511028424840	-5.65900225931467	0.10179121689504
H	17.08329198129990	-5.70906442036547	2.17768159731276
C	17.20406277891930	1.22324703975871	-0.08713380992830
H	18.06902078629290	-0.31605594863379	-1.18974275440851
C	2.70263808986034	-3.60940717780802	15.43299935734400
H	1.45733774851890	-4.74057247097408	16.66254261544600
C	-18.67281637313740	-3.80106096692927	5.80666396451051
H	-19.98414748279480	-4.48337273212228	7.27551981302446
C	-19.07066227880580	-4.45632530232414	3.26144759613141
H	-20.69728405848340	-5.64666606658427	2.73090098249615
C	14.58314571194640	-3.66129877083030	8.17324892498658
H	16.18814869547770	-3.42478202199199	9.48119245492421
C	3.48640615644442	-4.50685713339307	-19.02475028106080
H	2.61516988275676	-4.61018582550205	-20.91531531285170
C	-13.76165743044730	-9.72308728158968	11.32605541230010
H	-15.16585085857350	-11.22502910887230	11.66618808359180

C	-9.82557150989497	13.10600411363060	-4.72097853234765
H	-10.37856600203640	15.04028264974930	-5.26540319268840
C	-3.49151690479581	1.48282034925303	19.59827760723100
H	-2.61229662098268	1.77827009381788	21.46496172825560
C	18.52178128250710	3.50417393653043	0.25320914727758
H	20.40946317204550	3.73621985467015	-0.59828162037109
C	8.51785555520824	8.11414825099430	-16.15381823487540
H	8.71177970954498	9.79014681001047	-17.37815285227660
C	-7.02473179465078	2.05954414991227	16.72019004042590
H	-8.92443163723581	2.79268380121209	16.27978115976450
C	-8.93114586712722	11.40621349577350	-6.55888245013303
H	-8.77557481458966	12.00652078948940	-8.54857334927412
C	-2.12572007594935	0.86853382072536	-17.93144460465150
H	-3.36275784139201	-0.00224604166707	-19.36424444997270
C	-5.93036407408984	2.41444211830009	19.11170130257880
H	-6.97349732666914	3.43512005269767	20.59977723581890
C	-5.69283630640033	12.53398695265910	2.98837000801537
H	-4.47111706483268	11.42321938204700	1.71082906604531
C	11.17820334523030	-12.35362609161040	-11.25460649547990
H	12.09722342222720	-12.87143423971610	-13.05227941555040
C	3.94306017371974	-16.53591045181350	-0.99176893053036
H	4.50784407974196	-18.42942323333680	-0.32905383100504
C	11.14091599865760	-9.82290575690429	-10.45656895810680
H	12.00541728348050	-8.31295706016984	-11.60247604072260
C	-1.67953288811030	7.64186190268153	15.33007907373330
H	-3.55964451816170	8.50880398851789	15.54380989252050
C	6.01745988282152	-3.74616599592802	-18.76312300129150
H	7.13910807371505	-3.25651754460363	-20.45013748394060
C	9.96180560810650	-9.17334185792035	-8.17104240638443
H	9.92866590310272	-7.16810443291409	-7.59166278764794
C	13.68920681075230	-11.75067954606250	2.41464585037558
H	15.11667827724320	-12.18660545073990	3.86876883309685
C	18.60312019522060	-7.10561082083883	-1.37051498355098
H	20.05588369778810	-8.26839994648116	-0.43181097552440
C	-6.29097628636391	16.19200256562950	5.71271035591413
H	-5.55001251277402	17.93835200300210	6.57486788554805
C	5.32515818441610	-3.64877273521390	15.82592025824020
H	6.11595441688319	-4.77839166495469	17.38867368174690
C	2.13713174319150	5.91099557198286	17.23692137205320

H	3.24579769526975	5.42141936669616	18.93205167538590
C	7.10597759611639	-3.59032572404643	-16.34736249922940
H	9.07829257180536	-2.97164188277357	-16.08805174910600
C	-4.75791221775145	14.75734398497920	4.08581095798527
H	-2.80648615285492	15.35838590657260	3.67777740303994
C	-12.99953232664510	-11.25098784557040	-0.65092274092963
H	-14.12440653684720	-12.00103656999710	-2.23572048177500
C	-14.11217263952120	-9.61855923134418	1.11995387312887
H	-16.11451685890540	-9.07732862131495	0.93863199442933
C	-8.75756783451941	15.38056709266320	6.25191297467380
H	-9.96433201081143	16.48225368120390	7.54459590218688
C	-5.23704937534828	9.68456021810935	11.08705610704070
C	-5.35173728403738	12.26432210375880	11.74841453912620
H	-3.68266851730513	13.48407156410320	11.50746088273060
C	-7.60533634266699	13.29642892030120	12.69614635391660
H	-7.67520733403742	15.31611214201360	13.20553604463830
C	-9.76255660621491	11.77407116706610	12.98415990901120
H	-11.52776803315100	12.59615153633310	13.72779650224490
C	-9.67015982070497	9.21118250672424	12.30030167438230
H	-11.34417606799200	7.98361244157044	12.47475855081970
C	-7.42219420536545	8.17420682251445	11.34840607230150
H	-7.40179307463540	6.16211595963524	10.79568530194280
