# Supplementary material for: Key factors for connecting silver-based icosahedral superatoms by vertex sharing
Source: Commun Chem. 2023 Mar 28;6:57. doi: 10.1038/s42004-023-00854-0 (PMC10050180; doi:10.1038/s42004-023-00854-0)
Supplement: Supplementary file 12 — Supplementary Data 9 [file 42004_2023_854_MOESM12_ESM.pdf]

element	*x*	*y*	*z*
Ag	0.03925674578955	-0.02224347488441	0.01928672001897
Ag	-4.02364038760142	0.65854957851481	3.55956322611616
Ag	4.09316460430287	-0.69137525392527	-3.52713321651874
Ag	-0.83432178197897	0.25527212879372	-5.44172555746350
Ag	4.12880151794508	-3.61223131842872	0.97487658247458
Ag	0.47232871246037	-4.59009679786609	-3.09695224696014
Ag	5.16122229722169	1.83146051169978	1.07212205063304
Ag	-0.73123048805072	-3.48540015343630	4.27835056107730
Ag	-2.92623200732013	-0.78169355990667	8.54789998459164
Ag	2.06603694681830	4.22085476178470	-2.90712057916528
Ag	2.84256883814738	-3.44134022018505	-7.90235215057796
Ag	3.63421055636898	2.16406897643249	-7.97612201136833
Ag	7.41479623784408	3.44734483478018	-3.92210461352370
Ag	-4.35731605154158	-3.39237582381547	0.03336394812085
Ag	-4.91668902095351	1.86437683067900	-1.57856332070304
Ag	-6.34461193102325	-3.85349553709516	5.13216506517820
Ag	0.95966524771040	1.70358128622496	5.19935604362702
Ag	-6.97446300858904	4.99736340007952	2.76818320110740
Ag	8.01027611021822	-1.32522023215239	-6.95555164597597
Ag	-1.62602553346768	5.02159310702986	1.58312180813948
Ag	-8.87718529864160	-0.19720060867436	1.61354778451217
Ag	6.00315922275872	-5.65365156134541	-3.76888947387005
Ag	-3.28738314830599	4.65298713896857	7.02892118246394
Ag	-7.92049089181986	1.30290756362273	6.99631210088366
Ag	8.87182085365233	-1.41541085085062	-1.37297671361977
Br	-3.64879996037240	-7.37187442044230	-2.93533185675332
Br	3.07880772046755	-6.67513975611036	4.78824274561472
Br	5.55640625827575	3.53780669872844	5.75456620677934
Br	0.49987974137231	8.62025940530147	-1.14089571763475
Br	-5.44584581453031	1.86931886081165	-6.55460580815798
Br	-11.34347824483080	1.89540339457176	10.28885028382760
Br	11.53578295366960	-1.97018587743493	-10.12824841569890
P	-1.81496260649682	-2.20059709226263	12.87987848074250
P	7.63803690914908	-10.06109516758390	-3.54864167126441
P	9.92451609432873	7.43129722996151	-4.18475882615546
P	-13.10461945127080	-0.79298391370397	-0.34193810520686
P	2.81800098005603	4.60863079339557	-11.90258512738830
P	1.52730079068450	-6.02434303146871	-11.59705153854510

P	-2.31752205322587	8.09477457078303	10.09338714259720
P	13.04703201771430	-1.95816388059298	0.68368589239831
P	-8.32572255156237	-8.04593948532515	5.98037131867839
P	-9.33455942620225	8.96973139156072	1.93728785509961
C	-13.32337652295240	3.25920732163021	-3.74241853046335
H	-11.46048082843840	2.57151370844018	-4.38465389216603
C	2.19053603846600	-9.44108357258412	-11.36470054398170
C	1.61452820922004	-2.30408738271169	13.47426694635730
C	-1.63372766104520	2.32770746757148	-13.62746526849200
H	-2.34333937218573	2.69182426266881	-11.69839838417800
C	5.24788078470130	-12.40593247858620	-2.57893534711235
C	-8.38744323413251	8.28117998590620	-3.25324588413184
H	-7.84658624518217	6.33851618989331	-2.71505235964297
C	-14.59671088720100	2.02701382154047	-1.74376952755993
C	13.77405092079700	3.00485591690134	2.34575610359887
H	11.86733603257260	2.80025779774118	3.17153145621295
C	13.01427932040300	6.97819491189848	-5.71326969819944
C	8.42940512138629	10.01702761988400	-5.99387126360411
C	-0.13691076572379	10.51618826430310	8.85461310749926
C	0.33675563115397	10.63563297400620	6.23773960923620
H	-0.60963638093927	9.30004132930787	4.94450153638213
C	2.04543961868458	12.41054388544270	5.25339130960628
H	2.41350368158807	12.43941683968190	3.20350883855369
C	3.28906489997813	14.09520238896910	6.88295779222964
H	4.63650689825475	15.48806896124780	6.11734962492041
C	2.83957124201084	13.98256922638490	9.49955426902157
H	3.83009769383730	15.28570323679410	10.78821259256040
C	1.15245788961697	12.19206928155530	10.48821293979740
H	0.86798771966860	12.07570628897220	12.54878931004380
C	10.50574207476780	8.90900404746735	-1.07668492713926
C	-1.63090705231890	12.05177832858290	-10.32424918133000
H	-2.69891644535991	13.79583986467050	-9.92596718706104
C	10.18327160019460	-10.64688621375970	-1.23661115002437
C	15.11125647730780	5.26337764412994	2.69745593530713
H	14.23960661123360	6.81469086543682	3.77743473585005
C	-10.95112390476510	-6.46729434263680	-5.25222003941950
H	-9.34257340520274	-7.78154049970238	-5.39863062022009
C	1.71890710876069	6.00597445431567	12.80387483652130
H	2.54886825877670	5.66028156301516	10.92024234773790

C	-10.28123098583030	-9.32523299250842	3.38423991162933
C	2.66874662255470	-11.74928120716230	-2.65586328536949
H	2.09466072015162	-9.85004427011854	-3.30192760126109
C	-7.19082016378841	-5.92943091652594	-12.87938234456090
H	-9.25759959727655	-5.91650990567583	-13.14451746000050
C	14.40288286072880	9.00328831879437	-6.76837935816046
H	13.62648162735250	10.93574959547490	-6.74505302122442
C	8.97219267519875	-11.07869751995130	-6.59598047558490
C	-13.06917893510520	-3.03318500055133	-3.01104326549853
C	-0.20399729306148	10.90080929987510	-8.40638912445321
H	-0.15147908465151	11.70999982504880	-6.48891196433499
C	3.78435686913341	-10.71009917964730	-13.08045069617800
H	4.74449338726569	-9.65648447040635	-14.59686390312040
C	7.15594022244074	12.00501351343780	-4.75157684325947
H	7.06208460718894	12.05424752678100	-2.67320012814611
C	0.85797852976830	3.01480685028652	-14.30166219452760
C	-4.52759897405352	-6.04397968578260	15.54430678661770
H	-5.31961020252093	-4.52713588791589	16.72890787834560
C	-1.90742775897413	-5.98902486761150	-12.17456470754330
C	-14.97265945019670	-3.01015171675653	-4.88472319959821
H	-16.52408215567750	-1.62321701416086	-4.78767703209956
C	16.75881142570140	8.55314392453668	-7.89803761315535
H	17.82924891016110	10.14227774303470	-8.71517644581213
C	-2.82650321405329	-5.46149388916270	13.57796728942320
C	-10.43799221780460	-8.00453555897029	8.74132704277333
C	3.12229798411456	-5.02786033558899	-14.52308508749330
C	14.87317766545570	0.99561936691325	0.97258501564343
C	-6.03623251056924	-4.08868713012420	-11.35741058844320
H	-7.17326872360472	-2.63285011328823	-10.39529208946520
C	-3.27621623091232	-0.30373480842439	15.40151305867010
C	-16.96902607340460	2.93553045971304	-0.94206189240333
H	-17.97214163354520	2.00629106636077	0.62629418383301
C	-11.05918114711920	-4.76814395499856	-3.21940898784463
H	-9.53313838451747	-4.77973100329011	-1.79576529063349
C	5.86937230975463	-0.94031866902901	12.30483801633930
H	7.11098457876704	0.16039057422681	11.04515763460690
C	3.24487575338737	-0.87857806999925	11.92375042536080
H	2.46277789163608	0.27538734387478	10.37060197591090
C	12.85098503011260	-3.05258366579175	3.98465450613496

C	10.24094574881460	-14.28359829072260	-9.65176488494060
H	10.29266025372890	-16.27955846295380	-10.24649040571290
C	-10.17434820894030	-6.02188216725897	10.50356980583990
H	-8.76263413027378	-4.51973765813618	10.18221202425390
C	-9.26707935482102	-11.08440038471950	1.65344028280011
H	-7.30278947418010	-11.74262041085150	1.85429779306049
C	-0.69452366500732	7.12716552128751	13.02552270849230
C	-1.83274465916578	-7.42941312933724	12.07002914584820
H	-0.50626276620471	-7.00072384475735	10.51657039509030
C	-9.21571769754897	9.99462146686270	-1.39011838494477
C	9.04207016707717	-13.62677906611180	-7.37820494766276
H	8.15234478441556	-15.10959879880070	-6.21958684248874
C	-12.67058906126180	8.69352322727086	2.87969133867481
C	-14.64562687682150	10.00716906608140	1.66434063264498
H	-14.25867988196360	11.17000957954240	-0.01810403135021
C	-17.12498205523660	9.82613693478674	2.59238053531406
H	-18.65762985393870	10.86390373036420	1.63638911233278
C	-17.65608691083790	8.33351533742040	4.72390298009599
H	-19.60636646345850	8.20458273400130	5.44536572842882
C	-15.70068524320620	6.99776520237604	5.92381152162758
H	-16.08200649264720	5.79696364659367	7.58223564942187
C	-13.21986192028490	7.17262945431692	5.00272081941025
H	-11.70819261078430	6.10631817228657	5.96926447600869
C	-5.69619722810796	0.71570227676741	14.93266316599130
H	-6.60268195768964	0.50562084895157	13.06373400589830
C	17.74057747679930	6.08678441238203	-8.00949680159205
H	19.58268828329180	5.74099854662320	-8.91864400904865
C	5.75947781412898	5.45075235268593	-13.56138928442000
C	-14.43843175882210	5.33717575111883	-4.95138681747388
H	-13.43325136435130	6.28055057741873	-6.51155164691670
C	15.16737612575530	-4.10512941824209	-1.04601376731447
C	-8.18658148233072	11.80698046065850	3.61080605610787
C	-15.42499681885980	-1.97393354729964	1.97080038802196
C	14.87067370186990	-2.69843406080973	5.69681725089517
H	16.61605403674120	-1.74598432058565	5.07547741682577
C	8.51384133278805	9.98732919255861	-8.66295017581090
H	9.48546229031218	8.44571041271441	-9.67460462178141
C	-3.23749241523635	1.21266055122898	-15.41785581516880
H	-5.17913022293578	0.69243495052315	-14.87533971610310

C	-5.99144395710717	-10.59006949456560	6.49312012706615
C	-6.58535534566270	-12.81146786749510	7.84338596318232
H	-8.43316405645133	-13.01102963880280	8.77944093348590
C	-4.81693270659975	-14.78065462676810	8.01087623040969
H	-5.30257672135564	-16.51084397926700	9.06464850520954
C	-2.44048841555922	-14.55225878957770	6.84299181996657
H	-1.05966953041826	-16.10665309914970	6.97767145079746
C	-1.82628724188455	-12.33654504539030	5.51778825380406
H	0.03626301738778	-12.11846956804220	4.61162012842241
C	-3.59489168114354	-10.36291794682590	5.34985772088134
H	-3.09297047902697	-8.62952074008721	4.30206244589328
C	-5.71403109395115	-7.80984462487180	-14.03893012580010
H	-6.61656212633685	-9.27072249017335	-15.21823893194100
C	-3.40546814976904	-4.11669584333818	-11.01793276179120
H	-2.51588030364797	-2.67467948329394	-9.80000686256118
C	5.63822871800715	-4.16152680665086	-14.31778066532300
H	6.56261344440933	-3.99100076634684	-12.45378947875650
C	1.14316456803166	8.67346581060319	-8.91372397192568
H	2.24261929926251	7.77297784906008	-7.38567272703400
C	6.15815897845901	13.94032925031040	-8.80588088574864
H	5.28749471153274	15.48049905114990	-9.90448316385140
C	5.92838099085032	7.54379589198641	-15.20706709660060
H	4.28412401950918	8.78945885129393	-15.49571052061850
C	14.00818038966250	4.50873979343297	-5.84753251331946
H	12.91823812137990	2.90327839202426	-5.08135135335357
C	7.39807199525936	11.95170062887190	-10.05126720677500
H	7.50811231111575	11.91729630025010	-12.12941950017060
C	-14.87163711078150	-4.72515175891196	-6.90443367040813
H	-16.36702590571060	-4.69524458879479	-8.35480723574824
C	-5.20469268652037	-8.56957056744207	16.01077865203300
H	-6.53170555251814	-9.00856509768130	17.55484896469780
C	12.53789449501550	10.58500974656480	-0.65940454031922
H	13.93788369487040	10.93707548263650	-2.15896160442241
C	1.06929410549028	7.56952107784017	-11.33759328843510
C	-2.15899285652271	0.02175177177409	17.79941617239990
H	-0.25540052272907	-0.72797050395608	18.18643617448960
C	0.98658388974333	-10.82063742418170	-9.42100845379914
H	-0.26800413992700	-9.84775650533321	-8.06560123930070
C	3.09623065948965	5.37269281437440	14.97716896065180

H	4.98032999609774	4.50674311656191	14.78632950414010
C	8.76341833071200	8.46034722541590	0.88912241983235
H	7.16677203682570	7.14807444806890	0.59707091794355
C	-17.55793205249930	-3.41272057270320	1.26367505442367
H	-17.86928826290400	-3.91658031889144	-0.73313413353240
C	6.02784549731852	13.95271102013100	-6.15674012005960
H	5.05537366246645	15.50360760705500	-5.16231142176378
C	-18.07324524267140	5.02077599251781	-2.16064455221775
H	-19.93604427099170	5.70619572928098	-1.53063020131392
C	-9.83242804326481	12.49456494330840	-2.10371943992254
H	-10.40916529597550	13.88429285400340	-0.66308788247069
C	12.59434500067860	-11.52132529235290	-1.95387899215443
H	13.05370905880570	-11.80306803138800	-3.96402862310815
C	4.13267870652765	-13.33439144109470	-12.87561980706370
H	5.37090901075400	-14.31304733454400	-14.23432093009970
C	11.45933168481060	-10.82300733749260	3.19018228073385
H	11.00839286565940	-10.53831398776750	5.20315410557152
C	18.55729266119270	-7.01205284287824	-3.95269556932051
H	19.89381954699220	-8.14028727339880	-5.08456892336347
C	-2.50135635130452	-9.94502954591953	12.55914143548600
H	-1.71211502721315	-11.46525587442610	11.37636709390160
C	10.60182217769790	-4.18985550694652	4.84553552007986
H	8.98976162246423	-4.43248941788327	3.54338711362350
C	6.88555577343564	-2.42579597052967	14.25503807955480
H	8.94646797480441	-2.48020131150929	14.55876457001210
C	-12.31088472405240	-9.86836471256631	9.14570832066321
H	-12.59605293057250	-11.39340444489630	7.75616953191840
C	1.51699791957364	-15.88165632897470	-1.01190831303005
H	0.06334167322563	-17.23816028102070	-0.38941710181694
C	-11.72982724025590	-5.91020128311008	12.64950724919450
H	-11.51916900680530	-4.32441875461287	13.98153811809500
C	16.35934326689730	4.06407502429675	-6.99176020594452
H	17.08729473906570	2.11713859591086	-7.11346989459622
C	-8.19145903505674	9.03081435305460	-5.79065454356024
H	-7.49603162790987	7.66942705015706	-7.20503050845304
C	1.98793697911916	-5.20020806845007	-16.92884111407690
H	0.01471961043070	-5.83556469318387	-17.11927427321020
C	5.94748860752666	-14.81949710766980	-1.67190843786261
H	7.95862467268413	-15.33901828232620	-1.51818639509651

C	9.02644148023430	9.68404780007350	3.23100080860682
H	7.64655675557624	9.29999019396523	4.74318445646608
C	-10.76406921081790	-12.03185522883270	-0.32095308687387
H	-9.95214046715230	-13.41650146597430	-1.64872677113966
C	2.90559453889321	-14.70360253501380	-10.96467002846130
H	3.17264986251089	-16.76440789691610	-10.81934264908150
C	7.89439478487895	3.89499935139492	-13.20049105385450
H	7.81375240228679	2.25214296629836	-11.91510901174180
C	9.63351091613137	-10.27520487388860	1.35006583618239
H	7.75287009198044	-9.58783817836554	1.93938378220746
C	-15.05522119567660	-1.36248038956700	4.54051088135612
H	-13.40110984248130	-0.24221378924260	5.14348266531504
C	-9.72363689600488	13.20643564116150	5.27644746872712
H	-11.66541667915180	12.58141651565720	5.68942431152657
C	-0.38937539187590	8.72572814344752	-13.25373657092690
H	-0.51854584494491	7.85570525920988	-15.14180623811470
C	10.16777215800540	4.41624387010462	-14.46619258831670
H	11.81090550355140	3.17786366827790	-14.14113800772800
C	-0.33476475729510	6.91781211562423	17.61165585432000
H	-1.14906007795009	7.27705774491699	19.49430915814450
C	-4.18780935519250	-10.52011542168430	14.53009092549600
H	-4.71542659545912	-12.49982110130600	14.90597906244010
C	11.04090447710810	11.36570590907580	3.62530839852333
H	11.25237412045790	12.33020119547520	5.45997725993716
C	-3.08797298805461	-7.85798328576250	-13.67558115825900
H	-1.95708554985142	-9.38128795288105	-14.53583487055980
C	10.36957087103310	-4.98604676435838	7.36544195215022
H	8.57961208291056	-5.83626357781397	8.00830267390588
C	-16.78569182942760	-2.17729060377963	6.38012079724744
H	-16.44697774478430	-1.68231854655506	8.37483760439323
C	12.79702317421790	11.80696335224010	1.67992826018704
H	14.38910169911740	13.11549445554900	1.98589889523536
C	14.97645793716980	-4.14174697721630	-3.70761985878419
H	13.50432468904280	-3.03287093534235	-4.68783059044816
C	0.80988889592968	-13.47285816704880	-1.87151357404789
H	-1.19251527424476	-12.89957736854310	-1.91426122370135
C	17.53880679289820	5.55238232074348	1.65729903888270
H	18.58508812188450	7.33270416237902	1.92916424290235
C	14.41524684288480	-12.06132300328210	-0.09812268073063

H	16.28945893645030	-12.76012284064400	-0.67703298584396
C	10.32550501822630	6.50050560056087	-16.10110372474820
H	12.10867155339710	6.92018381066837	-17.09289705129180
C	12.39829215040790	-4.66272013178401	9.04544394115195
H	12.22128751878800	-5.28516131089577	11.02630113787100
C	1.33900078745745	-13.43996775297670	-9.23115524755567
H	0.38598359016128	-14.49903662501170	-7.71259868583238
C	-16.82110204590910	6.21511472940721	-4.17056083881424
H	-17.69995354711070	7.84110810754551	-5.13099884591631
C	-12.86232893543480	-6.45255587041883	-7.09213707791108
H	-12.77744130409050	-7.78129551191259	-8.69523781498834
C	-1.71774720811727	10.96460033708710	-12.74655758943040
H	-2.84620991954516	11.85417064897850	-14.25493964544950
C	0.11582879546669	1.39962604789465	-18.55080413873290
H	0.81214513474794	1.03503292575803	-20.47902176142590
C	-12.80059821174930	-8.49603445294232	3.07626676632833
H	-13.62866691280260	-7.10997404276488	4.39350171136834
C	-13.56314963262430	-7.78393037153544	13.05496083738480
H	-14.79264633232050	-7.69611085791370	14.73431271580790
C	16.66927632783720	-5.58372762346330	-5.15411506716258
H	16.48957900207290	-5.57714881802883	-7.22940701873241
C	1.73328953341824	2.52294156097345	-16.76895880695480
H	3.67695960251024	3.02872706113616	-17.31382559115290
C	17.04603837097300	-5.56806513695520	0.15240525297550
H	17.19626499216120	-5.59185428941870	2.22850575587117
C	17.30209183581500	1.29573238213220	-0.07790615792794
H	18.17710552560650	-0.25444831398376	-1.15677416239995
C	2.65435926026712	-3.82485222894202	15.40812279234260
H	1.41440584692841	-5.00675068468166	16.59378457640220
C	-18.89870526146700	-3.61591069474602	5.66606730366584
H	-20.25493083832950	-4.26803932144923	7.10645009067969
C	-19.28308097957030	-4.22696825099407	3.10802339493396
H	-20.94291907107710	-5.35115251455803	2.54116925677492
C	14.64601487619350	-3.51856813112690	8.20869152045014
H	16.23371313241110	-3.24087212656731	9.52809142095717
C	3.37176313015277	-4.54633871152486	-19.09647175424340
H	2.47367708982336	-4.69253746533853	-20.97056726070900
C	-13.85323710640350	-9.75801700373444	11.29978872825180
H	-15.30699353329720	-11.21908279408080	11.60148558648090

C	-9.66556558760385	13.23303850983110	-4.64522756951507
H	-10.15708113792140	15.18438360459060	-5.18388428873243
C	-3.47290316426558	1.31279812684573	19.70877634053560
H	-2.59007439354323	1.55175290940901	21.58086992581290
C	18.62402073543060	3.57369631047684	0.26526323597063
H	20.52501155733510	3.79166810239460	-0.55784209774300
C	8.20441723619573	8.05917667272120	-16.46989707675570
H	8.32251985182928	9.69629076236323	-17.75339705101790
C	-7.00297893385050	2.00175947500702	16.84918341411520
H	-8.89443400700613	2.77250365463202	16.44062893582580
C	-8.84275617632718	11.50598536070450	-6.48979996696342
H	-8.68291829247502	12.10297477130770	-8.47935223831212
C	-2.36950962798802	0.75507919603057	-17.88672442979690
H	-3.63654816370225	-0.11349072812778	-19.29342803518600
C	-5.89631279151426	2.29398573216332	19.24376910713200
H	-6.92118775816527	3.29833755801596	20.75429352827990
C	-5.69244822700010	12.62506935526230	3.12063235577992
H	-4.46784212729355	11.54317120253340	1.82181583022615
C	11.36699424295850	-12.41342507361600	-11.16520686129330
H	12.30766014485760	-12.94388318878940	-12.94692361160600
C	4.08689985423874	-16.55014436197070	-0.91354030931710
H	4.65355110117623	-18.42966025815860	-0.21642721552439
C	11.27624410649200	-9.87032554501321	-10.41176756626150
H	12.12318608213180	-8.37147793114740	-11.58408240680190
C	-1.72606375058007	7.55931564036952	15.44278007208670
H	-3.61167291468315	8.41756716395181	15.63979778192860
C	5.88334127891981	-3.70965573316298	-18.88441550180750
H	6.96020130349688	-3.20289658131358	-20.59433809318300
C	10.07876704898110	-9.20522248340795	-8.14045483001791
H	10.01855170186070	-7.19387961312222	-7.58558803683225
C	13.85085938776190	-11.72780568805350	2.47120453809932
H	15.27846973618290	-12.17006700374420	3.92223752550905
C	18.73364791421550	-7.00868261971072	-1.30293120101782
H	20.20314606736170	-8.13829915681990	-0.35185285712275
C	-6.32192350938665	16.25017472779590	5.87985392488390
H	-5.59842935027844	17.99707472047230	6.75398794904822
C	5.27666198587610	-3.86708273280520	15.80217941589440
H	6.07019401096544	-5.05267949118894	17.32016201566380
C	2.07678652892228	5.83815824935704	17.38566201477270

H	3.16787364794749	5.35072508985260	19.09186537678590
C	7.01336768694330	-3.51129310757717	-16.49054023042240
H	8.97507180177070	-2.84280287072718	-16.28394110828000
C	-4.77289990514763	14.84068507871140	4.24591800668483
H	-2.82747646001379	15.46568748305060	3.84599590991587
C	-13.28108755176820	-11.24001228571240	-0.59050670686126
H	-14.45984892068880	-12.00007337676160	-2.13004119457022
C	-14.28867206392230	-9.46824710213043	1.10847432439456
H	-16.25906748758090	-8.82685193376743	0.91173953166372
C	-8.78710360057706	15.42274301390920	6.40027220909223
H	-10.00757668046090	16.51259024396510	7.68887261068328
C	-5.20529808813393	9.70365039967447	11.17770622886780
C	-5.27004776230747	12.27872136760760	11.86479545739710
H	-3.57753712172401	13.46989772851890	11.64677535649370
C	-7.50601713531598	13.34012846889310	12.82066138667520
H	-7.53910608291047	15.35225167114010	13.35897348582790
C	-9.69025193108654	11.85352518825000	13.08946469010560
H	-11.43839085972970	12.69779109315080	13.84613595687950
C	-9.64454425968572	9.29616510661460	12.38000282300260
H	-11.34398332530700	8.10445821290019	12.55204528010960
C	-7.41456018755073	8.22654361490814	11.42239937477820
H	-7.42214533837893	6.21513713926199	10.86803271332600
